# The phylogenetic nomenclature of ornithischian dinosaurs

**DOI:** 10.7717/peerj.12362

**Published:** 2021-12-09

**Authors:** Daniel Madzia, Victoria M. Arbour, Clint A. Boyd, Andrew A. Farke, Penélope Cruzado-Caballero, David C. Evans

**Affiliations:** 1 Department of Evolutionary Paleobiology, Institute of Paleobiology, Polish Academy of Sciences, Warsaw, Poland; 2 Department of Knowledge, Royal BC Museum, Victoria, BC, Canada; 3 School of Earth and Ocean Sciences, University of Victoria, Victoria, BC, Canada; 4 North Dakota Geological Survey, Bismarck, ND, USA; 5 Raymond M. Alf Museum of Paleontology at The Webb Schools, Claremont, CA, USA; 6 Área de Paleontología, Departamento de Biología Animal, Edafología y Geología, Universidad de La Laguna, Santa Cruz de Tenerife, Spain; 7 Instituto de Investigación en Paleobiología y Geología (IIPG), Universidad Nacional de Río Negro (UNRN), Río Negro, Argentina; 8 Instituto de Investigación en Paleobiología y Geología (IIPG), Consejo Nacional de Investigaciones Científicas y Tecnológicas (CONICET), Río Negro, Argentina; 9 Grupo Aragosaurus-IUCA, Área de Paleontología, Departamento de Ciencias de la Tierra, Universidad de Zaragoza, Zaragoza, Spain; 10 Department of Natural History, Royal Ontario Museum, Toronto, ON, Canada

**Keywords:** Phylogenetic nomenclature, Phylogenetic definition, *PhyloCode*, *International Code of Phylogenetic Nomenclature*, *Ornithischia*, *Dinosauria*

## Abstract

Ornithischians form a large clade of globally distributed Mesozoic dinosaurs, and represent one of their three major radiations. Throughout their evolutionary history, exceeding 134 million years, ornithischians evolved considerable morphological disparity, expressed especially through the cranial and osteodermal features of their most distinguishable representatives. The nearly two-century-long research history on ornithischians has resulted in the recognition of numerous diverse lineages, many of which have been named. Following the formative publications establishing the theoretical foundation of phylogenetic nomenclature throughout the 1980s and 1990s, many of the proposed names of ornithischian clades were provided with phylogenetic definitions. Some of these definitions have proven useful and have not been changed, beyond the way they were formulated, since their introduction. Some names, however, have multiple definitions, making their application ambiguous. Recent implementation of the *International Code of Phylogenetic Nomenclature* (*ICPN*, or *PhyloCode*) offers the opportunity to explore the utility of previously proposed definitions of established taxon names. Since the Articles of the *ICPN* are not to be applied retroactively, all phylogenetic definitions published prior to its implementation remain informal (and ineffective) in the light of the Code. Here, we revise the nomenclature of ornithischian dinosaur clades; we revisit 76 preexisting ornithischian clade names, review their recent and historical use, and formally establish their phylogenetic definitions. Additionally, we introduce five new clade names: two for robustly supported clades of later-diverging hadrosaurids and ceratopsians, one uniting heterodontosaurids and genasaurs, and two for clades of nodosaurids. Our study marks a key step towards a formal phylogenetic nomenclature of ornithischian dinosaurs.

## Introduction

The ornithischian, or ‘bird-hipped’, dinosaurs were a species-rich clade of Mesozoic archosaurs that first appeared in the Triassic (*e.g*., Langer & Ferigolo, 2013; Cabreira et al., 2016; Pacheco et al., 2019; Desojo et al., 2020; Müller & Garcia, 2020) or the earliest Jurassic (Agnolín & Rozadilla, 2018; Baron, 2019) and died out during the Cretaceous/Paleogene extinction event (*e.g*., Brusatte et al., 2015). Throughout their >134 million-year-long evolutionary history, ornithischians achieved global distribution (Weishampel et al., 2004; Boyd, 2015), evolved considerable taxic diversity (Tennant, Chiarenza & Baron, 2018), and an apparent morphological disparity, expressed through their markedly different body sizes (Benson et al., 2018) and especially the ‘exaggerated’ structures of the crania and osteodermal armor of some of their most distinctive members (*e.g*., Brown, 2017; Stubbs et al., 2019).

Here, we provide a nomenclatural revision of ornithischian dinosaur clades. Following the pivotal, early formative publications establishing the theoretical foundation of the phylogenetic nomenclature in the 1980s and early 1990s (*e.g*., Ghiselin, 1984; Gauthier, 1986; Rowe, 1987; de Queiroz, 1988; Estes, de Queiroz & Gauthier, 1988; Gauthier, de Queiroz & Estes, 1988; de Queiroz & Gauthier, 1990, 1992, 1994), many names of the ornithischian clades were provided phylogenetic definitions (*e.g*., Padian & May, 1993; Currie & Padian, 1997; Sereno, 1998; Sereno, 1999), some of which have proven useful and have not been changed, beyond the way they were formulated, since their introduction.

The implementation of the *International Code of Phylogenetic Nomenclature*, or the *PhyloCode* (de Queiroz & Cantino, 2020), an evolution-based system for naming organisms, hereafter abbreviated and referred to as *ICPN* (accessible at http://phylonames.org/code/), and parallel publication of *Phylonyms: A Companion to the PhyloCode* (de Queiroz, Cantino & Gauthier, 2020), offers the opportunity to consider the utility of previously proposed phylogenetic definitions of established taxon names and, in appropriate cases, formalize their use, as specified by the Articles of the *ICPN*.

Recent studies have thoroughly assessed the use of clade names applied to some ornithischian lineages, mostly early-diverging neornithischians and ornithopods (Boyd, 2015; Madzia, Boyd & Mazuch, 2018; Herne et al., 2019; Madzia, Jagt & Mulder, 2020). However, the Articles of the *ICPN* are not to be applied retroactively (*ICPN*: Preamble 6, see also Art. 7.1). As such, all these efforts remain informal and ineffective in the light of the Code.

We formalize some of the nomenclatural acts of previous studies and introduce phylogenetic definitions for 81 names of ornithischian dinosaur clades. Specifically, we provide formal phylogenetic definitions for the following 76 preexisting taxon names: *Ankylopollexia*, *Ankylosauria*, *Ankylosauridae*, *Ankylosaurinae*, *Ankylosaurini*, *Aralosaurini*, *Brachylophosaurini*, *Camptosauridae*, *Centrosaurinae*, *Centrosaurini*, *Cerapoda*, *Ceratopsia*, *Ceratopsidae*, *Ceratopsoidea*, *Chaoyangsauridae*, *Chasmosaurinae*, *Clypeodonta*, *Coronosauria*, *Dryomorpha*, *Dryosauridae*, *Edmontosaurini*, *Elasmaria*, *Eucentrosaura*, *Euhadrosauria*, *Euiguanodontia*, *Euornithopoda*, *Eurypoda*, *Genasauria*, *Hadrosauridae*, *Hadrosauriformes*, *Hadrosaurinae*, *Hadrosauroidea*, *Hadrosauromorpha*, *Heterodontosauridae*, *Huayangosauridae*, *Hypsilophodontia*, *Hypsilophodontidae*, *Iguanodontia*, *Iguanodontidae*, *Jeholosauridae*, *Kritosaurini*, *Lambeosaurinae*, *Lambeosaurini*, *Leptoceratopsidae*, *Marginocephalia*, *Nasutoceratopsini*, *Neoceratopsia*, *Neoiguanodontia*, *Neornithischia*, *Nodosauridae*, *Nodosaurinae*, *Ornithischia*, *Ornithopoda*, *Orodrominae*, *Pachycephalosauria*, *Pachycephalosauridae*, *Pachycephalosaurinae*, *Pachycephalosaurini*, *Pachyrhinosaurini*, *Pachyrostra*, *Parasaurolophini*, *Polacanthinae*, *Protoceratopsidae*, *Rhabdodontidae*, *Rhabdodontomorpha*, *Saurolophinae*, *Saurolophini*, *Shamosaurinae*, *Stegosauria*, *Stegosauridae*, *Styracosterna*, *Thescelosauridae*, *Thescelosaurinae*, *Thyreophora*, *Triceratopsini*, and *Tsintaosaurini*. These names cover all major ornithischian clades and the vast majority of their subclades for which taxon names were used and defined in the past. Additionally, we introduce five new clade names: *Corythosauria*, for the node uniting lambeosaurin and parasaurolophin lambeosaurines, *Euceratopsia*, for the node uniting leptoceratopsid and coronosaur ceratopsians, *Saphornithischia*, for the node uniting heterodontosaurids and genasaurs, and *Panoplosaurini* and *Struthiosaurini* for clades of later-diverging nodosaurids.

## Methods

### Protocol

In order to be formally established under the *ICPN*, clade names must comply especially with the provisions of Articles 7 and 9–11 of the Code (*ICPN*: Art. 7.2d). These Articles are fully followed here. The entries, provided in ‘Phylogenetic nomenclature of ornithischian clades’ below, partly follow the scheme used in *Phylonyms* (de Queiroz, Cantino & Gauthier, 2020); they include the following sub-sections: ‘Definition’, ‘Reference phylogeny’, ‘Composition’, ‘Synonyms’, and ‘Comments’. The sub-sections ‘Diagnostic apomorphies’ and ‘Etymology’, as used in *Phylonyms*, have been omitted. Note that detailed discussion of apomorphies is not strictly required by the Code, and inclusion of a reference phylogeny alone is sufficient (*ICPN*: Art. 9.13). Recent assessments of the phylogenetic relationships of numerous taxa, particularly those nested near the basal neornithischian-ornithopod transition, but also within some major clades, such as ornithopods, currently provide conflicting results (*e.g*., Norman, 2015; Han et al., 2018; Madzia, Boyd & Mazuch, 2018; Andrzejewski, Winkler & Jacobs, 2019; Herne et al., 2019; Párraga & Prieto-Márquez, 2019; Dieudonné et al., 2020; Yang et al., 2020; Barta & Norell, 2021; Černý, Madzia & Slater, 2021). It is extremely difficult, and perhaps impossible at the moment, to list unambiguous diagnostic apomorphies for many clades that have long been associated with widely-used names, and detailed discussion would be far beyond the scope of the paper. Instead, emphasis was placed on using definitions that are reflective of all currently inferred phylogenies. In turn, ‘Etymology’ was omitted because all but five of the clade names that are established in the present study are preexisting (Art. 6.2 of the *ICPN*). The only reason for discussing the etymological origin of taxon names would be to provide arguments for the inclusion of certain internal specifiers (*e.g*., within the context of Art. 11.10 of the *ICPN*). With that respect, relevant comments are provided in the ‘Comments’ sub-section of the name entries. The five new clade names introduced in the present study are provided with their etymologies. Additionally, owing to the fact that the phylogenetic relationships of ornithischian dinosaurs are intensively researched, each clade name entry could be supplemented with numerous reference phylogenies. Rather than list all of the relevant phylogeny reconstructions available, we decided to refer to a subset of the more recent tree topologies that justify the ‘conversion’ of the taxon name in accordance with the *ICPN*.

We have not followed any strict approach while selecting primary reference phylogenies. Instead of providing references to studies that represent, for example, the most recent iterations of some datasets, we preferred to refer to studies that we have been either directly involved in, and are therefore familiar with the original data used for phylogeny inference, or consider to cover relevant data sampling.

With respect to the clade ‘Composition’, we list only those subtaxa that are included in the primary reference phylogeny. It is essential to realize that some of the clades for which names are provided have insufficiently explored origins and their basal branching is expressed through polytomies (this applies especially, but not exclusively, to non-hadrosaurid ornithopod subclades). In such cases, the actual extent may not be certain and some of the taxa listed in the ‘Composition’ subsection may in fact fall outside the clades. Note also that some of the selected primary reference phylogenies do not show the placements of all taxa used as specifiers in the definitions of the names to be defined. In such cases, the phylogenetic positions of these specifiers are discussed in the ‘Comments’.

We also realize that the list of taxon names provided in ‘Synonyms’ is not exhaustive and does not list all historically used approximate synonyms. When discussing names that may be considered synonymous with those whose application is preferred here, we have focused especially on those names that have been used for the same or very similar contents in recent years, or those that have been used interchangeably with those that we define (*e.g*., *Iguanodontidae* and *Iguanodontoidea*, *Thescelosauridae* and *Parksosauridae*). Therefore, the names that have not been in use for a long time were mostly omitted.

Further, Article 8.1 of the *ICPN* states that, “(i)n order for a name to be established under [the *ICPN*], the name and other required information must be submitted to the registration database for phylogenetically defined names (see Art. 22.2). A name may be submitted to the database prior to acceptance for publication, but it is given only a temporary registration number at that time. The registration number will become permanent after the author notifies the database that the paper or book in which the name will appear has been published, provides a full reference to the publication, and confirms that the definition in the database is identical to that in the publication”. We have therefore registered all names, whose phylogenetic definitions are established in the present study, to the database of phylogenetically defined names, the *RegNum* (*ICPN*: Art. 22; Appendix A), and obtained registration numbers that are included in the clade name entries.

Finally, we follow the *ICPN* in that all scientific names are italicized (*ICPN*: Recommendation 6.1A.) and that names are attributed to the earliest author(s) to spell them rather than according to the Principle of Coordination (*ICPN*: Note 9.15A.3).

### Phylogenetic definitions

The names of ornithischian clades are defined using the following two types of definitions: (a) minimum-clade definition, known previously as ‘node-based’ definition (*ICPN*: Art. 9.5) and (b) maximum-clade definition, known previously as ‘branch-based’ or ‘stem-based’ definition (*ICPN*: Art. 9.6). We refer to the aforementioned Articles of the *ICPN* for details.

**Adopted conventions for abbreviated definitions.** We abbreviate the definitions using the following conventions (as per Notes 9.4.1 and 11.12.1 of the *ICPN*): max = the largest; min = the smallest; ∇ = clade; () = containing; & = and; ∨ = or; ~ = but not (in trivial maximum-clade definitions) or it does not (while using a qualifying clause); | = on the condition that. See also Note 9.6.2 of the *ICPN* for explanation of differences between the use of ‘&’ and ‘∨’ in the definitions. Additionally, we apply the set theory symbols ∈, that means “belongs to”, and ∉, meaning “not element of”, to indicate that a name is applied *within* or *outside* another clade, respectively (see *Euornithopoda*, *Jeholosauridae*, *Orodrominae*, and *Polacanthinae* for some examples).

**Selection of specifiers.** Specifiers are selected following Art. 11 of the *ICPN*. Numerous names pertaining to ornithischian clades have been informally defined in the past and these definitions can still be considered applicable. We have attempted to formalize most of these definitions, providing only the changes that were necessary to reflect all currently inferred phylogenies and to comply with the Articles of the *ICPN*. However, in some cases we have decided to replace certain specifiers with taxa that we consider to be more appropriate candidates. For example, we have replaced *Parasaurolophus walkeri* Parks, 1922 in some definitions with *Iguanodon bernissartensis* Boulenger in Beneden, 1881 (designated as the type species of *Iguanodon* Mantell, 1825 by the International Commission on Zoological Nomenclature (2000)), provided that this taxon has always been considered part of the clade (when selected as an internal specifier) or outside the clade (when selected as an external specifier) whose name is being defined. *I. bernissartensis* is known based on multiple complete or near-complete individuals of different ontogenetic stages and has been extensively researched (*e.g*., Norman, 1980; Verdú et al., 2017). It has also been frequently used as the specifier in previous, informal phylogenetic definitions, and was recently included as the internal specifier of *Dinosauria* (Langer et al., 2020). It is further essential to note that some taxa had to be used as internal specifiers despite their suggested dubious taxonomic status. For example, *Ceratops montanus* Marsh, 1888 is the name-bearer of *Ceratopsia*, *Ceratopsoidea*, *Ceratopsidae*, and *Ceratopsinae* (the last name is not converted to a clade name in the present study). At the same time, however, the taxon is generally considered to lack diagnostic features and is commonly treated as a *nomen dubium* (*e.g*., Dodson, Forster & Sampson, 2004; Mallon et al., 2016). Following Article 11.10 of the *ICPN* (which specifies that “(w)hen a clade name is converted from a preexisting name that is typified under a rank-based code or is a new or converted name derived from the stem of a typified name, the definition of the clade name must use the type species of that preexisting typified name or of the genus name from which it is derived (or the type specimen of that species) as an internal specifier.”), *Ceratops montanus* must be the internal specifier (or among the internal specifiers) in the definitions of the names in question.

## Phylogenetic Nomenclature of Ornithischian Clades

**Figure 1:**
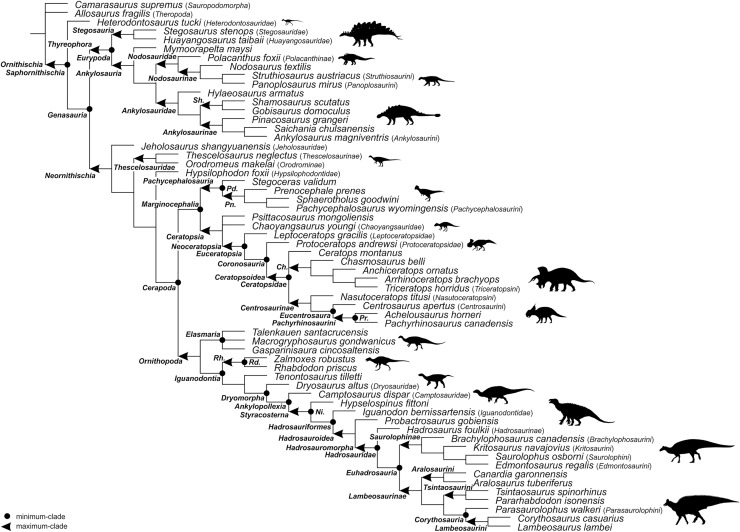
Specifier-based phylogeny of *Ornithischia*. Subclade topologies reflect those of the primary reference phylogenies: *Ankylosauria* (Figure 11 of Arbour & Currie, 2016; Figure 5 of Rivera-Sylva et al., 2018a), *Hadrosauridae* (Figure 25 of Prieto-Márquez et al., 2013; Figure 18 of Prieto-Márquez, Wagner & Lehman, 2020), *Marginocephalia* (Figure 27 of Schott & Evans, 2017; Figure 9 of Chiba et al., 2018; Figure 9a of Fowler & Freedman Fowler, 2020; Figure 10 of Morschhauser et al., 2019; Figure 4 of Yu et al., 2020), non-ankylosaur *Thyreophora* (Figure 16 of Han et al., 2018), non-cerapod *Neornithischia* (Figure 4 of Madzia, Boyd & Mazuch, 2018), non-genasaur *Ornithischia* (Figure 4 of Madzia, Boyd & Mazuch, 2018), non-hadrosaurid *Ornithopoda* (Figure 2.26 of Norman, 2014; Figure 4 of Madzia, Boyd & Mazuch, 2018; Figure 12 of Madzia, Jagt & Mulder, 2020). Abbreviations: *Ch*. – *Chasmosaurinae*; *Ni*. – *Neoiguanodontia*; *Pd*. – *Pachycephalosauridae*; *Pn*. – *Pachycephalosaurinae*; *Pr*. – *Pachyrostra*; *Rh*. – *Rhabdodontomorpha*; *Rd*. – *Rhabdodontidae*; and *Sh*. – *Shamosaurinae*. Majority of the silhouettes were obtained from phylopic.org: *Ankylosaurinae* (Andrew A. Farke, CC BY 3.0), *Camptosauridae* (Tasman Dixon, public domain), *Centrosaurinae* (Andrew A. Farke, CC BY 3.0), *Chaoyangsauridae* (Andrew A. Farke, CC BY 3.0), *Chasmosaurinae* (Jagged Fang Designs, public domain), *Dryosauridae* (Gereth Monger, CC BY 3.0), *Heterodontosauridae* (Scott Hartman, CC BY 3.0), *Iguanodontidae* (Tasman Dixon, public domain), *Lambeosaurinae* (Dmitry Bogdanov, CC BY 3.0), *Nodosaurinae* (Scott Hartman, public domain), *Polacanthinae* (FunkMonk, public domain), *Protoceratopsidae* (Andrew A. Farke, CC BY 3.0), *Rhabdodontidae* (Scott Hartman, CC BY 3.0), *Stegosauria* (Scott Hartman, CC BY 3.0). We have further added silhouettes for *Elasmaria* (Victoria M. Arbour, CC BY 4.0), *Pachycephalosauria* (Victoria M. Arbour, CC BY 4.0), *Saurolophinae* (Victoria M. Arbour, CC BY 4.0), and *Thescelosauridae* (Victoria M. Arbour, CC BY 4.0).

**Figure 2:**
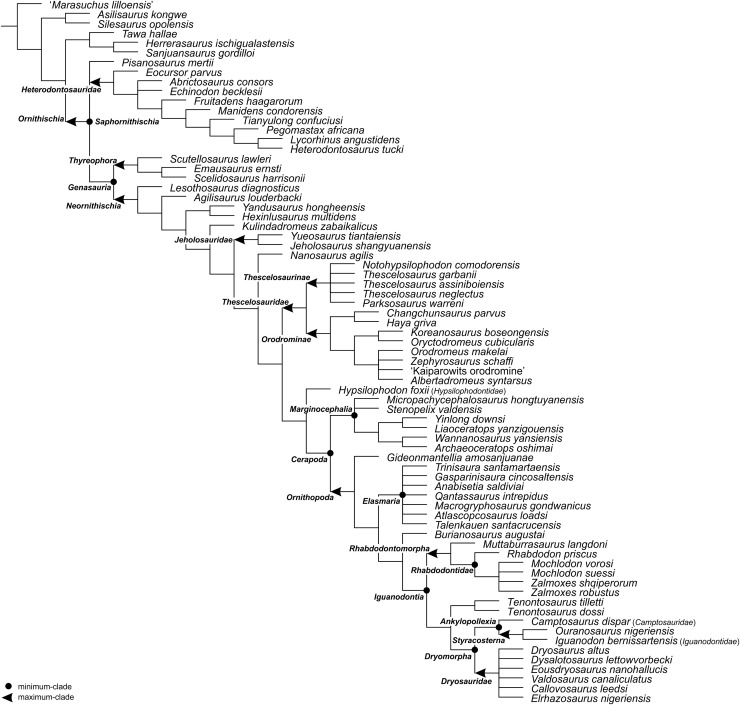
The phylogenetic nomenclature of ornithischian dinosaurs using the topology of Madzia, Boyd & Mazuch (2018: Fig. 4B). Note that *Nanosaurus agilis* has been analyzed by Madzia, Boyd & Mazuch (2018) as ‘*Othnielosaurus*’. The name was changed here following Carpenter & Galton (2018). Additionally, the name *Marasuchus lilloensis* was placed in quotation marks to highlight that the taxon may not be distinct from *Lagosuchus talampayensis* (Agnolin & Ezcurra, 2019).

**Figure 3:**
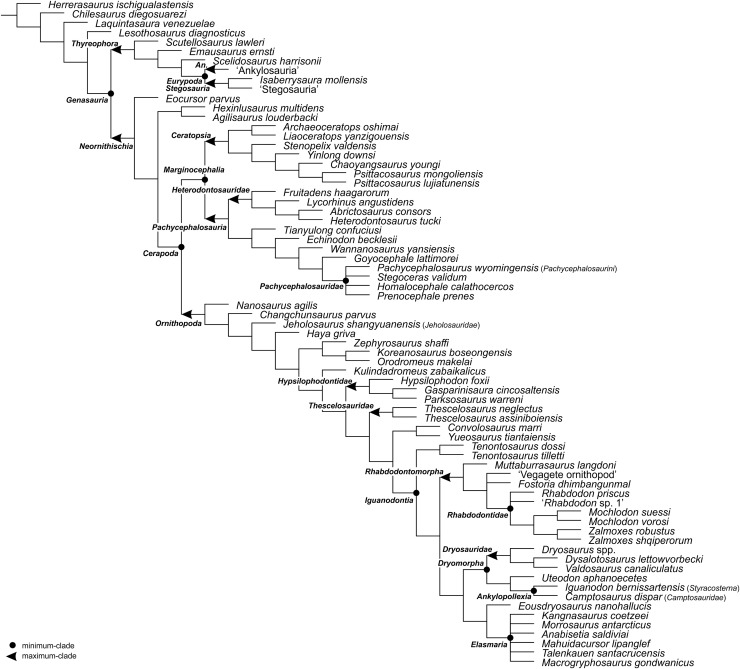
The phylogenetic nomenclature of ornithischian dinosaurs using the topology of Dieudonné et al. (2020: Figs. 1 and 2). Note that Dieudonné et al. (2020) followed Carpenter & Lamanna (2015) in placing *aphanoecetes* within *Camptosaurus*. Owing to the results of recent phylogenetic analyses (*e.g*., Madzia, Jagt & Mulder, 2020; Verdú et al., 2020), *aphanoecetes* is placed here within *Uteodon* McDonald, 2011. Additionally, the name *Psittacosaurus major* was changed to *Psittacosaurus lujiatunensis* (following Hedrick & Dodson, 2013), and *Ankylosauria* and *Stegosauria* of Dieudonné et al. (2020) were placed in quotation marks to highlight that these names have not been necessarily used by the authors as defined in the present study. Note also that the extent of *Ornithischia* is difficult to indicate on the tree because *Chilesaurus diegosuarezi* may represent a theropod (see ‘Discussion’). Abbreviation: *An*. – *Ankylosauria*.

**Figure 4:**
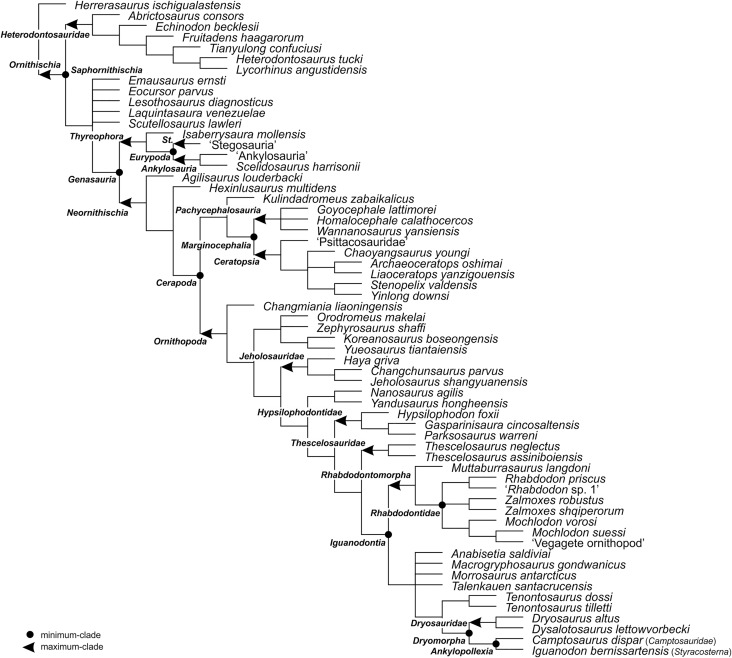
The phylogenetic nomenclature of ornithischian dinosaurs using the topology of Yang et al. (2020: Fig. 12). *Ankylosauria* and *Stegosauria* of Yang et al. (2020) were placed in quotation marks to highlight that these names have not been necessarily used by the authors as defined in the present study. In turn, *Psittacosauridae* of Yang et al. (2020) was placed in quotation marks because the name has not been formally defined yet. Abbreviation: *St*. – *Stegosauria*.

For the sake of clarity, all clade names are provided in alphabetical order. The definitions are summarized in Table 1. The extent of all clade names is further depicted on Fig. 1 that shows the relationships of taxa included in the present study as specifiers (both, internal as well as external) and additionally on Figs. 2–4 that represent selected ornithischian-wide phylogenies published within recent years: Madzia, Boyd & Mazuch (2018: Fig. 4B), Dieudonné et al. (2020: Figs. 1 and 2), and Yang et al. (2020: Fig. 12).

**Table 1:** The phylogenetic nomenclature of ornithischian dinosaurs.

Clade name	Authorship	Definition type	Abbreviated definition	Primary reference phylogeny
*Ankylopollexia*	Sereno, 1986	minimum-clade	min ∇ (*Camptosaurus dispar* (Marsh, 1879) & *Iguanodon bernissartensis* Boulenger in Beneden, 1881)	Figure 12 of Madzia, Jagt & Mulder (2020)
*Ankylosauria*	Osborn, 1923	maximum-clade	max ∇ (*Ankylosaurus magniventris* Brown, 1908 ~ *Stegosaurus stenops* Marsh, 1887)	Figure 11 of Arbour & Currie (2016)
*Ankylosauridae*	Brown, 1908	maximum-clade	max ∇ (*Ankylosaurus magniventris* Brown, 1908 ~ *Nodosaurus textilis* Marsh, 1889)	Figure 11 of Arbour & Currie (2016)
*Ankylosaurinae*	Nopcsa, 1918	maximum-clade	max ∇ (*Ankylosaurus magniventris* Brown, 1908 ~ *Shamosaurus scutatus* Tumanova, 1983)	Figure 11 of Arbour & Currie (2016)
*Ankylosaurini*	Arbour & Currie, 2016	maximum-clade	max ∇ (*Ankylosaurus magniventris* Brown, 1908 ~ *Pinacosaurus grangeri* Gilmore, 1933 & *Saichania chulsanensis* Maryańska, 1977)	Figure 11 of Arbour & Currie (2016)
*Aralosaurini*	Prieto-Márquez et al., 2013	maximum-clade	max ∇ (*Aralosaurus tuberiferus* Rozhdestvensky, 1968 & *Canardia garonnensis* Prieto-Márquez et al., 2013 ~ *Lambeosaurus lambei* Parks, 1923 & *Parasaurolophus walkeri* Parks, 1922 & *Tsintaosaurus spinorhinus* Young, 1958)	Figure 25 of Prieto-Márquez et al. (2013)
*Brachylophosaurini*	Gates et al., 2011	maximum-clade	max ∇ (*Brachylophosaurus canadensis* Sternberg, 1953 ~ *Edmontosaurus regalis* Lambe, 1917 & *Hadrosaurus foulkii* Leidy, 1858 & *Kritosaurus navajovius* Brown, 1910 & *Saurolophus osborni* Brown, 1912)	Figure 18 of Prieto-Márquez, Wagner & Lehman (2020)
*Camptosauridae*	Marsh, 1885	maximum-clade	max ∇ (*Camptosaurus dispar* (Marsh, 1879) ~ *Iguanodon bernissartensis* Boulenger in Beneden, 1881)	Figure 13 of Madzia, Jagt & Mulder (2020)
*Centrosaurinae*	Lambe, 1915	maximum-clade	max ∇ (*Centrosaurus apertus* Lambe, 1905 ~ *Chasmosaurus belli* (Lambe, 1902) & *Triceratops horridus* Marsh, 1889)	Figure 9 of Chiba et al. (2018)
*Centrosaurini*	Ryan et al., 2017	maximum-clade	max ∇ (*Centrosaurus apertus* Lambe, 1905 ~ *Pachyrhinosaurus canadensis* Sternberg, 1950)	Figure 9 of Chiba et al. (2018)
*Cerapoda*	Sereno, 1986	minimum-clade	min ∇ (*Iguanodon bernissartensis* Boulenger in Beneden, 1881 & *Pachycephalosaurus wyomingensis* (Gilmore, 1931) & *Triceratops horridus* Marsh, 1889)	Figure 4 of Madzia, Boyd & Mazuch (2018)
*Ceratopsia*	Marsh, 1890	maximum-clade	max ∇ (*Ceratops montanus* Marsh, 1888 & *Triceratops horridus* Marsh, 1889 ~ *Pachycephalosaurus wyomingensis* (Gilmore, 1931))	Figure 10 of Morschhauser et al. (2019)
*Ceratopsidae*	Marsh, 1888	minimum-clade	min ∇ (*Centrosaurus apertus* Lambe, 1905 & *Ceratops montanus* Marsh, 1888 & *Chasmosaurus belli* (Lambe, 1902) & *Triceratops horridus* Marsh, 1889)	Figure 4 of Yu et al. (2020)
*Ceratopsoidea*	Hay, 1902	maximum-clade	max ∇ (*Ceratops montanus* Marsh, 1888 & *Triceratops horridus* Marsh, 1889 ~ *Protoceratops andrewsi* Granger & Gregory, 1923)	Figure 4 of Yu et al. (2020)
*Chaoyangsauridae*	Zhao, Cheng & Xu, 1999	maximum-clade	max ∇ (*Chaoyangsaurus youngi* Zhao, Cheng & Xu, 1999 ~ *Psittacosaurus mongoliensis* Osborn, 1923 & *Triceratops horridus* Marsh, 1889)	Figure 10 of Morschhauser et al. (2019)
*Chasmosaurinae*	Lambe, 1915	maximum-clade	max ∇ (*Chasmosaurus belli* (Lambe, 1902) & *Triceratops horridus* Marsh, 1889 ~ *Centrosaurus apertus* Lambe, 1905)	Figure 9a of Fowler & Freedman Fowler (2020)
*Clypeodonta*	Norman, 2014	minimum-clade	min ∇ ∈ *Ornithopoda* (*Edmontosaurus regalis* Lambe, 1917 & *Hypsilophodon foxii* Huxley, 1869)	Figure 50 of Norman (2015)
*Coronosauria*	Sereno, 1986	minimum-clade	min ∇ (*Protoceratops andrewsi* Granger & Gregory, 1923 & *Triceratops horridus* Marsh, 1889)	Figure 10 of Morschhauser et al. (2019)
*Corythosauria*	New	minimum-clade	min ∇ (*Corythosaurus casuarius* Brown, 1914a & *Lambeosaurus lambei* Parks, 1923 & *Parasaurolophus walkeri* Parks, 1922)	Figure 18 of Prieto-Márquez, Wagner & Lehman (2020)
*Dryomorpha*	Sereno, 1986	minimum-clade	min ∇ (*Dryosaurus altus* (Marsh, 1878) & *Iguanodon bernissartensis* Boulenger in Beneden, 1881)	Figure 12 of Madzia, Jagt & Mulder (2020)
*Dryosauridae*	Milner & Norman, 1984	maximum-clade	max ∇ (*Dryosaurus altus* (Marsh, 1878) ~ *Iguanodon bernissartensis* Boulenger in Beneden, 1881)	Figure 12 of Madzia, Jagt & Mulder (2020)
*Edmontosaurini*	Glut, 1997	maximum-clade	max ∇ (*Edmontosaurus regalis* Lambe, 1917 ~ *Brachylophosaurus canadensis* Sternberg, 1953 & *Hadrosaurus foulkii* Leidy, 1858 & *Kritosaurus navajovius* Brown, 1910 & *Saurolophus osborni* Brown, 1912)	Figure 18 of Prieto-Márquez, Wagner & Lehman (2020)
*Elasmaria*	Calvo, Porfiri & Novas, 2007	minimum-clade	min ∇ (*Macrogryphosaurus gondwanicus* Calvo, Porfiri & Novas, 2007 & *Talenkauen santacrucensis* Novas, Cambiaso & Ambrosio, 2004 | ~ *Hypsilophodon foxii* Huxley, 1869 ∨ *Iguanodon bernissartensis* Boulenger in Beneden, 1881 ∨ *Thescelosaurus neglectus* Gilmore, 1913)	Figure 31 of Rozadilla, Agnolín & Novas (2019)
*Eucentrosaura*	Chiba et al., 2018	minimum-clade	min ∇ (*Centrosaurus apertus* Lambe, 1905 & *Pachyrhinosaurus canadensis* Sternberg, 1950)	Figure 9 of Chiba et al. (2018)
*Euceratopsia*	New	minimum-clade	min ∇ (*Leptoceratops gracilis* Brown, 1914b & *Protoceratops andrewsi* Granger & Gregory, 1923 & *Triceratops horridus* Marsh, 1889)	Figure 4 of Yu et al. (2020)
*Euhadrosauria*	Weishampel, Norman & Grigorescu, 1993	minimum-clade	min ∇ (*Lambeosaurus lambei* Parks, 1923 & *Saurolophus osborni* Brown, 1912 | ~ *Hadrosaurus foulkii* Leidy, 1858)	Figure 18 of Prieto-Márquez, Wagner & Lehman (2020)
*Euiguanodontia*	Coria & Salgado, 1996	minimum-clade	min ∇ (*Camptosaurus dispar* (Marsh, 1879) & *Dryosaurus altus* (Marsh, 1878) & *Gasparinisaura cincosaltensis* Coria & Salgado, 1996 | ~ *Tenontosaurus tilletti* Ostrom, 1970)	Figure 13 of Coria & Salgado (1996)
*Euornithopoda*	Sereno, 1986	maximum-clade	max ∇ ∈ *Ornithopoda* (*Iguanodon bernissartensis* Boulenger in Beneden, 1881 ~ *Heterodontosaurus tucki* Crompton & Charig, 1962)	Figure 1 of Sereno (1999)
*Eurypoda*	Sereno, 1986	minimum-clade	min ∇ (*Ankylosaurus magniventris* Brown, 1908 & *Stegosaurus stenops* Marsh, 1887)	Figure 3 of Thompson et al. (2012)
*Genasauria*	Sereno, 1986	minimum-clade	min ∇ (*Ankylosaurus magniventris* Brown, 1908 & *Iguanodon bernissartensis* Boulenger in Beneden, 1881 & *Stegosaurus stenops* Marsh, 1887 & *Triceratops horridus* Marsh, 1889)	Figure 16 of Han et al. (2018)
*Hadrosauridae*	Cope, 1869	minimum-clade	min ∇ (*Hadrosaurus foulkii* Leidy, 1858 & *Lambeosaurus lambei* Parks, 1923 & *Saurolophus osborni* Brown, 1912)	Figure 18 of Prieto-Márquez, Wagner & Lehman (2020)
*Hadrosauriformes*	Sereno, 1997	minimum-clade	min ∇ (*Hadrosaurus foulkii* Leidy, 1858 & *Iguanodon bernissartensis* Boulenger in Beneden, 1881)	Figure 12 of Madzia, Jagt & Mulder (2020)
*Hadrosaurinae*	Lambe, 1918	maximum-clade	max ∇ (*Hadrosaurus foulkii* Leidy, 1858 ~ *Lambeosaurus lambei* Parks, 1923)	Figure 5 of Kobayashi et al. (2019)
*Hadrosauroidea*	von Huene, 1952	maximum-clade	max ∇ (*Hadrosaurus foulkii* Leidy, 1858 ~ *Iguanodon bernissartensis* Boulenger in Beneden, 1881)	Figure 12 of Madzia, Jagt & Mulder (2020)
*Hadrosauromorpha*	Norman, 2014	maximum-clade	max ∇ (*Hadrosaurus foulkii* Leidy, 1858 ~ *Probactrosaurus gobiensis* Rozhdestvensky, 1966)	Figure 12 of Madzia, Jagt & Mulder (2020)
*Heterodontosauridae*	Kuhn, 1966	maximum-clade	max ∇ (*Heterodontosaurus tucki* Crompton & Charig, 1962 ~ *Iguanodon bernissartensis* Boulenger in Beneden, 1881 & *Pachycephalosaurus wyomingensis* (Gilmore, 1931) & *Stegosaurus stenops* Marsh, 1887 & *Triceratops horridus* Marsh, 1889)	Figure 4 of Madzia, Boyd & Mazuch (2018)
*Huayangosauridae*	Dong, Tang & Zhou, 1982	maximum-clade	max ∇ (*Huayangosaurus taibaii* Dong, Tang & Zhou, 1982 ~ *Stegosaurus stenops* Marsh, 1887)	Figure 12 of Maidment et al. (2020)
*Hypsilophodontia*	Cooper, 1985	minimum-clade	min ∇ ∈ *Ornithopoda* (*Hypsilophodon foxii* Huxley, 1869 & *Tenontosaurus tilletti* Ostrom, 1970 | ~ *Iguanodon bernissartensis* Boulenger in Beneden, 1881)	Figure 50 of Norman (2015)
*Hypsilophodontidae*	Dollo, 1882	maximum-clade	max ∇ (*Hypsilophodon foxii* Huxley, 1869 ~ *Iguanodon bernissartensis* Boulenger in Beneden, 1881 & *Rhabdodon priscus* Matheron, 1869)	Figure 2 of Dieudonné et al. (2020)
*Iguanodontia*	Baur, 1891	minimum-clade	min ∇ (*Dryosaurus altus* (Marsh, 1878) & *Iguanodon bernissartensis* Boulenger in Beneden, 1881 & *Rhabdodon priscus* Matheron, 1869 & *Tenontosaurus tilletti* Ostrom, 1970 | ~ *Hypsilophodon foxii* Huxley, 1869)	Figure 12 of Madzia, Jagt & Mulder (2020)
*Iguanodontidae*	Bonaparte, 1850	maximum-clade	max ∇ (*Iguanodon bernissartensis* Boulenger in Beneden, 1881 ~ *Hadrosaurus foulkii* Leidy, 1858)	Figure 13 of Madzia, Jagt & Mulder (2020)
*Jeholosauridae*	Han et al., 2012	maximum-clade	max ∇ ∉ *Hypsilophodontidae* ∨ *Thescelosauridae* (*Jeholosaurus shangyuanensis* Xu, Wang & You, 2000 ~ *Hypsilophodon foxii* Huxley, 1869 & *Iguanodon bernissartensis* Boulenger in Beneden, 1881 & *Pachycephalosaurus wyomingensis* (Gilmore, 1931) & *Thescelosaurus neglectus* Gilmore, 1913 & *Triceratops horridus* Marsh, 1889)	Figure 25 of Herne et al. (2019)
*Kritosaurini*	Glut, 1997	maximum-clade	max ∇ (*Kritosaurus navajovius* Brown, 1910 ~ *Brachylophosaurus canadensis* Sternberg, 1953 & *Edmontosaurus regalis* Lambe, 1917 & *Hadrosaurus foulkii* Leidy, 1858 & *Saurolophus osborni* Brown, 1912)	Figure 18 of Prieto-Márquez, Wagner & Lehman (2020)
*Lambeosaurinae*	Parks, 1923	maximum-clade	max ∇ (*Lambeosaurus lambei* Parks, 1923 ~ *Hadrosaurus foulkii* Leidy, 1858 & *Saurolophus osborni* Brown, 1912)	Figure 18 of Prieto-Márquez, Wagner & Lehman (2020)
*Lambeosaurini*	Sullivan et al., 2011	maximum-clade	max ∇ (*Lambeosaurus lambei* Parks, 1923 ~ *Aralosaurus tuberiferus* Rozhdestvensky, 1968 & *Parasaurolophus walkeri* Parks, 1922 & *Tsintaosaurus spinorhinus* Young, 1958)	Figure 18 of Prieto-Márquez, Wagner & Lehman (2020)
*Leptoceratopsidae*	Nopcsa, 1923	maximum-clade	max ∇ (*Leptoceratops gracilis* Brown, 1914b ~ *Protoceratops andrewsi* Granger & Gregory, 1923 & *Triceratops horridus* Marsh, 1889)	Figure 10 of Morschhauser et al. (2019)
*Marginocephalia*	Sereno, 1986	minimum-clade	min ∇ (*Ceratops montanus* Marsh, 1888 & *Pachycephalosaurus wyomingensis* (Gilmore, 1931) & *Triceratops horridus* Marsh, 1889)	Figure 16 of Han et al. (2018)
*Nasutoceratopsini*	Ryan et al., 2017	maximum-clade	max ∇ (*Nasutoceratops titusi* Sampson et al., 2013 ~ *Centrosaurus apertus* Lambe, 1905)	Figure 9 of Chiba et al. (2018)
*Neoceratopsia*	Sereno, 1986	maximum-clade	max ∇ (*Triceratops horridus* Marsh, 1889 ~ *Chaoyangsaurus youngi* Zhao, Cheng & Xu, 1999 & *Psittacosaurus mongoliensis* Osborn, 1923)	Figure 10 of Morschhauser et al. (2019)
*Neoiguanodontia*	Norman, 2014	minimum-clade	min ∇ (*Hypselospinus fittoni* (Lydekker, 1889) & *Iguanodon bernissartensis* Boulenger in Beneden, 1881 & *Parasaurolophus walkeri* Parks, 1922)	Figure 2.26 of Norman (2014)
*Neornithischia*	Cooper, 1985	maximum-clade	max ∇ (*Iguanodon bernissartensis* Boulenger in Beneden, 1881 & *Triceratops horridus* Marsh, 1889 ~ *Ankylosaurus magniventris* Brown, 1908 & *Stegosaurus stenops* Marsh, 1887)	Figure 4 of Madzia, Boyd & Mazuch (2018)
*Nodosauridae*	Marsh, 1890	maximum-clade	max ∇ (*Nodosaurus textilis* Marsh, 1889 ~ *Ankylosaurus magniventris* Brown, 1908)	Figure 5 of Rivera-Sylva et al. (2018a)
*Nodosaurinae*	Abel, 1919	maximum-clade	max ∇ (*Nodosaurus textilis* Marsh, 1889 ~ *Hylaeosaurus armatus* Mantell, 1833 & *Mymoorapelta maysi* Kirkland & Carpenter, 1994 & *Polacanthus foxii* Owen in Anonymous, 1865)	Figure 5 of Rivera-Sylva et al. (2018a)
*Ornithischia*	Seeley, 1888	maximum-clade	max ∇ (*Iguanodon bernissartensis* Boulenger in Beneden, 1881 ~ *Allosaurus fragilis* Marsh, 1877a & *Camarasaurus supremus* Cope, 1877)	Figure 4 of Madzia, Boyd & Mazuch (2018)
*Ornithopoda*	Marsh, 1881	maximum-clade	max ∇ (*Iguanodon bernissartensis* Boulenger in Beneden, 1881 ~ *Pachycephalosaurus wyomingensis* (Gilmore, 1931) & *Triceratops horridus* Marsh, 1889)	Figure 4 of Madzia, Boyd & Mazuch (2018)
*Orodrominae*	Brown et al., 2013	maximum-clade	max ∇ ∈ *Hypsilophodontidae* ∨ *Thescelosauridae* (*Orodromeus makelai* Horner & Weishampel, 1988 ~ *Hypsilophodon foxii* Huxley, 1869 & *Thescelosaurus neglectus* Gilmore, 1913)	Figure 4 of Madzia, Boyd & Mazuch (2018)
*Pachycephalosauria*	Maryańska & Osmólska, 1974	maximum-clade	max ∇ (*Pachycephalosaurus wyomingensis* (Gilmore, 1931) ~ *Ceratops montanus* Marsh, 1888 & *Triceratops horridus* Marsh, 1889)	Figure 27 of Schott & Evans (2017)
*Pachycephalosauridae*	Sternberg, 1945	minimum-clade	min ∇ (*Pachycephalosaurus wyomingensis* (Gilmore, 1931) & *Stegoceras validum* Lambe, 1902 | ~ *Heterodontosaurus tucki* Crompton & Charig, 1962)	Figure 27 of Schott & Evans (2017)
*Pachycephalosaurinae*	Sereno, 1997	maximum-clade	max ∇ (*Pachycephalosaurus wyomingensis* (Gilmore, 1931) ~ *Stegoceras validum* Lambe, 1902)	Figure 27 of Schott & Evans (2017)
*Pachycephalosaurini*	Sullivan, 2003	maximum-clade	max ∇ (*Pachycephalosaurus wyomingensis* (Gilmore, 1931) ~ *Prenocephale prenes* Maryańska & Osmólska, 1974 & *Sphaerotholus goodwini* Williamson & Carr, 2003)	Figure 27 of Schott & Evans (2017)
*Pachyrhinosaurini*	Fiorillo & Tykoski, 2012	maximum-clade	max ∇ (*Pachyrhinosaurus canadensis* Sternberg, 1950 ~ *Centrosaurus apertus* Lambe, 1905)	Figure 9 of Chiba et al. (2018)
*Pachyrostra*	Fiorillo & Tykoski, 2012	minimum-clade	min ∇ (*Achelousaurus horneri* Sampson, 1995 & *Pachyrhinosaurus canadensis* Sternberg, 1950)	Figure 9 of Chiba et al. (2018)
*Panoplosaurini*	New	maximum-clade	max ∇ (*Panoplosaurus mirus* Lambe, 1919 ~ *Nodosaurus textilis* Marsh, 1889 & *Struthiosaurus austriacus* Bunzel, 1871)	Figure 5 of Rivera-Sylva et al. (2018a)
*Parasaurolophini*	Glut, 1997	maximum-clade	max ∇ (*Parasaurolophus walkeri* Parks, 1922 ~ *Aralosaurus tuberiferus* Rozhdestvensky, 1968 & *Lambeosaurus lambei* Parks, 1923 & *Tsintaosaurus spinorhinus* Young, 1958)	Figure 18 of Prieto-Márquez, Wagner & Lehman (2020)
*Polacanthinae*	Lapparent & Lavocat, 1955	maximum-clade	max ∇ ∈ *Ankylosauridae* ∨ *Nodosauridae* (*Polacanthus foxii* Owen in Anonymous, 1865 ~ *Ankylosaurus magniventris* Brown, 1908 & *Nodosaurus textilis* Marsh, 1889)	Figure 9 of Yang et al. (2013)
*Protoceratopsidae*	Granger & Gregory, 1923	maximum-clade	max ∇ (*Protoceratops andrewsi* Granger & Gregory, 1923 ~ *Ceratops montanus* Marsh, 1888 & *Leptoceratops gracilis* Brown, 1914b & *Triceratops horridus* Marsh, 1889)	Figure 10 of Morschhauser et al. (2019)
*Rhabdodontidae*	Weishampel et al., 2003	minimum-clade	min ∇ (*Rhabdodon priscus* Matheron, 1869 & *Zalmoxes robustus* (Nopcsa, 1900))	Figure 4 of Madzia, Boyd & Mazuch (2018)
*Rhabdodontomorpha*	Dieudonné et al., 2016	maximum-clade	max ∇ (*Rhabdodon priscus* Matheron, 1869 ~ *Hypsilophodon foxii* Huxley, 1869 & *Iguanodon bernissartensis* Boulenger in Beneden, 1881)	Figure 2 of Dieudonné et al. (2020)
*Saphornithischia*	New	minimum-clade	min ∇ (*Heterodontosaurus tucki* Crompton & Charig, 1962 & *Iguanodon bernissartensis* Boulenger in Beneden, 1881 & *Stegosaurus stenops* Marsh, 1887 & *Triceratops horridus* Marsh, 1889)	Figure 4 of Madzia, Boyd & Mazuch (2018)
*Saurolophinae*	Brown, 1914a	maximum-clade	max ∇ (*Saurolophus osborni* Brown, 1912 ~ *Lambeosaurus lambei* Parks, 1923 | ~ *Hadrosaurus foulkii* Leidy, 1858)	Figure 18 of Prieto-Márquez, Wagner & Lehman (2020)
*Saurolophini*	Glut, 1997	maximum-clade	max ∇ (*Saurolophus osborni* Brown, 1912 ~ *Brachylophosaurus canadensis* Sternberg, 1953 & *Edmontosaurus regalis* Lambe, 1917 & *Hadrosaurus foulkii* Leidy, 1858 & *Kritosaurus navajovius* Brown, 1910)	Figure 18 of Prieto-Márquez, Wagner & Lehman (2020)
*Shamosaurinae*	Tumanova, 1983	maximum-clade	max ∇ (*Gobisaurus domoculus* Vickaryous et al., 2001 & *Shamosaurus scutatus* Tumanova, 1983 ~ *Ankylosaurus magniventris* Brown, 1908)	Figure 11 of Arbour & Currie (2016)
*Stegosauria*	Marsh, 1877b	maximum-clade	max ∇ (*Stegosaurus stenops* Marsh, 1887 ~ *Ankylosaurus magniventris* Brown, 1908)	Figure 12 of Maidment et al. (2020)
*Stegosauridae*	Marsh, 1880	maximum-clade	max ∇ (*Stegosaurus stenops* Marsh, 1887 ~ *Huayangosaurus taibaii* Dong, Tang & Zhou, 1982)	Figure 12 of Maidment et al. (2020)
*Struthiosaurini*	New	maximum-clade	max ∇ (*Struthiosaurus austriacus* Bunzel, 1871 ~ *Nodosaurus textilis* Marsh, 1889 & *Panoplosaurus mirus* Lambe, 1919)	Figure 5 of Rivera-Sylva et al. (2018a)
*Styracosterna*	Sereno, 1986	maximum-clade	max ∇ (*Iguanodon bernissartensis* Boulenger in Beneden, 1881 ~ *Camptosaurus dispar* (Marsh, 1879))	Figure 12 of Madzia, Jagt & Mulder (2020)
*Thescelosauridae*	Sternberg, 1937	maximum-clade	max ∇ (*Thescelosaurus neglectus* Gilmore, 1913 ~ *Iguanodon bernissartensis* Boulenger in Beneden, 1881 | ~ *Hypsilophodon foxii* Huxley, 1869)	Figure 4 of Madzia, Boyd & Mazuch (2018)
*Thescelosaurinae*	Sternberg, 1940	maximum-clade	max ∇ ∈ *Hypsilophodontidae* ∨ *Thescelosauridae* (*Thescelosaurus neglectus* Gilmore, 1913 ~ *Hypsilophodon foxii* Huxley, 1869 & *Orodromeus makelai* Horner & Weishampel, 1988)	Figure 4 of Madzia, Boyd & Mazuch (2018)
*Thyreophora*	Nopcsa, 1915	maximum-clade	max ∇ (*Ankylosaurus magniventris* Brown, 1908 & *Stegosaurus stenops* Marsh, 1887 ~ *Iguanodon bernissartensis* Boulenger in Beneden, 1881 & *Triceratops horridus* Marsh, 1889)	Figure 16 of Han et al. (2018)
*Triceratopsini*	Longrich, 2011	maximum-clade	max ∇ (*Triceratops horridus* Marsh, 1889 ~ *Anchiceratops ornatus* Brown, 1914c & *Arrhinoceratops brachyops* Parks, 1925)	Figure 9a of Fowler & Freedman Fowler (2020)
*Tsintaosaurini*	Prieto-Márquez et al., 2013	maximum-clade	max ∇ (*Pararhabdodon isonensis* Casanovas-Cladellas, Santafé-Llopis & Isidro-Llorens, 1993 & *Tsintaosaurus spinorhinus* Young, 1958 ~ *Aralosaurus tuberiferus* Rozhdestvensky, 1968 & *Lambeosaurus lambei* Parks, 1923 & *Parasaurolophus walkeri* Parks, 1922)	Figure 18 of Prieto-Márquez, Wagner & Lehman (2020)

### *Ankylopollexia* Sereno, 1986 (converted clade name)

**Registration number:** 585

**Definition.** The smallest clade containing *Camptosaurus dispar* (Marsh, 1879) and *Iguanodon bernissartensis* Boulenger in Beneden, 1881. This is a minimum-clade definition. Abbreviated definition: min ∇ (*Camptosaurus dispar* (Marsh, 1879) & *Iguanodon bernissartensis* Boulenger in Beneden, 1881).

**Reference phylogeny.** Figure 12 of Madzia, Jagt & Mulder (2020) is treated here as the primary reference phylogeny. Additional reference phylogenies include Figure 3 of Madzia, Boyd & Mazuch (2018), Figure 20 of Verdú et al. (2018), Figure 9 of Verdú et al. (2020), Figure 11 of McDonald et al. (2021), and Figure 11 of Santos-Cubedo et al. (2021).

**Composition.** The clade *Ankylopollexia* comprises *Camptosaurus dispar* and members of the clade *Styracosterna*.

**Synonyms.** No other taxon names are currently in use for the same or approximate clade.

**Comments.** The name *Ankylopollexia* has been (informally) defined before by Sereno (1998: 62) who applied the minimum-clade definition and used *Camptosaurus* and *Parasaurolophus* as the internal specifiers. Since the name has traditionally been used in the exact sense, we apply it to the same clade, but prefer to use *Iguanodon bernissartensis* as the second internal specifier rather than *P. walkeri* because the name *Ankylopollexia* was formed after the stiff cone-shaped thumb that characterizes *Iguanodon*-grade ornithopods. The inclusion of a different internal specifier does not change the extent of *Ankylopollexia* under any of the published phylogeny inferences. Also, even though the name derives from an apomorphy, it was never used for an apomorphy-based clade.

### *Ankylosauria* Osborn, 1923 (converted clade name)

**Registration number:** 588

**Definition.** The largest clade containing *Ankylosaurus magniventris* Brown, 1908 but not *Stegosaurus stenops* Marsh, 1887. This is a maximum-clade definition. Abbreviated definition: max ∇ (*Ankylosaurus magniventris* Brown, 1908 ~ *Stegosaurus stenops* Marsh, 1887).

**Reference phylogeny.** Figure 11 of Arbour & Currie (2016) is treated here as the primary reference phylogeny. Additional reference phylogenies include Figure 3 of Thompson et al. (2012), Figure 1 of Arbour, Zanno & Gates (2016), Figure 3 of Brown et al. (2017), and Figure 26 of Wiersma & Irmis (2018).

**Composition.** Under the primary reference phylogeny, *Ankylosauria* comprises *Minmi* sp. (= *Kunbarrasaurus ieversi*), *Mymoorapelta maysi*, and members of the clades *Ankylosauridae* and *Nodosauridae*.

**Synonyms.** The name *Ankylosauromorpha* Carpenter, 2001 has been recently used under an alternative systematic scheme for the same branch as *Ankylosauria*, as defined herein (Norman, 2021; see ‘Discussion’). No other taxon names are currently in use for the same or approximate clade.

**Comments.** The name *Ankylosauria* has been (informally) defined before (Carpenter, 1997; Sereno, 1998; Sereno, 2005). These definitions were maximum-clade and used *Ankylosaurus* (Carpenter, 1997; Sereno, 1998) or *Ankylosaurus magniventris* (Sereno, 2005) as the internal specifier and *Stegosaurus* (Carpenter, 1997; Sereno, 1998) or *Stegosaurus stenops* (Sereno, 2005) as the external specifier. Since *Ankylosauria* has been ‘traditionally’ used in this sense (though, see also ‘Discussion’), we formalize this definition. Note that Norman (2021) recently provided two phylogenetic definitions for *Ankylosauria*, a maximum-clade and a minimum-clade. In the maximum-clade definition Norman (2021) used *Euoplocephalus* and *Edmontonia* as the internal specifiers and *Scelidosaurus* as the external specifier, while in the minimum-clade definition the use of the name was anchored on *Euoplocephalus* and *Edmontonia*. See ‘Discussion’ for additional comments. Note that the external specifier *Stegosaurus stenops* is not included in the primary reference phylogeny. From the taxa analyzed by Arbour & Currie (2016), *S. stenops* is most closely related to *Huayangosaurus taibaii* (see, *e.g*., Maidment et al., 2020).

### *Ankylosauridae* Brown, 1908 (converted clade name)

**Registration number:** 589

**Definition.** The largest clade containing *Ankylosaurus magniventris* Brown, 1908 but not *Nodosaurus textilis* Marsh, 1889. This is a maximum-clade definition. Abbreviated definition: max ∇ (*Ankylosaurus magniventris* Brown, 1908 ~ *Nodosaurus textilis* Marsh, 1889).

**Reference phylogeny.** Figure 11 of Arbour & Currie (2016) is treated here as the primary reference phylogeny. Additional reference phylogenies include Figure 3 of Thompson et al. (2012), Figure 1 of Arbour, Zanno & Gates (2016), Figure 3 of Brown et al. (2017), Figure 26 of Wiersma & Irmis (2018), and Figure 9 of Zheng et al. (2018).

**Composition.** Under the primary reference phylogeny, *Ankylosauridae* comprises *Ahshislepelta minor*, *Aletopelta coombsi*, *Cedarpelta bilbeyhallorum*, *Chuanqilong chaoyangensis*, *Gastonia burgei*, *Liaoningosaurus paradoxus*, and members of the clades *Shamosaurinae* and *Ankylosaurinae*.

**Synonyms.** No other taxon names are currently in use for the same or approximate clade.

**Comments.** The name *Ankylosauridae* has been (informally) defined before by Sereno (1998, 2005) who applied a maximum-clade definition and used *Ankylosaurus magniventris* as the internal specifier and *Panoplosaurus mirus* as the external specifier. Considering that *Ankylosauridae* has been traditionally used as a sister taxon to *Nodosauridae* (see, *e.g*., Thompson et al., 2012 for details), we use a definition that incorporates *Nodosaurus textilis* as the external specifier. Note that *N. textilis* is not included in the primary reference phylogeny. Both, *A. magniventris* and *N. textilis* were analyzed by, and their relationship is indicated in, Rivera-Sylva et al. (2018a).

### *Ankylosaurinae* Nopcsa, 1918 (converted clade name)

**Registration number:** 590

**Definition.** The largest clade containing *Ankylosaurus magniventris* Brown, 1908 but not *Shamosaurus scutatus* Tumanova, 1983. This is a maximum-clade definition. Abbreviated definition: max ∇ (*Ankylosaurus magniventris* Brown, 1908 ~ *Shamosaurus scutatus* Tumanova, 1983).

**Reference phylogeny.** Figure 11 of Arbour & Currie (2016) is treated here as the primary reference phylogeny. Additional reference phylogenies include Figure 3 of Thompson et al. (2012), Figure 1 of Arbour, Zanno & Gates (2016), Figure 8 of Arbour & Evans (2017), Figure 26 of Wiersma & Irmis (2018), and Figure 9 of Zheng et al. (2018).

**Composition.** Under the primary reference phylogeny, *Ankylosaurinae* comprises *Crichtonpelta benxiensis*, *Pinacosaurus* spp., *Saichania chulsanensis*, *Tarchia kielanae*, *Tsagantegia longicranialis*, *Zaraapelta nomadis*, ‘*Zhejiangosaurus luoyangensis*’, and members of the clade *Ankylosaurini*.

**Synonyms.** No other taxon names are currently in use for the same or approximate clade.

**Comments.** The name *Ankylosaurinae* was (informally) defined before (Sereno, 1998; Sereno, 2005; Vickaryous, Maryanska & Weishampel, 2004). All these definitions were maximum-clade and used *Ankylosaurus* (Sereno, 1998) or *Ankylosaurus magniventris* (Sereno, 2005; Vickaryous, Maryanska & Weishampel, 2004) as the internal specifiers and *Minmi paravertebra* and *Shamosaurus scutatus* (Sereno, 1998), *Gargoyleosaurus parkpinorum*, *Minmi paravertebra*, and *Shamosaurus scutatus* (Sereno, 2005) or only *Shamosaurus scutatus* (Vickaryous, Maryanska & Weishampel, 2004) as the external specifiers. Owing to the dubious taxonomic status of ‘*M. paravertebra*’ (Arbour & Currie, 2016) and non-ankylosaurid affinities of *G. parkpinorum* (*e.g*., Arbour & Currie, 2016; Rivera-Sylva et al., 2018a; Wiersma & Irmis, 2018; Zheng et al., 2018), we formalize the definition of Vickaryous, Maryanska & Weishampel (2004) in that we use a single external specifier (*Shamosaurus scutatus*).

### *Ankylosaurini* Arbour & Currie, 2016 (converted clade name)

**Registration number:** 592

**Definition.** The largest clade containing *Ankylosaurus magniventris* Brown, 1908 but not *Pinacosaurus grangeri* Gilmore, 1933 and *Saichania chulsanensis* Maryańska, 1977. This is a maximum-clade definition. Abbreviated definition: max ∇ (*Ankylosaurus magniventris* Brown, 1908 ~ *Pinacosaurus grangeri* Gilmore, 1933 & *Saichania chulsanensis* Maryańska, 1977).

**Reference phylogeny.** Figure 11 of Arbour & Currie (2016) is treated here as the primary reference phylogeny. Additional reference phylogenies include Figure 1 of Arbour, Zanno & Gates (2016), Figure 8 of Arbour & Evans (2017), Figure 26 of Wiersma & Irmis (2018), and Figure 9 of Zheng et al. (2018).

**Composition.** Under the primary reference phylogeny, *Ankylosaurini* comprises *Ankylosaurus magniventris*, *Anodontosaurus lambei*, *Dyoplosaurus acutosquameus*, *Euoplocephalus tutus*, *Nodocephalosaurus kirtlandensis*, *Scolosaurus cutleri*, *Talarurus plicatospineus*, and *Ziapelta sanjuanensis*.

**Synonyms.** No other taxon names are currently in use for the same or approximate clade.

**Comments.** The name *Ankylosaurini* was first (informally) defined by Arbour & Currie (2016) who applied the maximum-clade definition and used *Ankylosaurus magniventris* as the internal specifier and *Pinacosaurus grangeri* and *Saichania chulsanensis* as the external specifiers. The name was used for a clade that largely includes later-diverging North American ankylosaurines, many of which were previously synonymized with *Euoplocephalus tutus* (Arbour & Currie, 2013), although under some topologies the name may be more restricted in its use (Thompson et al., 2012).

### *Aralosaurini* Prieto-Márquez et al., 2013 (converted clade name)

**Registration number:** 593

**Definition.** The largest clade containing *Aralosaurus tuberiferus* Rozhdestvensky, 1968 and *Canardia garonnensis* Prieto-Márquez et al., 2013 but not *Lambeosaurus lambei* Parks, 1923, *Parasaurolophus walkeri* Parks, 1922, and *Tsintaosaurus spinorhinus* Young, 1958. This is a maximum-clade definition. Abbreviated definition: max ∇ (*Aralosaurus tuberiferus* Rozhdestvensky, 1968 & *Canardia garonnensis* Prieto-Márquez et al., 2013 ~ *Lambeosaurus lambei* Parks, 1923 & *Parasaurolophus walkeri* Parks, 1922 & *Tsintaosaurus spinorhinus* Young, 1958).

**Reference phylogeny.** Figure 25 of Prieto-Márquez et al. (2013) is treated here as the primary reference phylogeny. Additional reference phylogeny includes Figure 11 of McDonald et al. (2021).

**Composition.** Under the primary reference phylogeny, *Aralosaurini* comprises *Aralosaurus tuberiferus* and *Canardia garonnensis*.

**Synonyms.** No other taxon names are currently in use for the same or approximate clade.

**Comments.** The name was first (informally) defined by Prieto-Márquez et al. (2013) who applied the minimum-clade definition and used *Aralosaurus tuberiferus* and *Canardia garonnensis* as the internal specifiers. Following such definition, however, *Aralosaurini* would cover the entire lambeosaurine branch under some topologies that include both of the internal specifiers (Kobayashi et al., 2019; Prieto-Márquez et al., 2019; Zhang et al., 2019; Gates, Evans & Sertich, 2021; Kobayashi et al., 2021; Longrich et al., 2021), or would even comprise the same contents as *Euhadrosauria* (Ramírez-Velasco et al., 2021). Recently, however, McDonald et al. (2021) inferred *Aralosaurini* as delimited by Prieto-Márquez et al. (2013). Therefore, we define the name but make it inapplicable under a subset of recent phylogenies.

### *Brachylophosaurini* Gates et al., 2011 (converted clade name)

**Registration number:** 594

**Definition.** The largest clade containing *Brachylophosaurus canadensis* Sternberg, 1953 but not *Edmontosaurus regalis* Lambe, 1917, *Hadrosaurus foulkii* Leidy, 1858, *Kritosaurus navajovius* Brown, 1910, and *Saurolophus osborni* Brown, 1912. This is a maximum-clade definition. Abbreviated definition: max ∇ (*Brachylophosaurus canadensis* Sternberg, 1953 ~ *Edmontosaurus regalis* Lambe, 1917 & *Hadrosaurus foulkii* Leidy, 1858 & *Kritosaurus navajovius* Brown, 1910 & *Saurolophus osborni* Brown, 1912).

**Reference phylogeny.** Figure 18 of Prieto-Márquez, Wagner & Lehman (2020) is treated here as the primary reference phylogeny. Additional reference phylogenies include Figure 5 of Kobayashi et al. (2019), Figure 11 of Prieto-Márquez et al. (2019), Figure 9 of Zhang et al. (2019), Figure 5 of Zhang et al. (2020), Figure 7 of Kobayashi et al. (2021), and Figure 10 of Longrich et al. (2021).

**Composition.** Under the primary reference phylogeny, *Brachylophosaurini* comprises *Acristavus gagslarsoni*, *Brachylophosaurus canadensis*, *Maiasaura peeblesorum*, and *Probrachylophosaurus bergei* (erroneously named ‘*Probrachylophosaurus canadensis*’ in the primary reference phylogeny).

**Synonyms.** The name *Maiasaurini* Sereno, 2005 is an approximate synonym of *Brachylophosaurini*. To our knowledge, the name was used only in two recent papers (McFeeters et al., 2021; McFeeters, Evans & Maddin, 2021) that attributed the name to Horner (1992). However, this attribution was due to the adherence of the authors to the Principle of Coordination, as Horner (1992) used the name *Maiasaurinae*. Nevertheless, all recent phylogenetic studies consistently use *Brachylophosaurini* (*e.g*., Freedman Fowler & Horner, 2015; Cruzado-Caballero & Powell, 2017; Xing, Mallon & Currie, 2017; Kobayashi et al., 2019; Zhang et al., 2019; Prieto-Márquez, Wagner & Lehman, 2020; Zhang et al., 2020; Kobayashi et al., 2021; McDonald et al., 2021). No other taxon names are currently in use for the same or approximate clade.

**Comments.** The name *Brachylophosaurini* has been (informally) defined before (Gates et al., 2011; Freedman Fowler & Horner, 2015). These definitions were maximum-clade and used *Brachylophosaurus*, *Maiasaura*, and *Acristavus* (Gates et al., 2011) or *Brachylophosaurus*, *Probrachylophosaurus*, *Maiasaura*, and *Acristavus* (Freedman Fowler & Horner, 2015) as the internal specifiers and *Gryposaurus* and *Saurolophus* as the external specifiers. The composition of *Brachylophosaurini* and the relationships of the clade to other hadrosaurids have been stable across studies since the introduction of the name. Therefore, using more than one internal specifier is unnecessary. We use a definition that ensures *Brachylophosaurini* does not cover taxa ‘traditionally’ comprised within *Edmontosaurini*, *Kritosaurini*, and *Saurolophini*.

### *Camptosauridae* Marsh, 1885 (converted clade name)

**Registration number:** 595

**Definition.** The largest clade containing *Camptosaurus dispar* (Marsh, 1879) but not *Iguanodon bernissartensis* Boulenger in Beneden, 1881. This is a maximum-clade definition. Abbreviated definition: max ∇ (*Camptosaurus dispar* (Marsh, 1879) ~ *Iguanodon bernissartensis* Boulenger in Beneden, 1881).

**Reference phylogeny.** Figure 13 of Madzia, Jagt & Mulder (2020) is treated here as the primary reference phylogeny. Additional reference phylogenies include Figure 20 of Verdú et al. (2018), Figure 11 of Santos-Cubedo et al. (2021), and Figure 9 of Verdú et al. (2020).

**Composition.** Under the primary reference phylogeny, *Camptosauridae* comprises *Camptosaurus dispar* and *Cumnoria prestwichii*. Under alternative hypotheses, however, *Camptosauridae* includes only a single unequivocal member, *Camptosaurus dispar* (*e.g*., Madzia, Jagt & Mulder, 2020: Fig. 12).

**Synonyms.** No other taxon names are currently in use for the same or approximate clade.

**Comments.** The name *Camptosauridae* was first (informally) defined by Sereno (1998: 62) who used the maximum-clade definition and selected *Camptosaurus* as the internal specifier and *Parasaurolophus* as the external specifier. We prefer to use *Iguanodon bernissartensis* as the external specifier to maintain the ‘node-branch triplet’ (‘node-stem triplet’ of Sereno (1998: 52–54)) comprising *Ankylopollexia*, *Camptosauridae*, and *Styracosterna* (all formally defined in the present paper). The inclusion of a different external specifier does not change the extent of *Camptosauridae* under any of the published phylogeny inferences.

### *Centrosaurinae* Lambe, 1915 (converted clade name)

**Registration number:** 596

**Definition.** The largest clade containing *Centrosaurus apertus* Lambe, 1905 but not *Chasmosaurus belli* (Lambe, 1902) and *Triceratops horridus* Marsh, 1889. This is a maximum-clade definition. Abbreviated definition: max ∇ (*Centrosaurus apertus* Lambe, 1905 ~ *Chasmosaurus belli* (Lambe, 1902) & *Triceratops horridus* Marsh, 1889).

**Reference phylogeny.** Figure 9 of Chiba et al. (2018) is treated here as the primary reference phylogeny. Additional reference phylogenies include Figure 10 of Ryan et al. (2017), Figure 13 of Dalman et al. (2018), Figure 10 of Wilson, Ryan & Evans (2020), Figure 4 of Yu et al. (2020), and Figure 23 of Dalman et al. (2021).

**Composition.** Under the primary reference phylogeny, *Centrosaurinae* comprises *Albertaceratops nesmoi*, *Diabloceratops eatoni*, *Machairoceratops cronusi*, *Medusaceratops lokii*, *Sinoceratops zhuchengensis*, *Wendiceratops pinhornensis*, *Xenoceratops foremostensis*, and members of the clades *Eucentrosaura* and *Nasutoceratopsini*.

**Synonyms.** No other taxon names are currently in use for the same or approximate clade. Although *Ceratops montanus* may fall within the largest clade containing *Centrosaurus apertus* but not *Chasmosaurus belli* and *Triceratops horridus* as well, the name *Ceratopsinae* Abel, 1919 has not been associated with the same contents as *Centrosaurinae* in the past. Therefore, *Ceratopsinae* is not considered to be an approximate synonym of *Centrosaurinae*. In any case, *C. montanus* does not seem to be diagnostic beyond *Ceratopsidae* at present (Dodson, Forster & Sampson, 2004; Mallon et al., 2016). Therefore, its position within the clade is uncertain. Lucas et al. (2016: 202) have argued that *Pachyrhinosaurinae* von Huene, 1950 has priority over *Centrosaurinae* under the Article 61 of the ICZN (International Commission on Zoological Nomenclature, 1999). However, the name *Pachyrhinosaurinae* has not been used in the literature recently and even Lucas et al. (2016) used *Centrosaurinae* for the clade in question.

**Comments.** The name *Centrosaurinae* has been (informally) defined before (Sereno, 1998; Dodson, Forster & Sampson, 2004; Sereno, 2005). These definitions were maximum-clade and used *Pachyrhinosaurus* (Sereno, 1998), *Centrosaurus* (Dodson, Forster & Sampson, 2004), or *Centrosaurus apertus* (Sereno, 2005) as the internal specifier and *Triceratops* (Sereno, 1998; Dodson, Forster & Sampson, 2004) or *Triceratops horridus* (Sereno, 2005) as the external specifier. We apply the name *Centrosaurinae* for the same known contents; adopting the mandatory *Centrosaurus apertus* as the internal specifier and *Chasmosaurus belli* and *Triceratops horridus* as the external specifiers.

### *Centrosaurini* Ryan et al., 2017 (converted clade name)

**Registration number:** 687

**Definition.** The largest clade containing *Centrosaurus apertus* Lambe, 1905 but not *Pachyrhinosaurus canadensis* Sternberg, 1950. This is a maximum-clade definition. Abbreviated definition: max ∇ (*Centrosaurus apertus* Lambe, 1905 ~ *Pachyrhinosaurus canadensis* Sternberg, 1950).

**Reference phylogeny.** Figure 9 of Chiba et al. (2018) is treated here as the primary reference phylogeny. Additional reference phylogenies include Figure 7 of Fiorillo & Tykoski (2012), Figure 10 of Ryan et al. (2017), Figure 13 of Dalman et al. (2018), and Figure 23 of Dalman et al. (2021).

**Composition.** Under the primary reference phylogeny, *Centrosaurini* comprises *Centrosaurus apertus*, *Coronosaurus brinkmani*, *Rubeosaurus ovatus* (?= *Styracosaurus albertensis*; see Holmes et al., 2020), *Spinops sternbergorum*, and *Styracosaurus albertensis*. Under an alternative hypothesis, *Centrosaurini* includes only a single unequivocal member, *Centrosaurus apertus* (Wilson, Ryan & Evans, 2020: Fig. 10). However, a Bayesian analysis of the same matrix and published in the same study reconstructed *Centrosaurini* to comprise *Centrosaurus apertus*, *Coronosaurus brinkmani*, and *Spinops sternbergorum* (Wilson, Ryan & Evans, 2020: Fig. 9).

**Synonyms.** No other taxon names are currently in use for the same or approximate clade.

**Comments.** The name was first (informally) defined by Ryan et al. (2017) who applied the maximum-clade definition and used *Centrosaurus apertus* as the internal specifier and *Pachyrhinosaurus canadensis* as the external specifier. We formalize this definition.

### *Cerapoda* Sereno, 1986 (converted clade name)

**Registration number:** 597

**Definition.** The smallest clade containing *Iguanodon bernissartensis* Boulenger in Beneden, 1881, *Pachycephalosaurus wyomingensis* (Gilmore, 1931), and *Triceratops horridus* Marsh, 1889. This is a minimum-clade definition. Abbreviated definition: min ∇ (*Iguanodon bernissartensis* Boulenger in Beneden, 1881 & *Pachycephalosaurus wyomingensis* (Gilmore, 1931) & *Triceratops horridus* Marsh, 1889).

**Reference phylogeny.** Figure 4 of Madzia, Boyd & Mazuch (2018) is treated here as the primary reference phylogeny. Additional reference phylogenies include Figure 16 of Han et al. (2018), Figure 25 of Herne et al. (2019), Figure 1 of Dieudonné et al. (2020), and Figure 57 of Barta & Norell (2021).

**Composition.** Under the primary reference phylogeny, *Cerapoda* comprises members of the clades *Ornithopoda* and *Marginocephalia*.

**Synonyms.** No other taxon names are currently in use for the same or approximate clade.

**Comments.** The name *Cerapoda* has been (informally) defined before (Weishampel, 2004; Butler, Upchurch & Norman, 2008). Both types of definitions, minimum-clade as well as maximum-clade, have been proposed for the name. Weishampel (2004) preferred a maximum-clade definition and used *Triceratops* as the internal specifier and *Ankylosaurus* as the external specifier, while Butler, Upchurch & Norman (2008) applied a minimum-clade definition, using *Triceratops horridus* and *Parasaurolophus walkeri* as the internal specifiers. Subsequent authors followed the latter definition (Boyd, 2015; Madzia, Boyd & Mazuch, 2018; Herne et al., 2019; Yang et al., 2020). We apply a minimum-clade definition as well and use *Iguanodon bernissartensis*, *Pachycephalosaurus wyomingensis*, and *Triceratops horridus* as the internal specifiers. Note that the internal specifiers *Pachycephalosaurus wyomingensis* and *Triceratops horridus* are not included in the primary reference phylogeny. The former belongs to *Pachycephalosauria* (see, *e.g*., Dieudonné et al., 2020), while the latter is part of *Ceratopsia* (*e.g*., Morschhauser et al., 2019), both within *Marginocephalia* that is indicated on Figure 4 of Madzia, Boyd & Mazuch (2018).

### *Ceratopsia* Marsh, 1890 (converted clade name)

**Registration number:** 598

**Definition.** The largest clade containing *Ceratops montanus* Marsh, 1888 and *Triceratops horridus* Marsh, 1889 but not *Pachycephalosaurus wyomingensis* (Gilmore, 1931). This is a maximum-clade definition. Abbreviated definition: max ∇ (*Ceratops montanus* Marsh, 1888 & *Triceratops horridus* Marsh, 1889 ~ *Pachycephalosaurus wyomingensis* (Gilmore, 1931)).

**Reference phylogeny.** Figure 10 of Morschhauser et al. (2019) is treated here as the primary reference phylogeny. Additional reference phylogenies include Figure 16 of Han et al. (2018), Figure S1 of Knapp et al. (2018), Figure 1 of Dieudonné et al. (2020), Figure 3 of Yu et al. (2020), and Figure 4 of Yu et al. (2020).

**Composition.** Under the primary reference phylogeny, *Ceratopsia* comprises *Psittacosaurus* spp. and members of the clades *Chaoyangsauridae* and *Neoceratopsia*.

**Synonyms.** No other taxon names are currently in use for the same or approximate clade.

**Comments.** The name *Ceratopsia* has been (informally) defined before (Dodson, 1997; Sereno, 1998; Sereno, 2005). These definitions were maximum-clade and used *Ceratopsidae* (Dodson, 1997), *Triceratops* (Sereno, 1998), or *Triceratops horridus* (Sereno, 2005) as the internal specifiers and *Pachycephalosauridae* (Dodson, 1997), *Pachycephalosaurus* (Sereno, 1998), or *Pachycephalosaurus wyomingensis*, *Heterodontosaurus tucki*, *Hypsilophodon foxii*, and *Ankylosaurus magniventris* (Sereno, 2005) as the external specifiers. Even though the position of *Hypsilophodon foxii* and *Heterodontosaurus tucki* is indeed somewhat unstable across studies (see, *e.g*., Han et al., 2018; Madzia, Boyd & Mazuch, 2018; Herne et al., 2019; Dieudonné et al., 2020; Yang et al., 2020), inclusion of these taxa among the external specifiers is not necessary. We use a definition similar to that of Sereno (1998) but include the mandatory *Ceratops montanus* as a second internal specifier. Note that the internal specifier *Ceratops montanus* and the external specifier *Pachycephalosaurus wyomingensis* are not included in the primary reference phylogeny. The former belongs to *Ceratopsidae* (*e.g*., Mallon et al., 2016), while the latter is part of *Pachycephalosauria* (see, *e.g*., Dieudonné et al., 2020).

### *Ceratopsidae* Marsh, 1888 (converted clade name)

**Registration number:** 599

**Definition.** The smallest clade containing *Centrosaurus apertus* Lambe, 1905, *Ceratops montanus* Marsh, 1888, *Chasmosaurus belli* (Lambe, 1902), and *Triceratops horridus* Marsh, 1889. This is a minimum-clade definition. Abbreviated definition: min ∇ (*Centrosaurus apertus* Lambe, 1905 & *Ceratops montanus* Marsh, 1888 & *Chasmosaurus belli* (Lambe, 1902) & *Triceratops horridus* Marsh, 1889).

**Reference phylogeny.** Figure 4 of Yu et al. (2020) is treated here as the primary reference phylogeny. Additional reference phylogenies include Figure 14 of Mallon et al. (2016), Figure S1 of Knapp et al. (2018), Figure 9a of Fowler & Freedman Fowler (2020), Figure 10 of Wilson, Ryan & Evans (2020), and Figure 3 of Yu et al. (2020).

**Composition.** Under the primary reference phylogeny, *Ceratopsidae* comprises members of the clades *Centrosaurinae* and *Chasmosaurinae*.

**Synonyms.** No other taxon names are currently in use for the same or approximate clade.

**Comments.** The name *Ceratopsidae* has been (informally) defined before (Sereno, 1998, Dodson, Forster & Sampson, 2004; Sereno, 2005). These definitions were minimum-clade and used *Triceratops* and *Pachyrhinosaurus* (Sereno, 1998), *Triceratops* and *Centrosaurus* (Dodson, Forster & Sampson, 2004), and *Triceratops horridus* and *Pachyrhinosaurus canadensis* (Sereno, 2005) as the internal specifiers. Considering that *Ceratopsidae* ‘traditionally’ contains two subclades, *Centrosaurinae* and *Chasmosaurinae*, we include the nomenclatural types of these clades, *Centrosaurus apertus* and *Chasmosaurus belli*, respectively, as the internal specifiers, and additionally add *Triceratops horridus*, a common specifier in the nomenclature of ceratopsian clades and the only taxon that has always been used as an internal specifier in the definition of *Ceratopsidae*. Finally, we also include a fourth internal specifier, the mandatory *Ceratops montanus*. Even though the taxon is considered a *nomen dubium* (*e.g*., Dodson, Forster & Sampson, 2004; Mallon et al., 2016), its placement within the smallest clade comprising centrosaurines and chasmosaurines does not appear to be questionable (see, *e.g*., Mallon et al., 2016).

### *Ceratopsoidea* Hay, 1902 (converted clade name)

**Registration number:** 601

**Definition.** The largest clade containing *Ceratops montanus* Marsh, 1888 and *Triceratops horridus* Marsh, 1889 but not *Protoceratops andrewsi* Granger & Gregory, 1923. This is a maximum-clade definition. Abbreviated definition: max ∇ (*Ceratops montanus* Marsh, 1888 & *Triceratops horridus* Marsh, 1889 ~ *Protoceratops andrewsi* Granger & Gregory, 1923).

**Reference phylogeny.** Figure 4 of Yu et al. (2020) is treated here as the primary reference phylogeny. Additional reference phylogenies include Figure S1 of Knapp et al. (2018), Figure 10 of Morschhauser et al. (2019), and Figure 3 of Yu et al. (2020).

**Composition.** Under the primary reference phylogeny, *Ceratopsoidea* comprises *Turanoceratops tardabilis*, *Zuniceratops christopheri*, and members of the clade *Ceratopsidae*.

**Synonyms.** No other taxon names are currently in use for the same or approximate clade.

**Comments.** The name *Ceratopsoidea* has been (informally) defined before by Sereno (1998, 2005) who applied a maximum-clade definition and used *Triceratops horridus* as the internal specifier and *Protoceratops andrewsi* as the external specifier. We include an additional internal specifier, the mandatory *Ceratops montanus*.

### *Chaoyangsauridae* Zhao, Cheng & Xu, 1999 (converted clade name)

**Registration number:** 602

**Definition.** The largest clade containing *Chaoyangsaurus youngi* Zhao, Cheng & Xu, 1999 but not *Psittacosaurus mongoliensis* Osborn, 1923 and *Triceratops horridus* Marsh, 1889. This is a maximum-clade definition. Abbreviated definition: max ∇ (*Chaoyangsaurus youngi* Zhao, Cheng & Xu, 1999 ~ *Psittacosaurus mongoliensis* Osborn, 1923 & *Triceratops horridus* Marsh, 1889).

**Reference phylogeny.** Figure 10 of Morschhauser et al. (2019) is treated here as the primary reference phylogeny. Additional reference phylogenies include Figure 10 of Han et al. (2015), Figure 15 of Han et al. (2018), and Figure 3 of Yu et al. (2020).

**Composition.** Under the primary reference phylogeny, *Chaoyangsauridae* comprises *Chaoyangsaurus youngi*, *Hualianceratops wucaiwanensis*, *Xuanhuaceratops niei*, and *Yinlong downsi*.

**Synonyms.** No other taxon names are currently in use for the same or approximate clade.

**Comments.** The name *Chaoyangsauridae* has been (informally) defined before by Han et al. (2015) who applied a maximum-clade definition and used *Chaoyangsaurus youngi* as the internal specifier and *Triceratops horridus* and *Psittacosaurus mongoliensis* as the external specifiers. We formalize this definition.

### *Chasmosaurinae* Lambe, 1915 (converted clade name)

**Registration number:** 603

**Definition.** The largest clade containing *Chasmosaurus belli* (Lambe, 1902) and *Triceratops horridus* Marsh, 1889 but not *Centrosaurus apertus* Lambe, 1905. This is a maximum-clade definition. Abbreviated definition: max ∇ (*Chasmosaurus belli* (Lambe, 1902) & *Triceratops horridus* Marsh, 1889 ~ *Centrosaurus apertus* Lambe, 1905).

**Reference phylogeny.** Figure 9a of Fowler & Freedman Fowler (2020) is treated here as the primary reference phylogeny. Additional reference phylogenies include Figure 3 of Brown & Henderson (2015), Figure 14 of Mallon et al. (2016), Figure S1 of Knapp et al. (2018), Figure 3 of Campbell et al. (2019), and Figure 4 of Yu et al. (2020).

**Composition.** Under the primary reference phylogeny, *Chasmosaurinae* comprises *Agujaceratops mariscalensis*, *Anchiceratops ornatus*, *Arrhinoceratops brachyops*, *Bravoceratops polyphemus*, *Chasmosaurus* spp., *Coahuilaceratops magnacuerna*, *Kosmoceratops richardsoni*, *Navajoceratops sullivani*, *Pentaceratops sternbergii*, *Terminocavus sealyi*, *Utahceratops gettyi*, *Vagaceratops irvinensis*, and members of the clade *Triceratopsini*.

**Synonyms.** The taxon *Ceratops montanus* may also fall within the largest clade containing *Chasmosaurus belli* and *Triceratops horridus* but not *Centrosaurus apertus* (see, *e.g*., Mallon et al., 2016). In such case, *Ceratopsinae* Abel, 1919 would be an approximate synonym. Though the name has been advocated to be the proper name for the clade (it has been (informally) defined by Sereno, 1998 and Sereno, 2005), it was actually introduced 4 years later than *Chasmosaurinae*. Note that the Principle of Coordination, which would make *Ceratopsinae* attributable to Marsh (1888), rather than to Abel (1919), does not apply under the *ICPN* (see Note 9.15A.3). Therefore, *Ceratopsinae* would not have priority over *Chasmosaurinae* under the *ICPN*. Anyway, *C. montanus* does not seem to be diagnostic beyond *Ceratopsidae* at present (Mallon et al., 2016), and its position within the clade is thus uncertain.

**Comments.** The name *Chasmosaurinae* has been (informally) defined before by Dodson, Forster & Sampson (2004) who applied a maximum-clade definition and used *Triceratops* as the internal specifier and *Centrosaurus* as the external specifier. We apply the name *Chasmosaurinae* for the same known contents; adopting *Triceratops horridus* and the mandatory *Chasmosaurus belli* as the internal specifiers and *Centrosaurus apertus* as the external specifier.

### *Clypeodonta* Norman, 2014 (converted clade name)

**Registration number:** 604

**Definition.** The smallest clade within *Ornithopoda* containing *Edmontosaurus regalis* Lambe, 1917 and *Hypsilophodon foxii* Huxley, 1869. This is a minimum-clade definition. Abbreviated definition: min ∇ ∈ *Ornithopoda* (*Edmontosaurus regalis* Lambe, 1917 & *Hypsilophodon foxii* Huxley, 1869).

**Reference phylogeny.** Figure 50 of Norman (2015) is treated here as the primary reference phylogeny. Additional reference phylogenies include Figure 25 of Herne et al. (2019) and Figure 2 of Dieudonné et al. (2020).

**Composition.** Under the primary reference phylogeny, *Clypeodonta* comprises a clade formed by *Hypsilophodon foxii*, *Rhabdodontidae*, and *Tenontosaurus* spp., and a clade uniting *Dryosauridae* and *Ankylopollexia* (termed *Iguanodontia* in Norman, 2015). However, see ‘Comments’ below for discussion of potential alternative composition of *Clypeodonta*.

**Synonyms.** No other taxon names are currently in use for the same or approximate clade. *Iguanodontia*, as reconstructed, for example, by Madzia, Jagt & Mulder (2020) covers a similar taxic composition; though the topology of Madzia, Jagt & Mulder (2020) differs from that of the primary reference phylogeny of *Clypeodonta* significantly.

**Comments.** The name *Clypeodonta* was claimed as being new in two different studies (Norman, 2014: 29; Norman, 2015: 102), although Norman (2015: 170) also cites Norman (2014) as the establishing reference. The use of the name *Clypeodonta* differed across studies. Originally, Norman (2014, 2015) intended to use it for a subclade of *Ornithopoda* that (approximately) comprises *Hypsilophodon foxii* and its relatives, and ornithopods later-diverging than *H. foxii*, and (informally) defined the name as pertaining to either, the branch of “*Parasaurolophus walkeri* and all taxa more closely related to *P. walkeri* than to *Thescelosaurus neglectus*” (Norman, 2014: 29) or the node of “*Hypsilophodon foxii*, *Edmontosaurus regalis*, their most recent common ancestor, and all of its descendants” (Norman, 2015: 170). In both these studies, *Clypeodonta* is said (Norman, 2014: 29) or figured (Norman, 2015: Fig. 50) to cover the same known contents although neither of the studies included taxa in their analyses that would fall outside the clade (except for *Lesothosaurus diagnosticus*). Madzia, Boyd & Mazuch (2018) followed the definition of Norman (2015). In their phylogenetic analysis, however, the name covers a much broader contents as one of the internal specifiers of *Clypeodonta*, *Hypsilophodon foxii*, is reconstructed outside *Cerapoda* in that study (Madzia, Boyd & Mazuch, 2018: Fig. 4). Still, Madzia, Boyd & Mazuch (2018: Appendix 1) stated that as *Clypeodonta* was a relatively new name with no ‘traditional’ meaning, they saw no reason for its redefinition. They also noted, though, that “given the unstable position of *H. foxii* among neornithischians, the name might have only limited utility” (Madzia, Boyd & Mazuch, 2018: Appendix 1).

Here we define the name *Clypeodonta* using the minimum-clade definition of Norman (2015). However, by including the part “within *Ornithopoda*” in the definition, we restrict the use of *Clypeodonta* only when *H. foxii* represents an ornithopod (see Article 11.14 of the *ICPN*), following the original intent of Norman (2014, 2015).

### *Coronosauria* Sereno, 1986 (converted clade name)

**Registration number:** 605

**Definition.** The smallest clade containing *Protoceratops andrewsi* Granger & Gregory, 1923 and *Triceratops horridus* Marsh, 1889. This is a minimum-clade definition. Abbreviated definition: min ∇ (*Protoceratops andrewsi* Granger & Gregory, 1923 & *Triceratops horridus* Marsh, 1889).

**Reference phylogeny.** Figure 10 of Morschhauser et al. (2019) is treated here as the primary reference phylogeny. Additional reference phylogenies include Figure S1 of Knapp et al. (2018), Figure 8A of Arbour & Evans (2019), Figure 3 of Yu et al. (2020), and Figure 4 of Yu et al. (2020).

**Composition.** Under the primary reference phylogeny, *Coronosauria* comprises members of the clades *Protoceratopsidae* and *Ceratopsoidea*.

**Synonyms.** No other taxon names are currently in use for the same or approximate clade.

**Comments.** The name *Coronosauria* has been (informally) defined before by Sereno (1998, 2005) who applied the minimum-clade definition and used *Triceratops horridus* and *Protoceratops andrewsi* as the internal specifiers. We formalize this definition.

### *Corythosauria* (new clade name)

**Registration number:** 746

**Definition.** The smallest clade containing *Corythosaurus casuarius* Brown, 1914a, *Lambeosaurus lambei* Parks, 1923, and *Parasaurolophus walkeri* Parks, 1922. This is a minimum-clade definition. Abbreviated definition: min ∇ (*Corythosaurus casuarius* Brown, 1914a & *Lambeosaurus lambei* Parks, 1923 & *Parasaurolophus walkeri* Parks, 1922).

**Etymology.** Derived from the stem of *Corythosaurus* Brown, 1914a, the name of an included taxon, which combines the Greek words *korythos* (helmet) and *sauros* (lizard, reptile).

**Reference phylogeny.** Figure 18 of Prieto-Márquez, Wagner & Lehman (2020) is treated here as the primary reference phylogeny. Additional reference phylogenies include Figure 5 of Kobayashi et al. (2019), Figure 11 of Prieto-Márquez et al. (2019), Figure 9 of Zhang et al. (2019), Figure 5 of Zhang et al. (2020), Figure 7 of Kobayashi et al. (2021), and Figure 10 of Longrich et al. (2021).

**Composition.** Under the primary reference phylogeny, *Corythosauria* comprises members of the clades *Lambeosaurini* and *Parasaurolophini*.

**Synonyms.** No other taxon names are currently in use for the same or approximate clade.

**Comments.** The name *Corythosauria* is established for the well-supported node uniting *Lambeosaurini* and *Parasaurolophini*, two lambeosaurine clades characterized by their distinctive, ‘crested’ crania.

### *Dryomorpha* Sereno, 1986 (converted clade name)

**Registration number:** 606

**Definition.** The smallest clade containing *Dryosaurus altus* (Marsh, 1878) and *Iguanodon bernissartensis* Boulenger in Beneden, 1881. This is a minimum-clade definition. Abbreviated definition: min ∇ (*Dryosaurus altus* (Marsh, 1878) & *Iguanodon bernissartensis* Boulenger in Beneden, 1881).

**Reference phylogeny.** Figure 12 of Madzia, Jagt & Mulder (2020) is treated here as the primary reference phylogeny. Additional reference phylogenies include Figure 20 of Verdú et al. (2018), Figure 2 of Dieudonné et al. (2020), Figure 9 of Verdú et al. (2020), and Figure 11 of Santos-Cubedo et al. (2021).

**Composition.** Under the primary reference phylogeny, *Dryomorpha* comprises members of the clades *Dryosauridae* and *Ankylopollexia*.

**Synonyms.** No other taxon names are currently in use for the same or approximate clade.

**Comments.** The name *Dryomorpha* was first (informally) defined by Sereno (2005) who attributed the name to “(t)he most inclusive clade containing *Dryosaurus altus* (Marsh, 1878) and *Parasaurolophus walkeri* Parks, 1922”. However, due to the use of ‘most’, rather than ‘least’, such definition makes the name inapplicable within *Ornithischia*. Boyd (2015) later corrected the wording and proposed a minimum-clade definition using the same taxa as the internal specifiers. Here we use the same type of definition but replace *P. walkeri* with *I. bernissartensis*. This taxon has always been considered a part of *Dryomorpha*.

### *Dryosauridae* Milner & Norman, 1984 (converted clade name)

**Registration number:** 607

**Definition.** The largest clade containing *Dryosaurus altus* (Marsh, 1878) but not *Iguanodon bernissartensis* Boulenger in Beneden, 1881. This is a maximum-clade definition. Abbreviated definition: max ∇ (*Dryosaurus altus* (Marsh, 1878) ~ *Iguanodon bernissartensis* Boulenger in Beneden, 1881).

**Reference phylogeny.** Figure 12 of Madzia, Jagt & Mulder (2020) is treated here as the primary reference phylogeny. Additional reference phylogenies include Figure 20 of Verdú et al. (2018), Figure 9 of Verdú et al. (2020), Figure 57 of Barta & Norell (2021), and Figure 11 of Santos-Cubedo et al. (2021).

**Composition.** Under the primary reference phylogeny, *Dryosauridae* comprises *Callovosaurus leedsi*, ‘*Camptosaurus*’ *valdensis*, *Dryosaurus altus*, *Dysalotosaurus lettowvorbecki*, *Elrhazosaurus nigeriensis*, *Eousdryosaurus nanohallucis*, and *Valdosaurus canaliculatus*.

**Synonyms.** No other taxon names are currently in use for the same or approximate clade.

**Comments.** *Dryosauridae* was first (informally) defined by Sereno (1998: 61) who used the maximum-clade definition and *Dryosaurus altus* as the internal specifier and *Parasaurolophus walkeri* as the external specifier. Here we use the same type of definition but replace *P. walkeri* with *I. bernissartensis*. This taxon has always been considered outside *Dryosauridae*.

### *Edmontosaurini* Glut, 1997 (converted clade name)

**Registration number:** 608

**Definition.** The largest clade containing *Edmontosaurus regalis* Lambe, 1917 but not *Brachylophosaurus canadensis* Sternberg, 1953, *Hadrosaurus foulkii* Leidy, 1858, *Kritosaurus navajovius* Brown, 1910, and *Saurolophus osborni* Brown, 1912. This is a maximum-clade definition. Abbreviated definition: max ∇ (*Edmontosaurus regalis* Lambe, 1917 ~ *Brachylophosaurus canadensis* Sternberg, 1953 & *Hadrosaurus foulkii* Leidy, 1858 & *Kritosaurus navajovius* Brown, 1910 & *Saurolophus osborni* Brown, 1912).

**Reference phylogeny.** Figure 18 of Prieto-Márquez, Wagner & Lehman (2020) is treated here as the primary reference phylogeny. Additional reference phylogenies include Figure 5 of Kobayashi et al. (2019), Figure 11 of Prieto-Márquez et al. (2019), Figure 9 of Zhang et al. (2019), Figure 5 of Zhang et al. (2020), Figure 7 of Kobayashi et al. (2021), and Figure 10 of Longrich et al. (2021).

**Composition.** Under the primary reference phylogeny, *Edmontosaurini* comprises *Edmontosaurus* spp., *Kerberosaurus manakini*, *Kundurosaurus nagornyi*, and *Shantungosaurus giganteus*.

**Synonyms.** No other taxon names are currently in use for the same or approximate clade.

**Comments.** The name *Edmontosaurini* has been (informally) defined before (Sereno, 2005; Xing et al., 2014). Sereno (2005) applied the maximum-clade definition and used *Edmontosaurus regalis* as the internal specifier and *Maiasaura peeblesorum* and *Saurolophus osborni* as the external specifiers. In turn, Xing et al. (2014) applied a minimum-clade definition, with *Edmontosaurus* and *Kerberosaurus* as the internal specifiers. We formalize a maximum-clade definition similar to that of Sereno (2005) but replace *M. peeblesorum* with *Brachylophosaurus canadensis*, as the representative of *Brachylophosaurini*, and further add *Kritosaurus navajovius* and *Hadrosaurus foulkii*.

### *Elasmaria* Calvo, Porfiri & Novas, 2007 (converted clade name)

**Registration number:** 609

**Definition.** The smallest clade containing *Macrogryphosaurus gondwanicus* Calvo, Porfiri & Novas, 2007 and *Talenkauen santacrucensis* Novas, Cambiaso & Ambrosio, 2004, provided that it does not include *Hypsilophodon foxii* Huxley, 1869, *Iguanodon bernissartensis* Boulenger in Beneden, 1881, or *Thescelosaurus neglectus* Gilmore, 1913. This is a minimum-clade definition. Abbreviated definition: min ∇ (*Macrogryphosaurus gondwanicus* Calvo, Porfiri & Novas, 2007 & *Talenkauen santacrucensis* Novas, Cambiaso & Ambrosio, 2004 | ~ *Hypsilophodon foxii* Huxley, 1869 ∨ *Iguanodon bernissartensis* Boulenger in Beneden, 1881 ∨ *Thescelosaurus neglectus* Gilmore, 1913).

**Reference phylogeny.** Figure 31 of Rozadilla, Agnolín & Novas, 2019 is treated here as the primary reference phylogeny. Additional reference phylogenies include Figure 4 of Madzia, Boyd & Mazuch (2018), Figure 26 of Herne et al. (2019), Figure 2 of Dieudonné et al. (2020), and Figure 57 of Barta & Norell (2021).

**Composition.** Under the primary reference phylogeny, *Elasmaria* comprises *Anabisetia saldiviai*, *Atlascopcosaurus loadsi*, *Fulgurotherium austral*, *Gasparinisaura cincosaltensis*, *Kangnasaurus coetzeei*, *Macrogryphosaurus gondwanicus*, *Morrosaurus antarcticus*, *Notohypsilophodon comodorensis*, *Quantassaurus intrepidus*, and *Trinisaura santamartaensis*.

**Synonyms.** No other taxon names are currently in use for the same or approximate clade.

**Comments.** The name *Elasmaria* has been (informally) defined before (Calvo, Porfiri & Novas, 2007; Herne et al., 2019). The definition proposed by Calvo, Porfiri & Novas (2007) was minimum-clade, while the definition of Herne et al. (2019) was maximum-clade. However, both studies used *Talenkauen santacrucensis* and *Macrogryphosaurus gondwanicus* as the internal specifiers. Herne et al. (2019) proposed to add *Iguanodon bernissartensis* and *Hypsilophodon foxii* as the external specifiers to maintain the use of the name *Elasmaria* to the ‘traditional’ contents under a hypothesis in which one of the internal specifiers was reconstructed, for example, closer to iguanodontians. We keep the use of a minimum-clade definition (as first proposed for the name). However, even though all phylogenetic analyses consistently reconstruct close relationships between *T. santacrucensis* and *M. gondwanicus*, we follow Herne et al. (2019) in that the unsettled placement of elasmarians on the neornithischian phylogenetic tree warrants addition of external specifiers. We include *Iguanodon bernissartensis* and *Hypsilophodon foxii* as the external specifiers (following Herne et al., 2019) and further add a third external specifier, *Thescelosaurus neglectus*, to reflect that elasmarians were already inferred as a clade within *Thescelosaurinae*, as the sister taxon to *Thescelosaurus* spp. (Boyd, 2015).

### *Eucentrosaura* Chiba et al., 2018 (converted clade name)

**Registration number:** 688

**Definition.** The smallest clade containing *Centrosaurus apertus* Lambe, 1905 and *Pachyrhinosaurus canadensis* Sternberg, 1950. This is a minimum-clade definition. Abbreviated definition: min ∇ (*Centrosaurus apertus* Lambe, 1905 & *Pachyrhinosaurus canadensis* Sternberg, 1950).

**Reference phylogeny.** Figure 9 of Chiba et al. (2018) is treated here as the primary reference phylogeny. Additional reference phylogenies include Figure 7 of Fiorillo & Tykoski (2012), Figure 10 of Ryan et al. (2017), Figure 13 of Dalman et al. (2018), and Figure 23 of Dalman et al. (2021).

**Composition.** Under the primary reference phylogeny, *Eucentrosaura* comprises members of the clades *Centrosaurini* and *Pachyrhinosaurini*.

**Synonyms.** No other taxon names are currently in use for the same or approximate clade.

**Comments.** The name was first (informally) defined by Chiba et al. (2018) who applied the minimum-clade definition and used *Centrosaurus apertus* and *Pachyrhinosaurus canadensis* as the internal specifiers. We formalize this definition.

### *Euceratopsia* (new clade name)

**Registration number:** 610

**Definition.** The smallest clade containing *Leptoceratops gracilis* Brown, 1914b, *Protoceratops andrewsi* Granger & Gregory, 1923, and *Triceratops horridus* Marsh, 1889. This is a minimum-clade definition. Abbreviated definition: min ∇ (*Leptoceratops gracilis* Brown, 1914b & *Protoceratops andrewsi* Granger & Gregory, 1923 & *Triceratops horridus* Marsh, 1889).

**Etymology.** Derived from the Greek *eu*- (true) and formed to show its association to members of *Ceratopsia*. Note that *Euceratopsia* does not derive from the name *Ceratops* Marsh, 1888, and, as such, the taxon does not have to be the internal specifier in the used definition.

**Reference phylogeny.** Figure 4 of Yu et al. (2020) is treated here as the primary reference phylogeny. Additional reference phylogenies include Figure 16 of Han et al. (2018), Figure S1 of Knapp et al. (2018), Figure 10 of Morschhauser et al. (2019), and Figure 3 of Yu et al. (2020).

**Composition.** Under the primary reference phylogeny, *Euceratopsia* comprises members of the clades *Leptoceratopsidae* and *Coronosauria*.

**Synonyms.** The name *Coronosauria* Sereno, 1986 covers the same contents under the topology of You & Dodson (2004). However, see ‘Comments’. No other taxon names are currently in use for the same or approximate clade.

**Comments.** The name *Euceratopsia* is established for the well-supported node uniting the three latest-diverging clades of ceratopsians – *Leptoceratopsidae*, *Protoceratopsidae*, and *Ceratopsoidea*. The monophyly of the grouping is supported by all recently published phylogenies that infer *Euceratopsia* to branch into two clades – leptoceratopsids and coronosaurs (protoceratopsids + ceratopsoids). Both these clades comprise representatives that are very close or survived to the Cretaceous/Paleogene mass extinction event (Fowler, 2017: Table S1). It is worth noting that You & Dodson (2004) reconstructed leptoceratopsids to be the sister taxon to *Ceratopsoidea*, and *Protoceratopsidae* to be the sister taxon to *Leptoceratopsidae* + *Ceratopsoidea*. Under such topology, *Euceratopsia* becomes a heterodefinitional synonym of *Coronosauria*, with the latter having priority.

### *Euhadrosauria* Weishampel, Norman & Grigorescu, 1993 (converted clade name)

**Registration number:** 611

**Definition.** The smallest clade containing *Lambeosaurus lambei* Parks, 1923 and *Saurolophus osborni* Brown, 1912, provided that it does not include *Hadrosaurus foulkii* Leidy, 1858. This is a minimum-clade definition. Abbreviated definition: min ∇ (*Lambeosaurus lambei* Parks, 1923 & *Saurolophus osborni* Brown, 1912 | ~ *Hadrosaurus foulkii* Leidy, 1858).

**Reference phylogeny.** Figure 18 of Prieto-Márquez, Wagner & Lehman (2020) is treated here as the primary reference phylogeny. Additional reference phylogenies include Figure 11 of Prieto-Márquez et al. (2019), Figure 9 of Zhang et al. (2019), Figure 7 of Kobayashi et al. (2021), Figure 10 of Longrich et al. (2021), and Figure 11 of McDonald et al. (2021).

**Composition.** Under the primary reference phylogeny, *Euhadrosauria* comprises members of the clades *Saurolophinae* and *Lambeosaurinae*.

**Synonyms.** The name *Hadrosauridae* Cope, 1869 is an approximate synonym of *Euhadrosauria*. If *Hadrosaurus foulkii* nests within the smallest clade containing *Saurolophus osborni* and *Lambeosaurus lambei*, and within the ‘*Saurolophus* branch’ of the clade (see the entry for the name *Saurolophinae*), the name *Hadrosauridae* is used for the node instead, and *Euhadrosauria* becomes inapplicable. Additionally, the name *Saurolophidae* has been used for the same contents as well (see ‘Comments’).

**Comments.** The history and application of *Euhadrosauria* is complicated and has been thoroughly described and discussed by Madzia, Jagt & Mulder (2020: 14–16). We therefore refer to that study for details.

### *Euiguanodontia* Coria & Salgado, 1996 (converted clade name)

**Registration number:** 612

**Definition.** The smallest clade containing *Camptosaurus dispar* (Marsh, 1879), *Dryosaurus altus* (Marsh, 1878), and *Gasparinisaura cincosaltensis* Coria & Salgado, 1996, provided that it does not include *Tenontosaurus tilletti* Ostrom, 1970. This is a minimum-clade definition. Abbreviated definition: min ∇ (*Camptosaurus dispar* (Marsh, 1879) & *Dryosaurus altus* (Marsh, 1878) & *Gasparinisaura cincosaltensis* Coria & Salgado, 1996 | ~ *Tenontosaurus tilletti* Ostrom, 1970).

**Reference phylogeny.** Figure 13 of Coria & Salgado (1996) is treated here as the primary reference phylogeny.

**Composition.** Under the primary reference phylogeny, *Euiguanodontia* comprises *Gasparinisaura* and members of the clades *Dryosauridae* and *Ankylopollexia*.

**Synonyms.** No other taxon names are currently in use for the same or approximate clade.

**Comments.** The name *Euiguanodontia* is applicable only on the condition that *G. cincosaltensis*, *D. altus*, and *C. dispar* form a clade exclusive of *T. tilletti*, as originally used by Coria & Salgado (1996). We follow the definition advocated by Madzia, Boyd & Mazuch (2018: Appendix 1) and refer to that study for additional comments. Note also that *Euiguanodontia* must be a subclade of *Iguanodontia* under the proposed definition because *T. tilletti* is an internal specifier in the definition of the name. Finally, note that the internal specifiers *Dryosaurus altus* and *Camptosaurus dispar* are not included in the primary reference phylogeny. The former belongs to *Dryosauridae* (*e.g*., Madzia, Boyd & Mazuch, 2018), while the latter is part of *Ankylopollexia* (see, *e.g*., Madzia, Jagt & Mulder, 2020). Both these clades are indicated on Figure 13 of Coria & Salgado (1996).

### *Euornithopoda* Sereno, 1986 (converted clade name)

**Registration number:** 613

**Definition.** The largest clade within *Ornithopoda* containing *Iguanodon bernissartensis* Boulenger in Beneden, 1881 but not *Heterodontosaurus tucki* Crompton & Charig, 1962. This is a maximum-clade definition. Abbreviated definition: max ∇ ∈ *Ornithopoda* (*Iguanodon bernissartensis* Boulenger in Beneden, 1881 ~ *Heterodontosaurus tucki* Crompton & Charig, 1962).

**Reference phylogeny.** Figure 1 of Sereno (1999) is treated here as the primary reference phylogeny.

**Composition.** Under the primary reference phylogeny, *Euornithopoda* comprises *Tenontosaurus* spp. and members of the clades *Ankylopollexia*, *Dryosauridae*, and *Hypsilophodontidae*.

**Synonyms.** No other taxon names are currently in use for the same or approximate clade.

**Comments.** The name *Euornithopoda* has been (informally) defined before (Sereno, 1998; Sereno, 2005). These definitions were maximum-clade and used *Parasaurolophus* as the internal specifier and *Heterodontosaurus tucki*, *Pachycephalosaurus wyomingensis*, *Triceratops horridus*, and *Ankylosaurus magniventris* (Sereno, 2005) as the external specifiers. Here we define the name *Euornithopoda* using a similar maximum-clade definition as that of Sereno (1998) but replace *Parasaurolophus* with *Iguanodon bernissartensis*. Also, by including the part “within *Ornithopoda*” in the definition, we restrict the use of *Euornithopoda* to the branch only when *Heterodontosaurus tucki* represents an ornithopod (see Article 11.14 of the *ICPN*), thus maintaining the ‘traditional’ use (Sereno, 1998; Sereno, 2005).

### *Eurypoda* Sereno, 1986 (converted clade name)

**Registration number:** 614

**Definition.** The smallest clade containing *Ankylosaurus magniventris* Brown, 1908 and *Stegosaurus stenops* Marsh, 1887. This is a minimum-clade definition. Abbreviated definition: min ∇ (*Ankylosaurus magniventris* Brown, 1908 & *Stegosaurus stenops* Marsh, 1887).

**Reference phylogeny.** Figure 3 of Thompson et al. (2012) is treated here as the primary reference phylogeny. Additional reference phylogenies include Figure 16 of Han et al. (2018) and Figure 1 of Dieudonné et al. (2020).

**Composition.** Under the primary reference phylogeny, *Eurypoda* comprises members of the clades *Ankylosauria* and *Stegosauria*.

**Synonyms.** No other taxon names are currently in use for the same or approximate clade.

**Comments.** The name *Eurypoda* has been (informally) defined before by Sereno (1998) who used *Ankylosaurus* and *Stegosaurus* as the internal specifiers. Since *Eurypoda* has never been proposed an alternative use, we formalize this definition. Note that the internal specifier *Stegosaurus stenops* is not included in the primary reference phylogeny. The taxon is most closely related to the clade comprising the operational taxonomic units (OTUs) *Stegosaurus armatus* (*nomen dubium* according to Galton, 2010; *S. armatus* has long been the type species of *Stegosaurus* but was replaced by *S. stenops* as the type through an *ICZN* ruling (International Commission on Zoological Nomenclature, 2013)) and *Huayangosaurus taibaii* (see, *e.g*., Maidment et al., 2020).

### *Genasauria* Sereno, 1986 (converted clade name)

**Registration number:** 615

**Definition.** The smallest clade containing *Ankylosaurus magniventris* Brown, 1908, *Iguanodon bernissartensis* Boulenger in Beneden, 1881, *Stegosaurus stenops* Marsh, 1887, and *Triceratops horridus* Marsh, 1889. This is a minimum-clade definition. Abbreviated definition: min ∇ (*Ankylosaurus magniventris* Brown, 1908 & *Iguanodon bernissartensis* Boulenger in Beneden, 1881 & *Stegosaurus stenops* Marsh, 1887 & *Triceratops horridus* Marsh, 1889).

**Reference phylogeny.** Figure 16 of Han et al. (2018) is treated here as the primary reference phylogeny. Additional reference phylogenies include Figure 4 of Madzia, Boyd & Mazuch (2018), Figure 25 of Herne et al. (2019), Figure 1 of Dieudonné et al. (2020), Figure 12 of Yang et al. (2020), and Figure 57 of Barta & Norell (2021).

**Composition.** Under the primary reference phylogeny, *Genasauria* comprises members of the clades *Neornithischia* and *Thyreophora*.

**Synonyms.** No other taxon names are currently in use for the same or approximate clade.

**Comments.** The name *Genasauria* has been (informally) defined before (Currie & Padian, 1997; Sereno, 1998; Sereno, 2005; Butler, Upchurch & Norman, 2008). These definitions were minimum-clade and used *Thyreophora* and *Cerapoda* (Currie & Padian, 1997), *Ankylosaurus* and *Triceratops* (Sereno, 1998), *Ankylosaurus magniventris*, *Triceratops horridus*, and *Parasaurolophus walkeri* (Sereno, 2005), and *Ankylosaurus magniventris*, *Stegosaurus stenops*, *Triceratops horridus*, *Parasaurolophus walkeri*, and *Pachycephalosaurus wyomingensis* (Butler, Upchurch & Norman, 2008) as the internal specifiers. In order to maintain the ‘traditional’ concept of *Genasauria* as a clade comprising *Neornithischia* and *Thyreophora*, the internal specifiers in the definition of *Genasauria* are used from among the taxa representing the four major subclades – *Ornithopoda* (*Iguanodon bernissartensis*), *Marginocephalia* (*Triceratops horridus*), *Ankylosauria* (*Ankylosaurus magniventris*), and *Stegosauria* (*Stegosaurus stenops*). Addition of *P. wyomingensis* as another internal specifier (to include representatives of both marginocephalian clades – *Ceratopsia* and *Pachycephalosauria*) is considered unnecessary because pachycephalosaurs have always been inferred to be part of *Genasauria* as defined herein. Note that the internal specifiers *Ankylosaurus magniventris* and *Triceratops horridus* are not included in the primary reference phylogeny. The former belongs to *Ankylosauria* within *Thyreophora* (see, *e.g*., Thompson et al., 2012), while the latter is part of *Ceratopsia* (*e.g*., Morschhauser et al., 2019).

### *Hadrosauridae* Cope, 1869 (converted clade name)

**Registration number:** 616

**Definition.** The smallest clade containing *Hadrosaurus foulkii* Leidy, 1858, *Lambeosaurus lambei* Parks, 1923, and *Saurolophus osborni* Brown, 1912. This is a minimum-clade definition. Abbreviated definition: min ∇ (*Hadrosaurus foulkii* Leidy, 1858 & *Lambeosaurus lambei* Parks, 1923 & *Saurolophus osborni* Brown, 1912).

**Reference phylogeny.** Figure 18 of Prieto-Márquez, Wagner & Lehman (2020) is treated here as the primary reference phylogeny. Additional reference phylogenies include Figure 5 of Kobayashi et al. (2019), Figure 11 of Prieto-Márquez et al. (2019), Figure 9 of Zhang et al. (2019), Figure 5 of Zhang et al. (2020), Figure 7 of Kobayashi et al. (2021), and Figure 10 of Longrich et al. (2021).

**Composition.** Under the primary reference phylogeny, *Hadrosauridae* comprises *Hadrosaurus foulkii*, *Eotrachodon orientalis*, *Latirhinus uitstlani*, *Aquilarhinus palimentus*, and members of the clade *Euhadrosauria*.

**Synonyms.** Several taxon names have been historically or recently used as approximate synonyms of *Hadrosauridae*. Of these, only the names *Saurolophidae* and *Euhadrosauria* have recently been attributed to a clade of the same or a similar composition (*e.g*., Prieto-Márquez, 2010; Verdú et al., 2018; Zhang et al., 2019; Madzia, Jagt & Mulder, 2020; Prieto-Márquez, Wagner & Lehman, 2020; Verdú et al., 2020; Zhang et al., 2020; Kobayashi et al., 2021; Ramírez-Velasco et al., 2021). See ‘Comments’ below.

**Comments.** The use of *Hadrosauridae* and other names applied to the same or similar clades (*Saurolophidae* and *Euhadrosauria*) have been thoroughly described and discussed by Madzia, Jagt & Mulder (2020: 14–16) who recommended to use *Hadrosauridae* for the smallest clade containing *H. foulkii*, *S. osborni*, and *L. lambei*; *Euhadrosauria* for the smallest clade containing *S. osborni* and *L. lambei*; and to abandon *Saurolophidae*. Note that under some phylogenies, in which *H. foulkii* is reconstructed within the smallest clade containing *S. osborni* and *L. lambei*, the names *Hadrosauridae* and *Euhadrosauria*, as (informally) defined by Madzia, Jagt & Mulder (2020), become heterodefinitional synonyms. Although such option may still be viewed acceptable, we decided to apply a minimum-clade definition for *Euhadrosauria* that makes the name inapplicable under such hypothesis.

### *Hadrosauriformes* Sereno, 1997 (converted clade name)

**Registration number:** 617

**Definition.** The smallest clade containing *Hadrosaurus foulkii* Leidy, 1858 and *Iguanodon bernissartensis* Boulenger in Beneden, 1881. This is a minimum-clade definition. Abbreviated definition: min ∇ (*Hadrosaurus foulkii* Leidy, 1858 & *Iguanodon bernissartensis* Boulenger in Beneden, 1881).

**Reference phylogeny.** Figure 12 of Madzia, Jagt & Mulder (2020) is treated here as the primary reference phylogeny. Additional reference phylogenies include Figure 20 of Verdú et al. (2018), Figure 3 of Párraga & Prieto-Márquez (2019), Figure 8 of Słowiak et al. (2020), Figure 9 of Verdú et al. (2020) and Figure 11 of McDonald et al. (2021).

**Composition.** Under the primary reference phylogeny, *Hadrosauriformes* comprises members of the clades *Iguanodontidae* and *Hadrosauroidea*.

**Synonyms.** If *Hypselospinus fittoni* nests within the smallest clade containing *Hadrosaurus foulkii* and *Iguanodon bernissartensis*, the name *Hadrosauriformes* is a potential heterodefinitional synonym of *Neoiguanodontia* (see the name entry). In such case, the name *Hadrosauriformes* should have priority. The name *Iguanodontoidea* Hay, 1902 has been also used as an approximate synonym (Sereno, 1986; Norman, 2002). Note that Norman (2002) used *Iguanodontoidea* for a clade “(s)erially more derived than *Camptosaurus*” (Norman, 2002: 138) and defined it as “*Iguanodon* and all iguanodontians more closely related to *Edmontosaurus* than to *Camptosaurus*”. Such definition would make *Iguanodontoidea* applicable for the same clade as *Styracosterna* (see the name entry). However, Figure 35 of Norman (2002) shows that the name does not cover *Lurdusaurus*, which should be included within the clade under such maximum-clade definition. Since Norman (2002) considers *Iguanodontoidea* to be a synonym of *Hadrosauriformes* of Sereno (1997, 1998, 1999), it is apparent that Norman (2002) concept of *Iguanodontoidea* would be more similar to that of *Hadrosauriformes* rather than *Styracosterna*.

**Comments.** The name *Hadrosauriformes* has been (informally) defined before (Sereno, 1998; Norman, 2015; Madzia, Jagt & Mulder, 2020). However, only Madzia, Jagt & Mulder (2020: Table 1) included the mandatory *H. foulkii* as the internal specifier. We formalize the definition of Madzia, Jagt & Mulder (2020).

### *Hadrosaurinae* Lambe, 1918 (converted clade name)

**Registration number:** 618

**Definition.** The largest clade containing *Hadrosaurus foulkii* Leidy, 1858 but not *Lambeosaurus lambei* Parks, 1923. This is a maximum-clade definition. Abbreviated definition: max ∇ (*Hadrosaurus foulkii* Leidy, 1858 ~ *Lambeosaurus lambei* Parks, 1923).

**Reference phylogeny.** Figure 5 of Kobayashi et al. (2019) is treated here as the primary reference phylogeny. Additional reference phylogenies include Figure 13 of Cruzado-Caballero & Powell (2017), Figure 20 of Xing, Mallon & Currie (2017), Figure 5 of Zhang et al. (2020), and Figure 10 of Longrich et al. (2021).

**Composition.** Under the primary reference phylogeny, *Hadrosaurinae* comprises *Hadrosaurus foulkii* and members of the clades *Brachylophosaurini*, *Edmontosaurini*, *Kritosaurini*, and *Saurolophini*.

**Synonyms.** The name *Saurolophinae* Brown, 1914a has been recently used for the same clade (under the hypothesis in which *H. foulkii* is nested outside the smallest clade containing *Saurolophus osborni* and *Lambeosaurus lambei*). See the entry for the name *Saurolophinae*.

**Comments.** The name *Hadrosaurinae* has been (informally) defined before by (Sereno, 1998; Sereno, 2005). Sereno (1998) applied the maximum-clade definition and used *Saurolophus* as the internal specifier and *Parasaurolophus* as the external specifier. In turn, Sereno (2005), apparently erroneously, defined *Hadrosaurinae* as pertaining to “(t)he most inclusive taxon containing *Saurolophus osborni* Brown, 1912 and *Parasaurolophus walkeri* Parks, 1922 and including *Hadrosaurus foulkii* Leidy, 1858”. Our formal maximum-clade definition was formed to make *Hadrosaurinae* applicable regardless of whether the taxon lies ouside or within the smallest clade containing *Saurolophus osborni* and *Lambeosaurus lambei*.

### *Hadrosauroidea* von Huene, 1952 (converted clade name)

**Registration number:** 619

**Definition.** The largest clade containing *Hadrosaurus foulkii* Leidy, 1858 but not *Iguanodon bernissartensis* Boulenger in Beneden, 1881. This is a maximum-clade definition. Abbreviated definition: max ∇ (*Hadrosaurus foulkii* Leidy, 1858 ~ *Iguanodon bernissartensis* Boulenger in Beneden, 1881).

**Reference phylogeny.** Figure 12 of Madzia, Jagt & Mulder (2020) is treated here as the primary reference phylogeny. Additional reference phylogenies include Figure 20 of Verdú et al. (2018), Figure 8 of Słowiak et al. (2020), Figure 9 of Verdú et al. (2020), Figure 11 of McDonald et al. (2021), and Figure 11 of Santos-Cubedo et al. (2021).

**Composition.** Under the primary reference phylogeny, *Hadrosauroidea* comprises *Altirhinus kurzanovi*, *Batyrosaurus rozhdestvenskyi*, *Bolong yixianensis*, *Equijubus normani*, *Gongpoquansaurus mazongshanensis*, *Jinzhousaurus yangi*, *Koshisaurus katsuyama*, *Mantellisaurus atherfieldensis*, *Morelladon beltrani*, *Ouranosaurus nigeriensis*, *Penelopognathus weishampeli*, *Proa valdearinnoensis*, *Probactrosaurus gobiensis*, *Ratchasimasaurus suranareae*, *Sirindhorna khoratensis*, *Xuwulong yueluni*, *Zuoyunlong huangi*, and members of the clade *Hadrosauromorpha*.

**Synonyms.** No other taxon names are currently in use for the same or approximate clade.

**Comments.** The name *Hadrosauroidea* was first (informally) defined by Sereno (1998: 62) who used the maximum-clade definition and *Parasaurolophus walkeri* as the internal specifier and *Iguanodon bernissartensis* as the external specifier. We formalize the definition of Madzia, Jagt & Mulder (2020: Table 1) who replaced *P. walkeri* with *H. foulkii*.

### *Hadrosauromorpha* Norman, 2014 (converted clade name)

**Registration number:** 620

**Definition.** The largest clade containing *Hadrosaurus foulkii* Leidy, 1858 but not *Probactrosaurus gobiensis* Rozhdestvensky, 1966. This is a maximum-clade definition. Abbreviated definition: max ∇ (*Hadrosaurus foulkii* Leidy, 1858 ~ *Probactrosaurus gobiensis* Rozhdestvensky, 1966).

**Reference phylogeny.** Figure 12 of Madzia, Jagt & Mulder (2020) is treated here as the primary reference phylogeny. Additional reference phylogenies include Figure 20 of Verdú et al. (2018), Figure 9 of Verdú et al. (2020), Figure 7 of Kobayashi et al. (2021), and Figure 11 of Santos-Cubedo et al. (2021).

**Composition.** Under the primary reference phylogeny, *Hadrosauromorpha* comprises *Bactrosaurus johnsoni*, *Datonglong tianzhenensis*, *Eolambia caroljonesa*, *Gilmoreosaurus mongoliensis*, *Jeyawati rugoculus*, *Jintasaurus meniscus*, *Levnesovia transoxiana*, *Nanyangosaurus zhugeii*, ‘*Orthomerus dolloi*’, *Plesiohadros djadokhtaensis*, *Protohadros byrdi*, *Tanius sinensis*, *Tethyshadros insularis*, *Shuangmiaosaurus gilmorei*, *Zhanghenglong yangchengensis*, and members of the clade *Hadrosauridae*.

**Synonyms.** No other taxon names are currently in use for the same or approximate clade.

**Comments.** *Hadrosauromorpha* was first (informally) defined by Norman (2014: 32) who used the maximum-clade definition and *Parasaurolophus walkeri* as the internal specifier and *Probactrosaurus gobiensis* as the external specifier. We formalize the definition of Madzia, Jagt & Mulder (2020: Table 1) who replaced *P. walkeri* with *H. foulkii*.

### *Heterodontosauridae* Kuhn, 1966 (converted clade name)

**Registration number:** 622

**Definition.** The largest clade containing *Heterodontosaurus tucki* Crompton & Charig, 1962 but not *Iguanodon bernissartensis* Boulenger in Beneden, 1881, *Pachycephalosaurus wyomingensis* (Gilmore, 1931), *Stegosaurus stenops* Marsh, 1887, and *Triceratops horridus* Marsh, 1889. This is a maximum-clade definition. Abbreviated definition: max ∇ (*Heterodontosaurus tucki* Crompton & Charig, 1962 ~ *Iguanodon bernissartensis* Boulenger in Beneden, 1881 & *Pachycephalosaurus wyomingensis* (Gilmore, 1931) & *Stegosaurus stenops* Marsh, 1887 & *Triceratops horridus* Marsh, 1889).

**Reference phylogeny.** Figure 4 of Madzia, Boyd & Mazuch (2018) is treated here as the primary reference phylogeny. Additional reference phylogenies include Figure 25 of Herne et al. (2019), Figure 12 of Yang et al. (2020), and Figure 57 of Barta & Norell (2021).

**Composition.** Under the primary reference phylogeny, *Heterodontosauridae* comprises *Abrictosaurus consors*, *Echinodon becklesii*, *Eocursor parvus*, *Fruitadens haagarorum*, *Heterodontosaurus tucki*, *Lycorhinus angustidens*, *Manidens condorensis*, *Pegomastax africana*, and *Tianyulong confuciusi*.

**Synonyms.** No other taxon names are currently in use for the same or approximate clade.

**Comments.** We follow Sereno (2012) in recognizing Kuhn (1966), rather than Romer (1966), as the author establishing *Heterodontosauridae*. The name *Heterodontosauridae* has been (informally) defined before (Sereno, 1998; Sereno, 2005). These definitions were maximum-clade and used *Heterodontosaurus* as the internal specifier and *Parasaurolophus* (Sereno, 1998) or *Parasaurolophus walkeri*, *Pachycephalosaurus wyomingensis*, *Triceratops horridus*, and *Ankylosaurus magniventris* (Sereno, 2005) as the external specifiers. We apply the name *Heterodontosauridae* for the same known contents; adopting the mandatory *Heterodontosaurus tucki* as the internal specifier and representatives of all major ornithischian lineages, *Ceratopsia* (*Triceratops horridus*), *Ornithopoda* (*Iguanodon bernissartensis*), *Pachycephalosauria* (*Pachycephalosaurus wyomingensis*), and *Thyreophora* (*Stegosaurus stenops*), as the external specifiers. Note that the external specifiers *Pachycephalosaurus wyomingensis*, *Stegosaurus stenops*, and *Triceratops horridus* are not included in the primary reference phylogeny. *P. wyomingensis* and *T. horridus* belong to *Marginocephalia* that is indicated on Figure 4 of Madzia, Boyd & Mazuch (2018), while *S. stenops* is nested within *Thyreophora* (*e.g*., Maidment et al., 2020).

### *Huayangosauridae* Dong, Tang & Zhou, 1982 (converted clade name)

**Registration number:** 623

**Definition.** The largest clade containing *Huayangosaurus taibaii* Dong, Tang & Zhou, 1982 but not *Stegosaurus stenops* Marsh, 1887. This is a maximum-clade definition. Abbreviated definition: max ∇ (*Huayangosaurus taibaii* Dong, Tang & Zhou, 1982 ~ *Stegosaurus stenops* Marsh, 1887).

**Reference phylogeny.** Figure 12 of Maidment et al. (2020) is treated here as the primary reference phylogeny. Additional reference phylogenies include Figure 11 of Maidment et al. (2008) and Figure 1 of Raven & Maidment (2017).

**Composition.** Under the primary reference phylogeny, *Huayangosauridae* comprises *Chungkingosaurus jiangbeiensis* and *Huayangosaurus taibaii*.

**Synonyms.** No other taxon names are currently in use for the same or approximate clade.

**Comments.** The name *Huayangosauridae* was first (informally) defined by Galton & Upchurch (2004: 358) who used the maximum-clade definition and selected *Huayangosaurus* as the internal specifier and *Stegosaurus* as the external specifier. We formalize this definition.

### *Hypsilophodontia* Cooper, 1985 (converted clade name)

**Registration number:** 624

**Definition.** The smallest clade within *Ornithopoda* containing *Hypsilophodon foxii* Huxley, 1869 and *Tenontosaurus tilletti* Ostrom, 1970, provided that it does not include *Iguanodon bernissartensis* Boulenger in Beneden, 1881. This is a minimum-clade definition. Abbreviated definition: min ∇ ∈ *Ornithopoda* (*Hypsilophodon foxii* Huxley, 1869 & *Tenontosaurus tilletti* Ostrom, 1970 | ~ *Iguanodon bernissartensis* Boulenger in Beneden, 1881).

**Reference phylogeny.** Figure 50 of Norman (2015) is treated here as the primary reference phylogeny.

**Composition.** Under the primary reference phylogeny, *Hypsilophodontia* comprises *Hypsilophodon foxii*, *Tenontosaurus* spp., and members of the clade *Rhabdodontidae*. However, see ‘Comments’ below for discussion of potential alternative composition of *Clypeodonta*.

**Synonyms.** No other taxon names are currently in use for the same or approximate clade.

**Comments.** The name *Hypsilophodontia* was (informally) defined as pertaining to “*Hypsilophodon foxii*, *Tenontosaurus tilletti*, their most recent common ancestor, and all of its descendants” (Norman, 2015: 171). However, such definition does not reflect alternative topologies that do not support *Hypsilophodontia* as reconstructed by Norman (2015), making it applicable for markedly different contents (see, *e.g*., Madzia, Boyd & Mazuch, 2018: Fig. 4).

Here we define the name *Hypsilophodontia* using a similar minimum-clade definition as that of Norman (2015) but by including the part “within *Ornithopoda*” in the definition, and adding an external specifier, we restrict the use of *Hypsilophodontia* to the node only when *H. foxii* represents an ornithopod (see Article 11.14 of the *ICPN*) and when *Hypsilophodon foxii* and *Tenontosaurus tilletti* are more closely related to each other than either is to *I. bernissartensis*, following the original intent of Norman (2015). Note that the internal specifier *Tenontosaurus tilletti* is not indicated in the primary reference phylogeny. The taxon is the type species of *Tenontosaurus* Ostrom, 1970 and is comprised there within the ‘tenontosaurs’.

### *Hypsilophodontidae* Dollo, 1882 (converted clade name)

**Registration number:** 625

**Definition.** The largest clade containing *Hypsilophodon foxii* Huxley, 1869 but not *Iguanodon bernissartensis* Boulenger in Beneden, 1881 and *Rhabdodon priscus* Matheron, 1869. This is a maximum-clade definition. Abbreviated definition: max ∇ (*Hypsilophodon foxii* Huxley, 1869 ~ *Iguanodon bernissartensis* Boulenger in Beneden, 1881 & *Rhabdodon priscus* Matheron, 1869).

**Reference phylogeny.** Figure 2 of Dieudonné et al. (2020) is treated here as the primary reference phylogeny.

**Composition.** Under the primary reference phylogeny, *Hypsilophodontidae* comprises *Hypsilophodon foxii*, *Gasparinisaura cincosaltensis*, and *Parksosaurus warreni*.

**Synonyms.** The name *Parksosaurinae* has been recently for the same contents (Yang et al., 2020), and attributed (apparently following the Principle of Coordination) to Buchholz (2002). No other taxon names are currently in use for the same or approximate clade.

**Comments.** *Hypsilophodontidae* was first (informally) defined by Sereno (1998: 61) who used the maximum-clade definition and *Hypsilophodon foxii* as the internal specifier and *Parasaurolophus walkeri* as the external specifier. Here we use the same type of definition but replace *P. walkeri* with *I. bernissartensis*. This taxon has always been considered outside *Hypsilophodontidae*. Additionally, we include *Rhabdodon priscus* as a second external specifier to prevent the inclusion of *Rhabdodontidae* within *Hypsilophodontidae* under the topology of Norman (2015: Fig. 50).

### *Iguanodontia* Baur, 1891 (converted clade name)

**Registration number:** 626

**Definition.** The smallest clade containing *Dryosaurus altus* (Marsh, 1878), *Iguanodon bernissartensis* Boulenger in Beneden, 1881, *Rhabdodon priscus* Matheron, 1869, and *Tenontosaurus tilletti* Ostrom, 1970, provided that it does not include *Hypsilophodon foxii* Huxley, 1869. This is a minimum-clade definition. Abbreviated definition: min ∇ (*Dryosaurus altus* (Marsh, 1878) & *Iguanodon bernissartensis* Boulenger in Beneden, 1881 & *Rhabdodon priscus* Matheron, 1869 & *Tenontosaurus tilletti* Ostrom, 1970 | ~ *Hypsilophodon foxii* Huxley, 1869).

**Reference phylogeny.** Figure 12 of Madzia, Jagt & Mulder (2020) is treated here as the primary reference phylogeny. Additional reference phylogenies include Figure 16 of Han et al. (2018), Figure 20 of Verdú et al. (2018), Figure 25 of Herne et al. (2019), and Figure 9 of Verdú et al. (2020).

**Composition.** Under the primary reference phylogeny, *Iguanodontia* comprises members of the clade *Rhabdodontomorpha*, *Tenontosaurus* spp., and *Dryomorpha*.

**Synonyms.** No other taxon names are currently in use for the same or approximate clade. *Clypeodonta*, as reconstructed by Norman (2015) covers a similar taxic composition; though the topology of Norman (2015) differs from that of the primary phylogeny of *Iguanodontia* significantly.

**Comments.** The application of *Iguanodontia* has been described and discussed by Madzia, Boyd & Mazuch (2018: Appendix 1) and Madzia, Jagt & Mulder (2020: Table 1). We therefore refer to these studies for details. Our definition differs from that advocated by Madzia, Boyd & Mazuch (2018) and Madzia, Jagt & Mulder (2020) in that the name is newly applicable only if it is used for a clade that does not include *Hypsilophodon foxii* (*e.g*., it becomes inapplicable under the topology of Norman, 2015: Fig. 50).

### *Iguanodontidae* Bonaparte, 1850 (converted clade name)

**Registration number:** 627

**Definition.** The largest clade containing *Iguanodon bernissartensis* Boulenger in Beneden, 1881 but not *Hadrosaurus foulkii* Leidy, 1858. This is a maximum-clade definition. Abbreviated definition: max ∇ (*Iguanodon bernissartensis* Boulenger in Beneden, 1881 ~ *Hadrosaurus foulkii* Leidy, 1858).

**Reference phylogeny.** Figure 13 of Madzia, Jagt & Mulder (2020) is treated here as the primary reference phylogeny. Additional reference phylogenies include Figure 3 of Madzia, Boyd & Mazuch (2018), Figure 20 of Verdú et al. (2018), Figure 32 of Tsogtbaatar et al. (2019), Figure 7 of Kobayashi et al. (2021), and Figure 11 of Santos-Cubedo et al. (2021).

**Composition.** Under the primary reference phylogeny, *Iguanodontidae* comprises *Barilium dawsoni*, *Iguanodon bernissartensis*, *Iguanodon galvensis*, and *Lurdusaurus arenatus*.

**Synonyms.** The name *Iguanodontoidea* Hay, 1902 is an approximate synonym of *Iguanodontidae* (see, *e.g*., Figure 20 of Verdú et al., 2018). Both these names have been used for various sets of taxa thought or reconstructed to be more closely related to *Iguanodon bernissartensis* than to hadrosaurids. Considering that significant differences exist between phylogeny reconstructions of *Iguanodon*-grade ornithopods (*e.g*., Madzia, Boyd & Mazuch, 2018; Verdú et al., 2018; Madzia, Jagt & Mulder, 2020; McDonald et al., 2021), it is difficult to link either of the names to a certain, stable composition. Here, we prefer to apply the name *Iguanodontidae* because it is more frequent in the literature and because it was coined 52 years before *Iguanodontoidea*. It is worth noting that the name *Iguanodontoidea* has been also used as an approximate synonym of *Hadrosauriformes* (see the name entry).

**Comments.** The name *Iguanodontidae* was first (informally) defined before (Sereno, 1998; Sereno, 2005; Santos-Cubedo et al., 2021). These definitions were maximum-clade and used *Iguanodon bernissartensis* as the internal specifier and *Parasaurolophus walkeri* (Sereno, 1998; Sereno, 2005) or *Corythosaurus casuarius* (Santos-Cubedo et al., 2021) as the external specifier. We apply a similar definition but replace *P. walkeri*/*Corythosaurus casuarius* with *H. foulkii*. Note that even though the study of Santos-Cubedo et al. (2021) appeared after the publication of *Phylonyms* (de Queiroz, Cantino & Gauthier, 2020), the work does not meet the general requirements for establishing *Iguanodontidae* as a phylogenetically defined clade name (see Articles 7 of the *ICPN*), nor it provides anything that would indicate such intention. Specifically, the name *Iguanodontidae* is not explicitly designated as a converted clade name, no bibliographic citations demonstrating prior application of the name to a taxon approximating the clade for which it is being established have been provided (including the authorship of the preexisting name), and no evidence is provided that the required information has been submitted to the registration database for phylogenetically defined names, the *RegNum* (registration number is missing). The study specifies the phylogenetic information, such as the placement of the clade on the ornithopod tree and the distribution of apomorphies supporting the existence of the clade, and presents the hypothesized composition of the clade. This information alone, however, would not be sufficient for the name *Iguanodontidae* to be established as a converted clade name, as required by the *ICPN*.

### *Jeholosauridae* Han et al., 2012 (converted clade name)

**Registration number:** 628

**Definition.** The largest clade outside *Hypsilophodontidae* or *Thescelosauridae* containing *Jeholosaurus shangyuanensis* Xu, Wang & You, 2000 but not *Hypsilophodon foxii* Huxley, 1869, *Iguanodon bernissartensis* Boulenger in Beneden, 1881, *Pachycephalosaurus wyomingensis* (Gilmore, 1931), *Thescelosaurus neglectus* Gilmore, 1913, and *Triceratops horridus* Marsh, 1889. This is a maximum-clade definition. Abbreviated definition: max ∇ ∉ *Hypsilophodontidae* ∨ *Thescelosauridae* (*Jeholosaurus shangyuanensis* Xu, Wang & You 2000 ~ *Hypsilophodon foxii* Huxley, 1869 & *Iguanodon bernissartensis* Boulenger in Beneden, 1881 & *Pachycephalosaurus wyomingensis* (Gilmore, 1931) & *Thescelosaurus neglectus* Gilmore, 1913 & *Triceratops horridus* Marsh, 1889).

**Reference phylogeny.** Figure 25 of Herne et al. (2019) is treated here as the primary reference phylogeny. Additional reference phylogenies include Figure 16 of Han et al. (2018), Figure 4 of Madzia, Boyd & Mazuch (2018), and Figure 57 of Barta & Norell (2021).

**Composition.** Under the primary reference phylogeny, *Jeholosauridae* comprises *Changchunsaurus parvus*, *Haya griva*, and *Jeholosaurus shangyuanensis*. Under alternative hypotheses, however, *Jeholosauridae* includes *Jeholosaurus shangyuanensis* and *Yueosaurus tiantaiensis* (*e.g*., Madzia, Boyd & Mazuch, 2018: Fig. 4; Barta & Norell, 2021: Fig. 57).

**Synonyms.** The name *Jeholosaurinae* has been used recently for the same contents (Yang et al., 2020), and attributed (apparently following the Principle of Coordination) to Han et al. (2012). No other taxon names are currently in use for the same or approximate clade.

**Comments.** We use a maximum-clade definition similar to that of Han et al. (2012), which is the only definition (informally) used for *Jeholosauridae*. Our definition differs in that we replaced the original representative of *Ceratopsia* (*Protoceratops andrewsi*) with a taxon that is widely used in phylogenetic definitions of ornithischian clade names (*Triceratops horridus*). Additionally, our definition prevents the use of *Jeholosauridae* under the potential hypotheses in which *Jeholosaurus* is inferred as part of *Hypsilophodontidae* or *Thescelosauridae*. Note that the internal specifiers *Pachycephalosaurus wyomingensis* and *Triceratops horridus* are not included in the primary reference phylogeny. The former belongs to *Pachycephalosauria* (see, *e.g*., Dieudonné et al., 2020), while the latter is part of *Ceratopsia* (*e.g*., Morschhauser et al., 2019), both within *Marginocephalia* that is indicated on Figure 25 of Herne et al. (2019).

### *Kritosaurini* Glut, 1997 (converted clade name)

**Registration number:** 629

**Definition.** The largest clade containing *Kritosaurus navajovius* Brown, 1910 but not *Brachylophosaurus canadensis* Sternberg, 1953, *Edmontosaurus regalis* Lambe, 1917, *Hadrosaurus foulkii* Leidy, 1858, and *Saurolophus osborni* Brown, 1912. This is a maximum-clade definition. Abbreviated definition: max ∇ (*Kritosaurus navajovius* Brown, 1910 ~ *Brachylophosaurus canadensis* Sternberg, 1953 & *Edmontosaurus regalis* Lambe, 1917 & *Hadrosaurus foulkii* Leidy, 1858 & *Saurolophus osborni* Brown, 1912).

**Reference phylogeny.** Figure 18 of Prieto-Márquez, Wagner & Lehman (2020) is treated here as the primary reference phylogeny. Additional reference phylogenies include Figure 5 of Kobayashi et al. (2019), Figure 11 of Prieto-Márquez et al. (2019), Figure 9 of Zhang et al. (2019), Figure 5 of Zhang et al. (2020), Figure 7 of Kobayashi et al. (2021), and Figure 10 of Longrich et al. (2021).

**Composition.** Under the primary reference phylogeny *Kritosaurini* comprises *Gryposaurus* spp., *Kritosaurus* spp., *Rhinorex condrupus*, *Secernosaurus koerneri*, and the specimen ‘Big Bend UTEP 37.7’.

**Synonyms.** No other taxon names are currently in use for the same or approximate clade.

**Comments.** The study of Lapparent & Lavocat (1955) has been cited to be the reference establishing the name *Kritosaurini* (*e.g*., Prieto-Márquez, 2014). However, Lapparent & Lavocat (1955) used ‘Kritosaurinés’ rather than ‘Kritosaurini’. The name *Kritosaurini* was then used by Brett-Surman (1989) and by Glut (1997). Since Brett-Surman (1989) is an unpublished doctoral dissertation, we consider Glut (1997) to be the earliest publication to spell the name *Kritosaurini*. The name was first (informally) defined by Prieto-Márquez (2014) who applied the minimum-clade definition and used *Kritosaurus navajovius*, *Gryposaurus notabilis*, and *Naashoibitosaurus ostromi* as the internal specifiers. We preserve the original intent of Prieto-Márquez (2014) but prefer to apply the maximum-clade definition. *Kritosaurus navajovius* is used as the internal specifier and *Hadrosaurus foulkii*, and representatives of *Brachylophosaurini* (*Brachylophosaurus canadensis*), *Edmontosaurini* (*Edmontosaurus regalis*), and *Saurolophini* (*Saurolophus osborni*), as the external specifiers.

### *Lambeosaurinae* Parks, 1923 (converted clade name)

**Registration number:** 630

**Definition.** The largest clade containing *Lambeosaurus lambei* Parks, 1923 but not *Hadrosaurus foulkii* Leidy, 1858 and *Saurolophus osborni* Brown, 1912. This is a maximum-clade definition. Abbreviated definition: max ∇ (*Lambeosaurus lambei* Parks, 1923 ~ *Hadrosaurus foulkii* Leidy, 1858 & *Saurolophus osborni* Brown, 1912).

**Reference phylogeny.** Figure 18 of Prieto-Márquez, Wagner & Lehman (2020) is treated here as the primary reference phylogeny. Additional reference phylogenies include Figure 5 of Kobayashi et al. (2019), Figure 11 of Prieto-Márquez et al. (2019), Figure 9 of Zhang et al. (2019), Figure 5 of Zhang et al. (2020), Figure 7 of Kobayashi et al. (2021), and Figure 10 of Longrich et al. (2021).

**Composition.** Under the primary reference phylogeny, *Lambeosaurinae* comprises *Aralosaurus tuberiferus*, *Canardia garonnensis*, *Jaxartosaurus aralensis*, and members of the clades *Corythosauria* and *Tsintaosaurini*.

**Synonyms.** No other taxon names are currently in use for the same or approximate clade.

**Comments.** The name *Lambeosaurinae* has been (informally) defined before (Sereno, 1998; Sereno, 2005; Prieto-Márquez, 2010). These definitions were maximum-clade and used *Parasaurolophus* (Sereno, 1998) or *Lambeosaurus lambei* (Prieto-Márquez, 2010) as the internal specifiers and *Saurolophus* (Sereno, 1998) or *Hadrosaurus foulkii*, *Saurolophus osborni*, and *Edmontosaurus regalis* (Prieto-Márquez, 2010) as the external specifiers. Sereno (2005), apparently erroneously, defined *Lambeosaurinae* as pertaining to “(t)he most inclusive taxon containing *Saurolophus osborni* Brown, 1912 but not *Parasaurolophus walkeri* Parks, 1922 and including *Lambeosaurus lambei* Parks, 1923”. Our formal maximum-clade definition is similar to that of Prieto-Márquez (2010) though we have removed *E. regalis* from the external specifiers because the taxon is consistently inferred outside *Lambeosaurinae* (Kobayashi et al., 2019; Prieto-Márquez et al., 2019; Prieto-Márquez, Wagner & Lehman, 2020; Zhang et al., 2019; Zhang et al., 2020; Gates, Evans & Sertich, 2021; Kobayashi et al., 2021; Longrich et al., 2021; Ramírez-Velasco et al., 2021).

### *Lambeosaurini* Sullivan et al., 2011 (converted clade name)

**Registration number:** 631

**Definition.** The largest clade containing *Lambeosaurus lambei* Parks, 1923 but not *Aralosaurus tuberiferus* Rozhdestvensky, 1968, *Parasaurolophus walkeri* Parks, 1922, and *Tsintaosaurus spinorhinus* Young, 1958. This is a maximum-clade definition. Abbreviated definition: max ∇ (*Lambeosaurus lambei* Parks, 1923 ~ *Aralosaurus tuberiferus* Rozhdestvensky, 1968 & *Parasaurolophus walkeri* Parks, 1922 & *Tsintaosaurus spinorhinus* Young, 1958).

**Reference phylogeny.** Figure 18 of Prieto-Márquez, Wagner & Lehman (2020) is treated here as the primary reference phylogeny. Additional reference phylogenies include Figure 5 of Kobayashi et al. (2019), Figure 11 of Prieto-Márquez et al. (2019), Figure 9 of Zhang et al. (2019), Figure 5 of Zhang et al. (2020), Figure 7 of Kobayashi et al. (2021), and Figure 10 of Longrich et al. (2021).

**Composition.** Under the primary reference phylogeny, *Lambeosaurini* comprises *Amurosaurus riabinini*, *Arenysaurus ardevoli*, *Blasisaurus canudoi*, *Corythosaurus* spp., *Hypacrosaurus stebingeri*, *Hypacrosaurus altispinus*, *Lambeosaurus* spp., *Magnapaulia laticaudus*, *Olorotitan arharensis* (misspelled as ‘*ararhensis’* in the primary reference phylogeny), *Sahaliyania elunchunorum*, and *Velafrons coahuilensis*.

**Synonyms.** The name *Corythosaurini* Glut, 1997 is an approximate synonym of *Lambeosaurini* (*e.g*., Evans & Reisz, 2007; Gates et al., 2007; Pereda-Suberbiola et al., 2009). However, its use has been discouraged (Prieto-Márquez et al., 2013) and all recent phylogenetic studies preferred to use *Lambeosaurini* instead (*e.g*., Xing, Mallon & Currie, 2017; Kobayashi et al., 2019; Prieto-Márquez et al., 2019; Zhang et al., 2020; Kobayashi et al., 2021; Longrich et al., 2021; Ramírez-Velasco et al., 2021). No other taxon names are currently in use for the same or approximate clade.

**Comments.** Even though Sullivan et al. (2011) did not explicitly formulate the definition of their newly proposed name *Lambeosaurini*, they noted that their “definition of the Lambeosaurini would be equivalent to node 38 of Prieto-Márquez (2010: fig. 9)” (Sullivan et al., 2011: 417). The name *Lambeosaurini* was first (informally) defined by Prieto-Márquez et al. (2013) who applied the maximum-clade definition and used *Lambeosaurus lambei* as the internal specifier and *Parasaurolophus walkeri*, *Tsintaosaurus spinorhinus*, and *Aralosaurus tuberiferus* as the external specifier. Such defined, the use of *Lambeosaurini* adheres to the original intent of Sullivan et al. (2011). We formalize this definition.

### *Leptoceratopsidae* Nopcsa, 1923 (converted clade name)

**Registration number:** 632

**Definition.** The largest clade containing *Leptoceratops gracilis* Brown, 1914b but not *Protoceratops andrewsi* Granger & Gregory, 1923 and *Triceratops horridus* Marsh, 1889. This is a maximum-clade definition. Abbreviated definition: max ∇ (*Leptoceratops gracilis* Brown, 1914b ~ *Protoceratops andrewsi* Granger & Gregory, 1923 & *Triceratops horridus* Marsh, 1889).

**Reference phylogeny.** Figure 10 of Morschhauser et al. (2019) is treated here as the primary reference phylogeny. Additional reference phylogenies include Figure S1 of Knapp et al. (2018), Figure 8A of Arbour & Evans (2019), Figure 3 of Yu et al. (2020), and Figure 4 of Yu et al. (2020).

**Composition.** Under the primary reference phylogeny, *Leptoceratopsidae* comprises *Cerasinops hodgskissi*, *Gryphoceratops morrisoni*, *Helioceratops brachygnathus*, *Ischioceratops zhuchengensis*, *Koreaceratops hwaseongensis*, *Leptoceratops gracilis*, *Montanoceratops cerorhynchus*, *Prenoceratops pieganensis*, *Udanoceratops tchizhovi*, *Unescoceratops koppelhusae*, and *Zhuchengceratops inexpectus*.

**Synonyms.** No other taxon names are currently in use for the same or approximate clade.

**Comments.** The name *Leptoceratopsidae* has been (informally) defined before by Makovicky (2001) who used *Leptoceratops gracilis* as the internal specifier and *Triceratops horridus* as the external specifier. Since *Leptoceratopsidae* has never been proposed an alternative use, we formalize a similar definition that differs only in adding *Protoceratops andrewsi* as a second external specifier.

### *Marginocephalia* Sereno, 1986 (converted clade name)

**Registration number:** 633

**Definition.** The smallest clade containing *Ceratops montanus* Marsh, 1888, *Pachycephalosaurus wyomingensis* (Gilmore, 1931), and *Triceratops horridus* Marsh, 1889. This is a minimum-clade definition. Abbreviated definition: min ∇ (*Ceratops montanus* Marsh, 1888 & *Pachycephalosaurus wyomingensis* (Gilmore, 1931) & *Triceratops horridus* Marsh, 1889).

**Reference phylogeny.** Figure 16 of Han et al. (2018) is treated here as the primary reference phylogeny. Additional reference phylogenies include Figure 4 of Madzia, Boyd & Mazuch (2018), Figure 25 of Herne et al. (2019), Figure 1 of Dieudonné et al. (2020), Figure 12 of Yang et al. (2020), and Figure 57 of Barta & Norell (2021).

**Composition.** Under the primary reference phylogeny, *Marginocephalia* comprises members of the clades *Ceratopsia* and *Pachycephalosauria*.

**Synonyms.** No other taxon names are currently in use for the same or approximate clade.

**Comments.** The name *Marginocephalia* has been (informally) defined before (Currie & Padian, 1997; Sereno, 1998; Sereno, 2005; Madzia, Boyd & Mazuch, 2018; Herne et al., 2019). These definitions, except for that of Herne et al. (2019), were minimum-clade and used *Ceratopsia* and *Pachycephalosauria* (Currie & Padian, 1997) or *Triceratops horridus* and *Pachycephalosaurus wyomingensis* (Sereno, 1998; Sereno, 2005; Madzia, Boyd & Mazuch, 2018) as the internal specifiers. Madzia, Boyd & Mazuch (2018) further included *Ceratops montanus* as a third internal specifier, stating that “(t)he first definition of Marginocephalia was node-based and used ‘Ceratopsia’ and ‘Pachycephalosauria’ as the internal specifiers […]. To follow the definition, and adhere to the ICPN (Art. 11), we have to use name-bearing species or their type specimens as specifiers which makes the name to be anchored on the types of *Ceratops montanus* and *Pachycephalosaurus wyomingensis*. Even if *C. montanus* may be a *nomen dubium*, its type specimen is unequivocally nested deeply within Ceratopsia and thus its use does not change the extent of the name” (Madzia, Boyd & Mazuch, 2018: Appendix 1). In turn, Herne et al. (2019) preferred a maximum-clade definition with *T. horridus* and *P. wyomingensis* as the internal specifiers and *Parasaurolophus walkeri* as the external specifier, arguing that “(previous) definitions (were) not complementary with present definitions of Cerapoda and Ornithopoda within a node-stem triplet arrangement of clades” and that “re-definition of Marginocephalia as a stem now mirrors its sister stem clade, Ornithopoda, within a node-based Cerapoda. As a result, this stabilization of definition allows for the definitive assignment of all cerapodan OTUs either as ornithopods or marginocephalians” (Herne et al., 2019: Supplemental Text S1: 4). However, *Marginocephalia* has never formed such ‘triplet’. When its use in a ‘node-branch triplet’ is considered, it is more closely tied with *Ceratopsia* and *Pachycephalosauria* rather than with *Cerapoda* and *Ornithopoda*. Here, the internal specifiers in the definition of *Marginocephalia* are used from among the taxa representing the two major subclades – *Ceratopsia* (*Ceratops montanus* and *Triceratops horridus*) and *Pachycephalosauria* (*Pachycephalosaurus wyomingensis*). Note that none of the internal specifiers is included in the primary reference phylogeny. *Ceratops montanus* and *Pachycephalosaurus wyomingensis* are name-bearers of *Ceratopsia* and *Pachycephalosauria*, respectively, and are deeply nested within these clades (*e.g*., Mallon et al., 2016; Dieudonné et al., 2020). *Triceratops horridus* is a late-diverging member of *Chasmosaurinae* within *Ceratopsia* (*e.g*., Morschhauser et al., 2019; Fowler & Freedman Fowler, 2020).

### *Nasutoceratopsini* Ryan et al., 2017 (converted clade name)

**Registration number:** 689

**Definition.** The largest clade containing *Nasutoceratops titusi* Sampson et al., 2013 but not *Centrosaurus apertus* Lambe, 1905. This is a maximum-clade definition. Abbreviated definition: max ∇ (*Nasutoceratops titusi* Sampson et al., 2013 ~ *Centrosaurus apertus* Lambe, 1905).

**Reference phylogeny.** Figure 9 of Chiba et al. (2018) is treated here as the primary reference phylogeny. Additional reference phylogenies include Figure 7 of Fiorillo & Tykoski (2012), Figure 10 of Ryan et al. (2017), and Figure 13 of Dalman et al. (2018).

**Composition.** Under the primary reference phylogeny, *Nasutoceratopsini* comprises *Avaceratops lammersi*, *Nasutoceratops titusi*, and the specimens CMN 8804, MOR 692, and the ‘Malta New Taxon’ (GPDM 63). Under an alternative hypothesis, however, *Nasutoceratopsini* includes only a single unequivocal member, *Nasutoceratops titusi* (Dalman et al., 2021: Fig. 23).

**Synonyms.** No other taxon names are currently in use for the same or approximate clade.

**Comments.** The name was first (informally) defined by Ryan et al. (2017) who applied the maximum-clade definition and used *Nasutoceratops titusi* as the internal specifier and *Centrosaurus apertus* as the external specifier. We formalize this definition.

### *Neoceratopsia* Sereno, 1986 (converted clade name)

**Registration number:** 634

**Definition.** The largest clade containing *Triceratops horridus* Marsh, 1889 but not *Chaoyangsaurus youngi* Zhao, Cheng & Xu, 1999 and *Psittacosaurus mongoliensis* Osborn, 1923. This is a maximum-clade definition. Abbreviated definition: max ∇ (*Triceratops horridus* Marsh, 1889 ~ *Chaoyangsaurus youngi* Zhao, Cheng & Xu, 1999 & *Psittacosaurus mongoliensis* Osborn, 1923).

**Reference phylogeny.** Figure 10 of Morschhauser et al. (2019) is treated here as the primary reference phylogeny. Additional reference phylogenies include Figure 16 of Han et al. (2018), Figure S1 of Knapp et al. (2018), and Figure 4 of Yu et al. (2020).

**Composition.** Under the primary reference phylogeny, *Neoceratopsia* comprises *Aquilops americanus*, *Archaeoceratops oshimai*, *Asiaceratops salsopaludalis*, *Auroraceratops rugosus*, ZPAL MgD-I/156 (= *Graciliceratops mongoliensis*), *Liaoceratops yanzigouensis*, *Mosaiceratops azumai*, *Stenopelix valdensis*, *Yamaceratops dorngobiensis*, and members of the clade *Euceratopsia*.

**Synonyms.** No other taxon names are currently in use for the same or approximate clade.

**Comments.** The name *Neoceratopsia* has been (informally) defined before by Sereno (1998, 2005) who applied a maximum-clade definition and used *Triceratops horridus* as the internal specifier and *Psittacosaurus mongoliensis* as the external specifier. We further include a second external specifier, *Chaoyangsaurus youngi*, to ensure that *Chaoyangsauridae*, a clade usually reconstructed as some of the earliest-diverging ceratopsians (*e.g*., Han et al., 2018; Knapp et al., 2018; Yu et al., 2020), are maintained outside *Neoceratopsia*.

### *Neoiguanodontia* Norman, 2014 (converted clade name)

**Registration number:** 635

**Definition.** The smallest clade containing *Hypselospinus fittoni* (Lydekker, 1889), *Iguanodon bernissartensis* Boulenger in Beneden, 1881, and *Parasaurolophus walkeri* Parks, 1922. This is a minimum-clade definition. Abbreviated definition: min ∇ (*Hypselospinus fittoni* (Lydekker, 1889) & *Iguanodon bernissartensis* Boulenger in Beneden, 1881 & *Parasaurolophus walkeri* Parks, 1922).

**Reference phylogeny.** Figure 2.26 of Norman (2014) is treated here as the primary reference phylogeny. Additional reference phylogenies include Figure 50 of Norman (2015), Figure 3 of Párraga & Prieto-Márquez (2019), and Figure 11 of McDonald et al. (2021).

**Composition.** Under the primary reference phylogeny, *Neoiguanodontia* comprises *Hypselospinus fittoni* and members of the clade *Hadrosauriformes*.

**Synonyms.** *Neoiguanodontia* is a potential heterodefinitional synonym of *Hadrosauriformes*. If *Hypselospinus fittoni* nests within the smallest clade containing *Hadrosaurus foulkii* and *Iguanodon bernissartensis* (*e.g*., Verdú et al., 2018; Santos-Cubedo et al., 2021: Fig. 11), the name *Hadrosauriformes* should have priority.

**Comments.** The application of *Neoiguanodontia* has been described and discussed by Madzia, Jagt & Mulder (2020: Table 1). We therefore refer to that study for details.

### *Neornithischia* Cooper, 1985 (converted clade name)

**Registration number:** 636

**Definition.** The largest clade containing *Iguanodon bernissartensis* Boulenger in Beneden, 1881 and *Triceratops horridus* Marsh, 1889 but not *Ankylosaurus magniventris* Brown, 1908 and *Stegosaurus stenops* Marsh, 1887. This is a maximum-clade definition. Abbreviated definition: max ∇ (*Iguanodon bernissartensis* Boulenger in Beneden, 1881 & *Triceratops horridus* Marsh, 1889 ~ *Ankylosaurus magniventris* Brown, 1908 & *Stegosaurus stenops* Marsh, 1887).

**Reference phylogeny.** Figure 4 of Madzia, Boyd & Mazuch (2018) is treated here as the primary reference phylogeny. Additional reference phylogenies include Figure 16 of Han et al. (2018), Figure 25 of Herne et al. (2019), Figure 1 of Dieudonné et al. (2020), and Figure 57 of Barta & Norell (2021).

**Composition.** Under the primary reference phylogeny, *Neornithischia* comprises *Agilisaurus louderbacki*, *Hexinlusaurus multidens*, *Hypsilophodon foxii*, *Kulindadromeus zabaikalicus*, *Leaellynasaura amicagraphica*, *Lesothosaurus diagnosticus*, *Othnielosaurus consors* (= *Nanosaurus agilis*; see Carpenter & Galton, 2018), *Yandusaurus hongheensis*, and members of the clades *Cerapoda*, *Jeholosauridae*, and *Thescelosauridae*.

**Synonyms.** No other taxon names are currently in use for the same or approximate clade.

**Comments.** The name *Neornithischia* has been (informally) defined before (Sereno, 1998; Sereno, 2005; Butler, Upchurch & Norman, 2008; Herne et al., 2019). These definitions were maximum-clade and used *Triceratops horridus* (Sereno, 1998), *Parasaurolophus walkeri* (Butler, Upchurch & Norman, 2008) or both, *T. horridus* and *P. walkeri* (Sereno, 2005; Herne et al., 2019) as the internal specifiers, and *Ankylosaurus magniventris* (Sereno, 1998; Sereno, 2005; Herne et al., 2019) or *A. magniventris* and *Stegosaurus stenops* (Butler, Upchurch & Norman, 2008) as the external specifiers. In order to maintain the ‘traditional’ concept of *Genasauria* as a clade comprising *Neornithischia* and *Thyreophora*, the internal specifiers in the definition of *Neornithischia* are used from among the taxa representing the two major subclades – *Ornithopoda* (*Iguanodon bernissartensis*) and *Marginocephalia* (*Triceratops horridus*) – and the external specifiers are used from among the taxa representing the thyreophoran clades *Ankylosauria* (*Ankylosaurus magniventris*) and *Stegosauria* (*Stegosaurus stenops*). Note that the internal specifier *Triceratops horridus* and the external specifiers *Ankylosaurus magniventris* and *Stegosaurus stenops* are not included in the primary reference phylogeny. *T. horridus* belongs to *Ceratopsia* (see, *e.g*., Morschhauser et al., 2019), while *Ankylosaurus magniventris* and *Stegosaurus stenops* are deeply nested members of *Thyreophora* (*e.g*., Thompson et al., 2012; Maidment et al., 2020), a clade that is indicated on Figure 4 of Madzia, Boyd & Mazuch (2018).

### *Nodosauridae* Marsh, 1890 (converted clade name)

**Registration number:** 637

**Definition.** The largest clade containing *Nodosaurus textilis* Marsh, 1889 but not *Ankylosaurus magniventris* Brown, 1908. This is a maximum-clade definition. Abbreviated definition: max ∇ (*Nodosaurus textilis* Marsh, 1889 ~ *Ankylosaurus magniventris* Brown, 1908).

**Reference phylogeny.** Figure 5 of Rivera-Sylva et al. (2018a) is treated here as the primary reference phylogeny. Additional reference phylogenies include Figure 3 of Thompson et al. (2012), Figure 11 of Arbour & Currie (2016), Figure 1 of Arbour, Zanno & Gates (2016), and Figure 3 of Brown et al. (2017).

**Composition.** Under the primary reference phylogeny, *Nodosauridae* comprises *Dongyangopelta yangyanensis*, *Gastonia burgei*, *Gargoyleosaurus parkpinorum*, and members of the clades *Nodosaurinae* and *Polacanthinae*.

**Synonyms.** No other taxon names are currently in use for the same or approximate clade.

**Comments.** The name *Nodosauridae* has been (informally) defined before by Sereno (1998, 2005) who used *Panoplosaurus mirus* (Sereno, 1998) or *Panoplosaurus mirus* and *Nodosaurus textilis* Sereno (2005) as the internal specifiers and *Ankylosaurus magniventris* as the external specifier. Considering that all phylogeny reconstructions that include *P. mirus* and *N. textilis* indicate that these taxa are more closely related to each other than either is to *A. magniventris* (or placed outside the *Ankylosauridae* + *Nodosauridae* node), we use a definition that incorporates *Nodosaurus textilis* as the sole internal specifier.

### *Nodosaurinae* Abel, 1919 (converted clade name)

**Registration number:** 638

**Definition.** The largest clade containing *Nodosaurus textilis* Marsh, 1889, but not *Hylaeosaurus armatus* Mantell, 1833, *Mymoorapelta maysi* Kirkland & Carpenter, 1994, and *Polacanthus foxii* Owen in Anonymous, 1865. This is a maximum-clade definition. Abbreviated definition: max ∇ (*Nodosaurus textilis* Marsh, 1889 ~ *Hylaeosaurus armatus* Mantell, 1833 & *Mymoorapelta maysi* Kirkland & Carpenter, 1994 & *Polacanthus foxii* Owen in Anonymous, 1865).

**Reference phylogeny.** Figure 5 of Rivera-Sylva et al. (2018a) is treated here as the primary reference phylogeny. Additional reference phylogenies include Figure 3 of Thompson et al. (2012), Figure 11 of Arbour & Currie (2016), Figure 1 of Arbour, Zanno & Gates (2016), and Figure 3 of Brown et al. (2017).

**Composition.** Under the primary reference phylogeny, *Nodosaurinae* comprises *Acantholipan gonzalezi*, *Ahshislepelta minor*, *Niobrarasaurus coleii*, *Nodosaurus textilis*, *Peloroplites cedrimontanus*, *Sauropelta edwardsi*, *Silvisaurus condrayi*, *Taohelong jinchengensis*, *Tatankacephalus cooneyorum*, members of the clades *Panoplosaurini* and *Struthiosaurini*, and the specimen CPC 273.

**Synonyms.** No other taxon names are currently in use for the same or approximate clade.

**Comments.** The name *Nodosaurinae* has been (informally) defined before (Sereno, 1998; Sereno, 2005). Both these definitions were maximum-clade and used *Panoplosaurus* (Sereno, 1998) or *Panoplosaurus mirus* and *Nodosaurus textilis* (Sereno, 2005) as the internal specifiers and *Sarcolestes* and *Hylaeosaurus* (Sereno, 1998) or *Polacanthus foxii*, *Hylaeosaurus armatus*, and *Mymoorapelta maysi* as the external specifiers (Sereno, 2005). We formalize a definition similar to that of Sereno (2005) but use a single internal specifier.

### *Ornithischia* Seeley, 1888 (converted clade name)

**Registration number:** 639

**Definition.** The largest clade containing *Iguanodon bernissartensis* Boulenger in Beneden, 1881 but not *Allosaurus fragilis* Marsh, 1877a and *Camarasaurus supremus* Cope, 1877. This is a maximum-clade definition. Abbreviated definition: max ∇ (*Iguanodon bernissartensis* Boulenger in Beneden, 1881 ~ *Allosaurus fragilis* Marsh, 1877a & *Camarasaurus supremus* Cope, 1877).

**Reference phylogeny.** Figure 4 of Madzia, Boyd & Mazuch (2018) is treated here as the primary reference phylogeny. Additional reference phylogenies include Figure 2 of Boyd (2015), Figure 1 of Baron, Norman & Barrett (2017a), Figure 1 of Baron, Norman & Barrett (2017b), and Figure 1 of Langer et al. (2017).

**Composition.** Under the primary reference phylogeny, *Ornithischia* comprises *Pisanosaurus mertii* and members of the clades *Heterodontosauridae* and *Genasauria*. Note, however, that the early evolution and basal branching of *Ornithischia* is currently unsettled. For example, *P. mertii* may represent either an early-diverging ornithischian (recently, *e.g*., Desojo et al., 2020) or a (non-dinosaur) silesaurid dinosauriform (Agnolín & Rozadilla, 2018, Baron, 2019). The same may be true for members of *Silesauridae*, a group often reconstructed as the sister taxon to *Dinosauria* (*e.g*., Nesbitt et al., 2010; Peecook et al., 2013; Ezcurra, 2016; Cau, 2018; Ezcurra et al., 2020), that have recently been inferred to represent early-diverging representatives of *Ornithischia* (Langer & Ferigolo, 2013; Cabreira et al., 2016; Pacheco et al., 2019; Müller & Garcia, 2020).

**Synonyms.** No other taxon names are currently in use for the same or approximate clade. Though not in use, the name *Predentata* Marsh, 1894 is being occasionally recalled as an approximate synonym (*e.g*., Butler, Upchurch & Norman, 2008; Langer & Ferigolo, 2013).

**Comments.** The name *Ornithischia* has been (informally) defined before (Padian & May, 1993; Sereno, 1998; Weishampel, 2004; Norman, Witmer & Weishampel, 2004; Sereno, 2005; Baron, Norman & Barrett, 2017a). These definitions were maximum-clade and used *Triceratops horridus* (Padian & May, 1993; Sereno, 1998; Weishampel, 2004; Sereno, 2005; Baron, Norman & Barrett, 2017a) or *Iguanodon bernissartensis* (Norman, Witmer & Weishampel, 2004) as the internal specifiers. In turn, “birds” (Padian & May, 1993), *Neornithes* (Sereno, 1998), *Tyrannosaurus* (Weishampel, 2004), *Cetiosaurus* (Norman, Witmer & Weishampel, 2004), *Passer domesticus* and *Saltasaurus loricatus* (Sereno, 2005), and *Passer domesticus* and *Diplodocus carnegii* (Baron, Norman & Barrett, 2017a) were used as the external specifiers. Although both, *I. bernissartensis* and *T. horridus*, are clearly ‘traditional’ members of *Ornithischia*, we have selected the former as the internal specifier and *Allosaurus fragilis* and *Camarasaurus supremus* as the external specifiers. These specifiers are preferred because (a) they represent deeply nested taxa within their respective clades (*Ornithischia*, *Theropoda*, and *Sauropodomorpha*), (b) they have been historically associated with these clades, thus being their ‘traditional’ members, and (c) their phylogenetic placements are stable across studies. Two external specifiers, instead of one, are used due to the alternative topologies of dinosaur relationships (see, *e.g*., Baron, Norman & Barrett, 2017a; Langer et al., 2017). Additionally, *Iguanodon bernissartensis* was used as the internal specifier in the formal definition of *Dinosauria* (Langer et al., 2020), considered therein as a ‘traditional’ representative of *Ornithischia*, and the external specifier in the formal definition of *Sauropodomorpha* (Fabbri et al., 2020), again considered therein as a ‘traditional’ representative of *Ornithischia*; *A. fragilis* was used as the internal specifier in the formal definitions of *Theropoda* (Naish et al., 2020) and *Saurischia* (Gauthier et al., 2020), and as the external specifier in the formal definition of *Sauropodomorpha* (Fabbri et al., 2020), considered in these contributions as a ‘traditional’ representative of *Theropoda*; and *Camarasaurus supremus* was used as the internal specifier in the formal definition of *Saurischia* (Gauthier et al., 2020) and considered therein as a ‘traditional’ representative of *Sauropodomorpha*.

### *Ornithopoda* Marsh, 1881 (converted clade name)

**Registration number:** 640

**Definition.** The largest clade containing *Iguanodon bernissartensis* Boulenger in Beneden, 1881 but not *Pachycephalosaurus wyomingensis* (Gilmore, 1931) and *Triceratops horridus* Marsh, 1889. This is a maximum-clade definition. Abbreviated definition: max ∇ (*Iguanodon bernissartensis* Boulenger in Beneden, 1881 ~ *Pachycephalosaurus wyomingensis* (Gilmore, 1931) & *Triceratops horridus* Marsh, 1889).

**Reference phylogeny.** Figure 4 of Madzia, Boyd & Mazuch (2018) is treated here as the primary reference phylogeny. Additional reference phylogenies include Figure 16 of Han et al. (2018), Figure 25 of Herne et al. (2019), Figure 1 of Dieudonné et al. (2020), and Figure 57 of Barta & Norell (2021).

**Composition.** Under the primary reference phylogeny, *Ornithopoda* comprises *Burianosaurus augustai*, *Gideonmantellia amosanjuanae*, and members of the clades *Elasmaria* and *Iguanodontia*.

**Synonyms.** No other taxon names are currently in use for the same or approximate clade.

**Comments.** The name *Ornithopoda* has been (informally) defined before (Sereno, 1998; Norman et al., 2004; Sereno, 2005; Butler, Upchurch & Norman, 2008; Herne et al., 2019). Two of these definitions were minimum-clade (Sereno, 1998; Sereno, 2005) and used *Parasaurolophus walkeri* and *Heterodontosaurus tucki* as the internal specifiers. Sereno (2005) further restricted the name to a hypothesis in which *P. walkeri* and *H. tucki* were more closely related to each other than either was to *Pachycephalosaurus wyomingensis*, *Triceratops horridus*, and *Ankylosaurus magniventris*. In turn, Norman et al. (2004), Butler, Upchurch & Norman (2008), and Herne et al. (2019) defined *Ornithopoda* as pertaining to the largest clade containing *Edmontosaurus regalis* (in Norman et al., 2004) or *P. walkeri* (in Butler, Upchurch & Norman, 2008 and Herne et al., 2019) but not *T. horridus*. Herne et al. (2019) additionally included a second external specifier (*P. wyomingensis*). We selected a definition that follows Herne et al. (2019) in that it includes two external specifiers (*T. horridus* and *P. wyomingensis*, representatives of two clades closely related to ornithopods; *i.e*., *Ceratopsia* and *Pachycephalosauria*, respectively). However, we prefer to use *Iguanodon bernissartensis* as the internal specifier rather than *P. walkeri*, because the former is among the few taxa that have been considered a part of *Ornithopoda* when the name was being introduced in the literature (*e.g*., Marsh, 1882). The inclusion of a different internal specifier does not change the extent of *Ornithopoda* under any of the published phylogeny inferences. Note that the external specifiers *Pachycephalosaurus wyomingensis* and *Triceratops horridus* are not included in the primary reference phylogeny. The former belongs to *Pachycephalosauria* (see, *e.g*., Dieudonné et al., 2020), while the latter is part of *Ceratopsia* (*e.g*., Morschhauser et al., 2019), both within *Marginocephalia* that is indicated on Figure 4 of Madzia, Boyd & Mazuch (2018).

### *Orodrominae* Brown et al., 2013 (converted clade name)

**Registration number:** 641

**Definition.** The largest clade within *Hypsilophodontidae* ∨ *Thescelosauridae* containing *Orodromeus makelai* Horner & Weishampel, 1988 but not *Hypsilophodon foxii* Huxley, 1869 and *Thescelosaurus neglectus* Gilmore, 1913. This is a maximum-clade definition. Abbreviated definition: max ∇ ∈ *Hypsilophodontidae* ∨ *Thescelosauridae* (*Orodromeus makelai* Horner & Weishampel, 1988 ~ *Hypsilophodon foxii* Huxley, 1869 & *Thescelosaurus neglectus* Gilmore, 1913).

**Reference phylogeny.** Figure 4 of Madzia, Boyd & Mazuch (2018) is treated here as the primary reference phylogeny. Additional reference phylogenies include Figure 25 of Herne et al. (2019) and Figure 57 of Barta & Norell (2021).

**Composition.** Under the primary reference phylogeny, *Orodrominae* comprises *Albertadromeus syntarsus*, *Changchunsaurus parvus*, *Haya griva*, *Koreanosaurus boseongensis*, *Orodromeus makelai*, *Oryctodromeus cubicularis*, *Zephyrosaurus schaffi*, and the ‘Kaiparowits orodromine’.

**Synonyms.** No other taxon names are currently in use for the same or approximate clade.

**Comments.** The name *Orodrominae* has been (informally) defined before (Brown et al., 2013; Boyd, 2015). Both these definitions were maximum-clade and used *Orodromeus makelai* as the internal specifier and *Thescelosaurus neglectus* (Brown et al., 2013) or *Thescelosaurus neglectus* and *Parasaurolophus walkeri* (Boyd, 2015) as the external specifiers. Considering the ‘traditional concept’ of *Orodrominae*, as a subclade of *Thescelosauridae*/‘hypsilophodonts’, and keeping in mind the unstable phylogenetic position of *H. foxii* (*e.g*., Madzia, Boyd & Mazuch, 2018), we apply *Orodrominae* only when it is inferred either within *Thescelosauridae* or *Hypsilophodontidae* (see Article 11.14 of the *ICPN*).

### *Pachycephalosauria* Maryańska & Osmólska, 1974 (converted clade name)

**Registration number:** 642

**Definition.** The largest clade containing *Pachycephalosaurus wyomingensis* (Gilmore, 1931) but not *Ceratops montanus* Marsh, 1888 and *Triceratops horridus* Marsh, 1889. This is a maximum-clade definition. Abbreviated definition: max ∇ (*Pachycephalosaurus wyomingensis* (Gilmore, 1931) ~ *Ceratops montanus* Marsh, 1888 & *Triceratops horridus* Marsh, 1889).

**Reference phylogeny.** Figure 27 of Schott & Evans (2017) is treated here as the primary reference phylogeny. Additional reference phylogenies include Figure 5 of Evans et al. (2013), Figure 16 of Han et al. (2018), and Figure 1 of Dieudonné et al. (2020).

**Composition.** Under the primary reference phylogeny, *Pachycephalosauria* comprises *Wannanosaurus yanshiensis* and members of the clade *Pachycephalosauridae*.

**Synonyms.** No other taxon names are currently in use for the same or approximate clade.

**Comments.** The name *Pachycephalosauria* has been (informally) defined before (Sereno, 1998; Maryańska, Chapman & Weishampel, 2004; Sereno, 2005). These definitions were maximum-clade and used *Pachycephalosaurus* (Sereno, 1998) or *Pachycephalosaurus wyomingensis* (Maryańska, Chapman & Weishampel, 2004; Sereno, 2005) as the internal specifier and *Triceratops* (Sereno, 1998), *Triceratops horridus* (Maryańska, Chapman & Weishampel, 2004), or *Triceratops horridus*, *Heterodontosaurus tucki*, *Hypsilophodon foxii*, and *Ankylosaurus magniventris* (Sereno, 2005) as the external specifiers. Even though the position of *Hypsilophodon foxii* and *Heterodontosaurus tucki* is unstable across studies (*e.g*., see, *e.g*., Han et al., 2018; Madzia, Boyd & Mazuch, 2018; Herne et al., 2019; Dieudonné et al., 2020), and, for example, *Heterodontosauridae* were inferred to be more closely related to *P. wyomingensis* than to *T. horridus* (Dieudonné et al., 2020: Fig. 1), inclusion of these taxa among the external specifiers does not need to be necessary as it can be expected that *Pachycephalosauria*, as ‘traditionally’ defined, may cover taxa that are markedly different from the Late Cretaceous members of the clade. We use a definition similar to that of Maryańska, Chapman & Weishampel (2004) but include *Ceratops montanus* as a second external specifier. Note that none of the external specifiers is included in the primary reference phylogeny. Both, *C. montanus* and *T. horridus*, however, are members of *Ceratopsidae* within *Ceratopsia* (*e.g*., Mallon et al., 2016; Morschhauser et al., 2019).

### *Pachycephalosauridae* Sternberg, 1945 (converted clade name)

**Registration number:** 643

**Definition.** The smallest clade containing *Pachycephalosaurus wyomingensis* (Gilmore, 1931) and *Stegoceras validum* Lambe, 1902, provided that it does not include *Heterodontosaurus tucki* Crompton & Charig, 1962. This is a minimum-clade definition. Abbreviated definition: min ∇ (*Pachycephalosaurus wyomingensis* (Gilmore, 1931) & *Stegoceras validum* Lambe, 1902 | ~ *Heterodontosaurus tucki* Crompton & Charig, 1962).

**Reference phylogeny.** Figure 27 of Schott & Evans (2017) is treated here as the primary reference phylogeny. Additional reference phylogenies include Figure 5 of Evans et al. (2013), Figure 3 of Williamson & Brusatte (2016), and Figure 14 of Woodruff et al. (2021).

**Composition.** Under the primary reference phylogeny, *Pachycephalosauridae* comprises *Colepiocephale lambei*, *Hanssuesia sternbergi*, *Stegoceras* spp., and members of the clade *Pachycephalosaurinae*.

**Synonyms.** No other taxon names are currently in use for the same or approximate clade.

**Comments.** The name *Pachycephalosauridae* has been (informally) defined before by Sereno (1998, 2005) who applied the minimum-clade definition and used *Pachycephalosaurus wyomingensis* and *Stegoceras validum* as the internal specifiers. This definition is followed here though we also include a qualifying clause that excludes *H. tucki* from *Pachycephalosauridae*. Even though no phylogenetic analysis has ever reconstructed *H. tucki* or any other ‘traditional’ heterodontosaurid to be within the smallest clade containing *P. wyomingensis* and *S. validum*, *Heterodontosauridae* were inferred to be early-diverging pachycephalosaurs (Dieudonné et al., 2020). The addition of a qualifying clause that excludes *H. tucki* from *Pachycephalosauridae* will therefore ensure that the name will never comprise *Heterodontosauridae*. Note that *H. tucki* is not included in the primary reference phylogeny. See Figure 1 of Dieudonné et al. (2020) for its potential placement with respect to pachycephalosaurids.

### *Pachycephalosaurinae* Sereno, 1997 (converted clade name)

**Registration number:** 748

**Definition.** The largest clade containing *Pachycephalosaurus wyomingensis* (Gilmore, 1931) but not *Stegoceras validum* Lambe, 1902. This is a maximum-clade definition. Abbreviated definition: max ∇ (*Pachycephalosaurus wyomingensis* (Gilmore, 1931) ~ *Stegoceras validum* Lambe, 1902).

**Reference phylogeny.** Figure 27 of Schott & Evans (2017) is treated here as the primary reference phylogeny. Additional reference phylogenies include Figure 7 of Longrich, Sankey & Tanke (2010), Figure 5 of Evans et al. (2013), Figure 3 of Williamson & Brusatte (2016), and Figure 14 of Woodruff et al. (2021).

**Composition.** Under the primary reference phylogeny, *Pachycephalosaurinae* comprises *Acrotholus audeti*, *Amtocephale gobiensis*, *Foraminacephale brevis*, *Goyocephale lattimorei*, *Homalocephale calathocercos*, *Prenocephale prenes*, *Sphaerotholus* spp., *Tylocephale gilmorei*, and members of the clade *Pachycephalosaurini*.

**Synonyms.** No other taxon names are currently in use for the same or approximate clade.

**Comments.** The name *Pachycephalosaurinae* has been (informally) defined before (Sereno, 1998; Sullivan, 2003; Sereno, 2005). Both types of definitions, minimum-clade as well as maximum-clade, have been proposed for the name. Sereno (1998, 2005) preferred a maximum-clade definition and used *Pachycephalosaurus* (Sereno, 1998) or *Pachycephalosaurus wyomingensis* (Sereno, 2005) as the internal specifier and *Stegoceras* (Sereno, 1998) or *Stegoceras validum* (Sereno, 2005) as the external specifier, while Sullivan (2003) applied a minimum-clade definition, using *Colepiocephale*, *Prenocephale*, *Tylocephale*, *Hanssuesia*, and *Pachycephalosaurus* and *Stygimoloch* (*Pachycephalosaurini sensu* Sullivan, 2003), as the internal specifiers. We formalize the definition of Sereno (2005).

### *Pachycephalosaurini* Sullivan, 2003 (converted clade name)

**Registration number:** 749

**Definition.** The largest clade containing *Pachycephalosaurus wyomingensis* (Gilmore, 1931) but not *Prenocephale prenes* Maryańska & Osmólska, 1974 and *Sphaerotholus goodwini* Williamson & Carr, 2003. This is a maximum-clade definition. Abbreviated definition: max ∇ (*Pachycephalosaurus wyomingensis* (Gilmore, 1931) ~ *Prenocephale prenes* Maryańska & Osmólska, 1974 & *Sphaerotholus goodwini* Williamson & Carr, 2003).

**Reference phylogeny.** Figure 27 of Schott & Evans (2017) is treated here as the primary reference phylogeny. Additional reference phylogenies include Figure 7 of Longrich, Sankey & Tanke (2010), Figure 5 of Evans et al. (2013), Figure 3 of Williamson & Brusatte (2016), and Figure 14 of Woodruff et al. (2021).

**Composition.** Under the primary reference phylogeny, *Pachycephalosaurini* comprises *Alaskacephale gongloffi*, *Dracorex hogwartsia*, *Pachycephalosaurus wyomingensis*, and *Stygimoloch spinifer*.

**Synonyms.** No other taxon names are currently in use for the same or approximate clade.

**Comments.** The name *Pachycephalosaurini* has been (informally) defined before by Sereno (2005) who applied a minimum-clade definition and used *Pachycephalosaurus wyomingensis* and *Stygimoloch spinifer* as the internal specifiers. Even though such definition is congruent with the original intent of Sullivan (2003) who established the taxon name to unite *S. spinifer* and *P. wyomingensis*, it has since been hypothesized that *S. spinifer* and *P. wyomingensis* may represent different growth stages of a single taxon (*P. wyomingensis*) rather than two distinct taxa (Horner & Goodwin, 2009). Nevertheless, the name *Pachycephalosaurini* may still be considered useful as recent studies indicate close relationships between *P. wyomingensis* and *Alaskacephale gangloffi* (Longrich, Sankey & Tanke, 2010; Evans et al., 2013; Williamson & Brusatte, 2016; Schott & Evans, 2017; Woodruff et al., 2021). Owing to the unsettled phylogenetic ties between the latest Cretaceous pachycephalosaurs, we prefer to establish a maximum-clade definition for *Pachycephalosaurini* to enable the name to be used for a wider range of late-diverging members of *Pachycephalosauridae*.

### *Pachyrhinosaurini* Fiorillo & Tykoski, 2012 (converted clade name)

**Registration number:** 690

**Definition.** The largest clade containing *Pachyrhinosaurus canadensis* Sternberg, 1950 but not *Centrosaurus apertus* Lambe, 1905. This is a maximum-clade definition. Abbreviated definition: max ∇ (*Pachyrhinosaurus canadensis* Sternberg, 1950 ~ *Centrosaurus apertus* Lambe, 1905).

**Reference phylogeny.** Figure 9 of Chiba et al. (2018) is treated here as the primary reference phylogeny. Additional reference phylogenies include Figure 7 of Fiorillo & Tykoski (2012), Figure 10 of Ryan et al. (2017), Figure 13 of Dalman et al. (2018), Figure 10 of Wilson, Ryan & Evans (2020), and Figure 23 of Dalman et al. (2021).

**Composition.** Under the primary reference phylogeny, *Pachyrhinosaurini* comprises *Einiosaurus procurvicornis* and members of the clade *Pachyrostra*.

**Synonyms.** No other taxon names are currently in use for the same or approximate clade.

**Comments.** The name was first (informally) defined by Fiorillo & Tykoski (2012) who applied the maximum-clade definition and used *Pachyrhinosaurus canadensis* as the internal specifier and *Centrosaurus apertus* as the external specifier. We formalize this definition.

### *Pachyrostra* Fiorillo & Tykoski, 2012 (converted clade name)

**Registration number:** 691

**Definition.** The smallest clade containing *Achelousaurus horneri* Sampson, 1995 and *Pachyrhinosaurus canadensis* Sternberg, 1950. This is a minimum-clade definition. Abbreviated definition: min ∇ (*Achelousaurus horneri* Sampson, 1995 & *Pachyrhinosaurus canadensis* Sternberg, 1950).

**Reference phylogeny.** Figure 9 of Chiba et al. (2018) is treated here as the primary reference phylogeny. Additional reference phylogenies include Figure 7 of Fiorillo & Tykoski (2012), Figure 10 of Ryan et al. (2017), Figure 13 of Dalman et al. (2018), Figure 10 of Wilson, Ryan & Evans (2020), and Figure 23 of Dalman et al. (2021).

**Composition.** Under the primary reference phylogeny, *Pachyrostra* comprises *Achelousaurus horneri* and *Pachyrhinosaurus* spp.

**Synonyms.** No other taxon names are currently in use for the same or approximate clade.

**Comments.** The name was first (informally) defined by Fiorillo & Tykoski (2012) who applied the minimum-clade definition and used *Achelousaurus horneri* and *Pachyrhinosaurus canadensis* as the internal specifiers. We formalize this definition.

### *Panoplosaurini* (new clade name)

**Registration number:** 644

**Definition.** The largest clade containing *Panoplosaurus mirus* Lambe, 1919 but not *Nodosaurus textilis* Marsh, 1889 and *Struthiosaurus austriacus* Bunzel, 1871. This is a maximum-clade definition. Abbreviated definition: max ∇ (*Panoplosaurus mirus* Lambe, 1919 ~ *Nodosaurus textilis* Marsh, 1889 & *Struthiosaurus austriacus* Bunzel, 1871).

**Etymology.** Derived from the stem of *Panoplosaurus* Lambe, 1919, the name of an included taxon, which combines the Greek words *pan* (all), *hoplon* (type of shield), and *sauros* (lizard, reptile).

**Reference phylogeny.** Figure 5 of Rivera-Sylva et al. (2018a) is treated here as the primary reference phylogeny. Additional reference phylogenies include Figure 1 of Arbour, Zanno & Gates (2016), Figure 3 of Brown et al. (2017), and Figure 9 of Zheng et al. (2018).

**Composition.** Under the primary reference phylogeny, *Panoplosaurini* comprises *Animantarx ramaljonesi*, ‘*Denversaurus*’ *schlessmani*, *Edmontonia longiceps*, *Edmontonia rugosidens*, *Panoplosaurus mirus*, *Texasetes pleurohalio*, and the ‘Argentinian ankylosaur’.

**Synonyms.** The name *Panoplosaurinae* Nopcsa, 1929 has been recently suggested for the same clade (*e.g*., Rivera-Sylva et al., 2018a; see also ‘Comments’ below). Additionally, Bakker (1988) coined the name *Edmontoniinae* for *Edmontonia rugosidens*, *Edmontonia longiceps*, and *Denversaurus schlessmani* and *Edmontoniidae* to include *Edmontoniinae* and *Panoplosaurinae*; no phylogenetic definition was proposed for either and neither clade name has been widely used since.

**Comments.** The grouping, here covered under the name *Panoplosaurini*, has previously been suggested to be named *Panoplosaurinae* (Rivera-Sylva et al., 2018a). No (informal) phylogenetic definition for *Panoplosaurinae* has ever been published in the peer-reviewed literature, though Burns (2015) proposed “all Late Cretaceous nodosaurids more closely related to *Panoplosaurus* than to *Pawpawsaurus*” in his dissertation, and the name itself has not been widely used. Bakker (1988) provided a diagnosis of *Panoplosaurinae*, as nodosaurids with lumpy armor and expanded internarial bridges, which contained the two species of *Panoplosaurus* he recognized (*Panoplosaurus mirus* and *Panoplosaurus* sp. 1, represented by ROM 1215). Alpha taxonomic reviews of the Campanian-Maastrichtian North American nodosaurids generally recognize *Panoplosaurus mirus*, *Edmontonia rugosidens*, and *Edmontonia longiceps* as valid taxa (*e.g*., Carpenter, 2001) and these typically form a clade (*e.g*. Kirkland, 1998, Vickaryous, Maryanska & Weishampel, 2004; Thompson et al., 2012; Yang et al., 2013), sometimes with additional taxa such as *Texasetes* (Arbour, Zanno & Gates, 2016; Rivera-Sylva et al., 2018a) or *Animantarx* (Hill, Witmer & Norell, 2003). Rivera-Sylva et al. (2018a) noted that the grouping *Animantarx*, ‘*Denversaurus*’, *Edmontonia*, *Panoplosaurus*, *Texasetes*, and an unnamed Argentinian ankylosaur could bear the name *Panoplosaurinae*. In several recent analyses *Edmontonia* and *Panoplosaurus* are found as the sister clade to a clade containing *Struthiosaurus* (Arbour, Zanno & Gates, 2016; Brown et al., 2017; Rivera-Sylva et al., 2018a), here named *Struthiosaurini* (see the name entry). Owing to the fact that the ‘*Panoplosaurus* clade’ is nested within *Nodosaurinae*, we prefer to use a name that implies a lesser inclusiveness. The suffix -*inae* (as in *Panoplosaurinae*) is typically associated with the rank of ‘subfamily’ under the *ICZN*. Therefore, the use of the suffix ‘-inae’ for the ‘*Panoplosaurus* clade’, without discussing the phylogenetic context, may suggest that *Panoplosaurinae* represents a clade outside *Nodosaurinae*. When the widely used suffix -*ini* (typically associated with the rank of ‘tribe’) is applied, such confusion is eliminated.

### *Parasaurolophini* Glut, 1997 (converted clade name)

**Registration number:** 645

**Definition.** The largest clade containing *Parasaurolophus walkeri* Parks, 1922 but not *Aralosaurus tuberiferus* Rozhdestvensky, 1968, *Lambeosaurus lambei* Parks, 1923 and *Tsintaosaurus spinorhinus* Young, 1958. This is a maximum-clade definition. Abbreviated definition: max ∇ (*Parasaurolophus walkeri* Parks, 1922 ~ *Aralosaurus tuberiferus* Rozhdestvensky, 1968 & *Lambeosaurus lambei* Parks, 1923 & *Tsintaosaurus spinorhinus* Young, 1958).

**Reference phylogeny.** Figure 18 of Prieto-Márquez, Wagner & Lehman (2020) is treated here as the primary reference phylogeny. Additional reference phylogenies include Figure 5 of Kobayashi et al. (2019), Figure 11 of Prieto-Márquez et al. (2019), Figure 9 of Zhang et al. (2019), Figure 5 of Zhang et al. (2020), Figure 7 of Kobayashi et al. (2021), and Figure 10 of Longrich et al. (2021).

**Composition.** Under the primary reference phylogeny, *Parasaurolophini* comprises *Charonosaurus jiayinensis* and *Parasaurolophus* spp.

**Synonyms.** No other taxon names are currently in use for the same or approximate clade.

**Comments.** The name was first (informally) defined by Prieto-Márquez et al. (2013) who applied the maximum-clade definition and used *Parasaurolophus walkeri* as the internal specifier and *Lambeosaurus lambei*, *Tsintaosaurus spinorhinus*, and *Aralosaurus tuberiferus* as the external specifiers. We formalize this definition.

### *Polacanthinae* Lapparent & Lavocat, 1955 (converted clade name)

**Registration number:** 646

**Definition.** The largest clade within *Ankylosauridae* or *Nodosauridae* containing *Polacanthus foxii* Owen in Anonymous, 1865 but not *Ankylosaurus magniventris* Brown, 1908 and *Nodosaurus textilis* Marsh, 1889. This is a maximum-clade definition. Abbreviated definition: max ∇ ∈ *Ankylosauridae* ∨ *Nodosauridae* (*Polacanthus foxii* Owen in Anonymous, 1865 ~ *Ankylosaurus magniventris* Brown, 1908 & *Nodosaurus textilis* Marsh, 1889).

**Reference phylogeny.** Figure 9 of Yang et al. (2013) is treated here as the primary reference phylogeny. Additional reference phylogenies include Figure 3 of Kirkland (1998), Figure 2 of Thompson et al. (2012), Figure 1 of Arbour, Zanno & Gates (2016), Figure 5 of Rivera-Sylva et al. (2018a), and Figure 9 of Zheng et al. (2018).

**Composition.** Under the primary reference phylogeny, *Polacanthinae* comprises *Polacanthus foxii* and *Taohelong jinchengensis*.

**Synonyms.** Jaekel (1910) introduced the name *Polacanthidae* to include ankylosaurs that appeared intermediate between *Ankylosauridae* and *Nodosauridae*. Kirkland (1998) was the first to assess ‘polacanthids’ using cladistic methods and found them to be a clade of early-diverging ankylosaurids, and as such should preferably be called *Polacanthinae* rather than *Polacanthidae*, to eliminate the possible confusion that *Ankylosauridae* and *Polacanthidae* refer to mutually exclusive clades. Carpenter (2001) argued that *Polacanthidae* was instead valid and defined the name to cover all ankylosaurs closer to *Gastonia* than to *Edmontonia* or *Euoplocephalus*.

**Comments.** The name *Polacanthinae* was (informally) defined before by Yang et al. (2013), who used *Polacanthus foxii* as the internal specifier and *Ankylosaurus magniventris* and *Panoplosaurus mirus* as the external specifiers. Kirkland (1998) diagnosed *Polacanthinae* as comprising ankylosaurs with an ankylosaurid-like skulls, nearly straight and parallel tooth rows, long basipterygoid processes, well-developed acromion arising from dorsal margin of scapula, ventrally flexed ischia, coossified pelvic osteoderms forming pelvic shield, posteriorly grooved and elongate pectoral osteoderms, and caudal osteoderms large, elongate laterally directed, and with hollow bases. Kirkland (1998) initially found *Polacanthinae* at the base of *Ankylosauridae* including *Gastonia*, *Polacanthus*, and *Mymoorapelta* and also referred *Hoplitosaurus* and *Hylaeosaurus* to the clade. Arbour, Zanno & Gates (2016), Rivera-Sylva et al. (2018a), and Zheng et al. (2018) inferred what could be called *Polacanthinae* at the base of *Nodosauridae*, including *Polacanthus foxii* and *Hoplitosaurus marshi*. *Polacanthinae* is poorly supported in most phylogenetic analyses yet frequently referenced in the literature. Taxa typically referred to as ‘polacanthines’ most often form a grade of early-diverging nodosaurids (*e.g*., Thompson et al., 2012; Brown et al., 2017). Additional taxonomic and phylogenetic revisions are needed to provide an assessment of *Polacanthinae*. We define the name here to ensure that it is applicable either within *Ankylosauridae* or *Nodosauridae*. If the ‘*Polacanthus* clade’ is reconstructed outside the *Ankylosauridae* + *Nodosauridae* node, the name *Polacanthinae* becomes inapplicable and the preferred name for the grouping should probably be *Polacanthidae* (not defined here).

### *Protoceratopsidae* Granger & Gregory, 1923 (converted clade name)

**Registration number:** 647

**Definition.** The largest clade containing *Protoceratops andrewsi* Granger & Gregory, 1923 but not *Ceratops montanus* Marsh, 1888, *Leptoceratops gracilis* Brown, 1914b, and *Triceratops horridus* Marsh, 1889. This is a maximum-clade definition. Abbreviated definition: max ∇ (*Protoceratops andrewsi* Granger & Gregory, 1923 ~ *Ceratops montanus* Marsh, 1888 & *Leptoceratops gracilis* Brown, 1914b & *Triceratops horridus* Marsh, 1889).

**Reference phylogeny.** Figure 10 of Morschhauser et al. (2019) is treated here as the primary reference phylogeny. Additional reference phylogenies include Figure S1 of Knapp et al. (2018), Figure 8A of Arbour & Evans (2019), Figure 3 of Yu et al. (2020), and Figure 4 of Yu et al. (2020).

**Composition.** Under the primary reference phylogeny, *Protoceratopsidae* comprises *Bagaceratops rozhdestvenskyi*, *Magnirostris dodsoni* (?= *Bagaceratops rozhdestvenskyi*; see Czepiński, 2020), and *Protoceratops* spp.

**Synonyms.** No other taxon names are currently in use for the same or approximate clade.

**Comments.** The name *Protoceratopsidae* has been (informally) defined before by Sereno (1998, 2005) who applied a maximum-clade definition and used *Protoceratops andrewsi* as the internal specifier and *Triceratops horridus* as the external specifier. We include two additional external specifiers *Ceratops montanus* and *Leptoceratops gracilis. C. montanus* was added because the name *Protoceratopsidae* has been traditionally applied to the sister taxon of *Ceratopsoidea*, and *L. gracilis* was included to ensure that *Protoceratopsidae* and *Leptoceratopsidae* remain mutually exclusive clades.

### *Rhabdodontidae* Weishampel et al., 2003 (converted clade name)

**Registration number:** 648

**Definition.** The smallest clade containing *Rhabdodon priscus* Matheron, 1869 and *Zalmoxes robustus* (Nopcsa, 1900). This is a minimum-clade definition. Abbreviated definition: min ∇ (*Rhabdodon priscus* Matheron, 1869 & *Zalmoxes robustus* (Nopcsa, 1900)).

**Reference phylogeny.** Figure 4 of Madzia, Boyd & Mazuch (2018) is treated here as the primary reference phylogeny. Additional reference phylogenies include Figure 3 of Madzia, Boyd & Mazuch (2018), Figure 20 of Verdú et al. (2018), Figure 25 of Herne et al. (2019), Figure 3 of Párraga & Prieto-Márquez (2019), Figure 2 of Dieudonné et al. (2020), and Figure 9 of Verdú et al. (2020).

**Composition.** Under the primary reference phylogeny, *Rhabdodontidae* comprises *Rhabdodon priscus*, *Zalmoxes robustus*, *Zalmoxes shqiperorum*, *Mochlodon suessi*, and *Mochlodon vorosi*.

**Synonyms.** No other taxon names are currently in use for the same or approximate clade.

**Comments.** The name *Rhabdodontidae* was first (informally) defined by Weishampel et al. (2003: 69) who used the minimum-clade definition and selected *Rhabdodon priscus* and *Zalmoxes robustus* as the internal specifiers. Sereno (2005) later used a maximum-clade definition, using *Rhabdodon priscus* as the internal specifier and *Parasaurolophus walkeri* as the external specifier. We formalize the former, minimum-clade, definition. A definition similar in effect to that of Sereno (2005) is applied to *Rhabdodontomorpha*.

### *Rhabdodontomorpha* Dieudonné et al., 2016 (converted clade name)

**Registration number:** 649

**Definition.** The largest clade containing *Rhabdodon priscus* Matheron, 1869 but not *Hypsilophodon foxii* Huxley, 1869 and *Iguanodon bernissartensis* Boulenger in Beneden, 1881. This is a maximum-clade definition. Abbreviated definition: max ∇ (*Rhabdodon priscus* Matheron, 1869 ~ *Hypsilophodon foxii* Huxley, 1869 & *Iguanodon bernissartensis* Boulenger in Beneden, 1881).

**Reference phylogeny.** Figure 2 of Dieudonné et al. (2020) is treated here as the primary reference phylogeny. Additional reference phylogenies include Figure 4 of Madzia, Boyd & Mazuch (2018), Figure 25 of Herne et al. (2019), and Figure 57 of Barta & Norell (2021).

**Composition.** Under the primary reference phylogeny, *Rhabdodontomorpha* comprises *Muttaburrasaurus langdoni*, *Fostoria dhimbangunmal*, the ‘Vegagete ornithopod’, and members of the clade *Rhabdodontidae*.

**Synonyms.** No other taxon names are currently in use for the same or approximate clade.

**Comments.** The application of *Rhabdodontomorpha* has been described, and (informally) proposed definitions have been discussed, by Madzia, Boyd & Mazuch (2018: Appendix 1) and Madzia, Jagt & Mulder (2020: Table 1). We therefore refer to these studies for details. Our formalized maximum-clade definition is similar to that of Madzia, Jagt & Mulder (2020) in that it uses *Rhabdodon priscus* as the internal specifier and *Iguanodon bernissartensis* as the external specifier. We have further added a second external specifier, *Hypsilophodon foxii*, to prevent its inclusion to *Rhabdodontomorpha* under phylogenies similar to that of Norman (2015: Fig. 50).

### *Saphornithischia* (new clade name)

**Registration number:** 747

**Definition.** The smallest clade containing *Heterodontosaurus tucki* Crompton & Charig, 1962, *Iguanodon bernissartensis* Boulenger in Beneden, 1881, *Stegosaurus stenops* Marsh, 1887, and *Triceratops horridus* Marsh, 1889. This is a minimum-clade definition. Abbreviated definition: min ∇ (*Heterodontosaurus tucki* Crompton & Charig, 1962 & *Iguanodon bernissartensis* Boulenger in Beneden, 1881 & *Stegosaurus stenops* Marsh, 1887 & *Triceratops horridus* Marsh, 1889).

**Etymology.** Derived from the Greek *safis* (clear, definite) and formed to include all members of *Ornithischia* whose placement within the clade is well established.

**Reference Phylogeny.** Figure 4 of Madzia, Boyd & Mazuch (2018) is treated here as the primary reference phylogeny. Additional reference phylogenies include Figure 25 of Herne et al. (2019), Yang et al. (2020), and Figure 57 of Barta & Norell (2021).

**Composition.** Under the primary reference phylogeny, *Saphornithischia* comprises *Pisanosaurus mertii* and members of the clades *Heterodontosauridae* and *Genasauria*.

**Synonyms.** Under alternative topologies, where *Heterodontosauridae* is reconstructed within *Neornithischia* (*e.g*., Butler, 2005; Xu et al., 2006; Dieudonné et al., 2020), *Saphornithischia* would be a heterodefinitional synonym of *Genasauria*.

**Comments.** Given the repeated inference of heterodontosaurids outside *Genasauria* in multiple studies (*e.g*., Butler, Upchurch & Norman, 2008; Boyd, 2015; Han et al., 2018; Madzia, Boyd & Mazuch, 2018; Andrzejewski, Winkler & Jacobs, 2019; Herne et al., 2019; Yang et al., 2020) and the uncertainty surrounding the potential ornithischian affinities of *Pisanosaurus mertii* (Agnolín & Rozadilla, 2018, Baron, 2019; Desojo et al., 2020) and members of the *Silesauridae* (Langer & Ferigolo, 2013; Cabreira et al., 2016; Pacheco et al., 2019; Müller & Garcia, 2020), we provide a new clade name to cover taxa whose placement within *Ornithischia* is well-supported. Note that the internal specifiers *Stegosaurus stenops* and *Triceratops horridus* are not included in the primary reference phylogeny. The former belongs to *Thyreophora* (*e.g*., Maidment et al., 2020), while the latter is part of *Marginocephalia* (see, *e.g*., Morschhauser et al., 2019; Fowler & Freedman Fowler, 2020). Both these clades are indicated on Figure 4 of Madzia, Boyd & Mazuch (2018).

### *Saurolophinae* Brown, 1914a (converted clade name)

**Registration number:** 650

**Definition.** The largest clade containing *Saurolophus osborni* Brown, 1912 but not *Lambeosaurus lambei* Parks, 1923, provided that it does not include *Hadrosaurus foulkii* Leidy, 1858. This is a maximum-clade definition. Abbreviated definition: max ∇ (*Saurolophus osborni* Brown, 1912 ~ *Lambeosaurus lambei* Parks, 1923 | ~ *Hadrosaurus foulkii* Leidy, 1858).

**Reference phylogeny.** Figure 18 of Prieto-Márquez, Wagner & Lehman (2020) is treated here as the primary reference phylogeny. Additional reference phylogenies include Figure 5 of Kobayashi et al. (2019), Figure 11 of Prieto-Márquez et al. (2019), Figure 9 of Zhang et al. (2019), Figure 5 of Zhang et al. (2020), Figure 7 of Kobayashi et al. (2021), and Figure 10 of Longrich et al. (2021).

**Composition.** Under the primary reference phylogeny, *Saurolophinae* comprises ?*Gryposaurus alsatei*, *Naashoibitosaurus ostromi*, members of the clades *Brachylophosaurini*, *Edmontosaurini*, *Kritosaurini*, and *Saurolophini*, and the specimen ‘PASAC 1 (‘Sabinosaur’)’.

**Figure 5:**
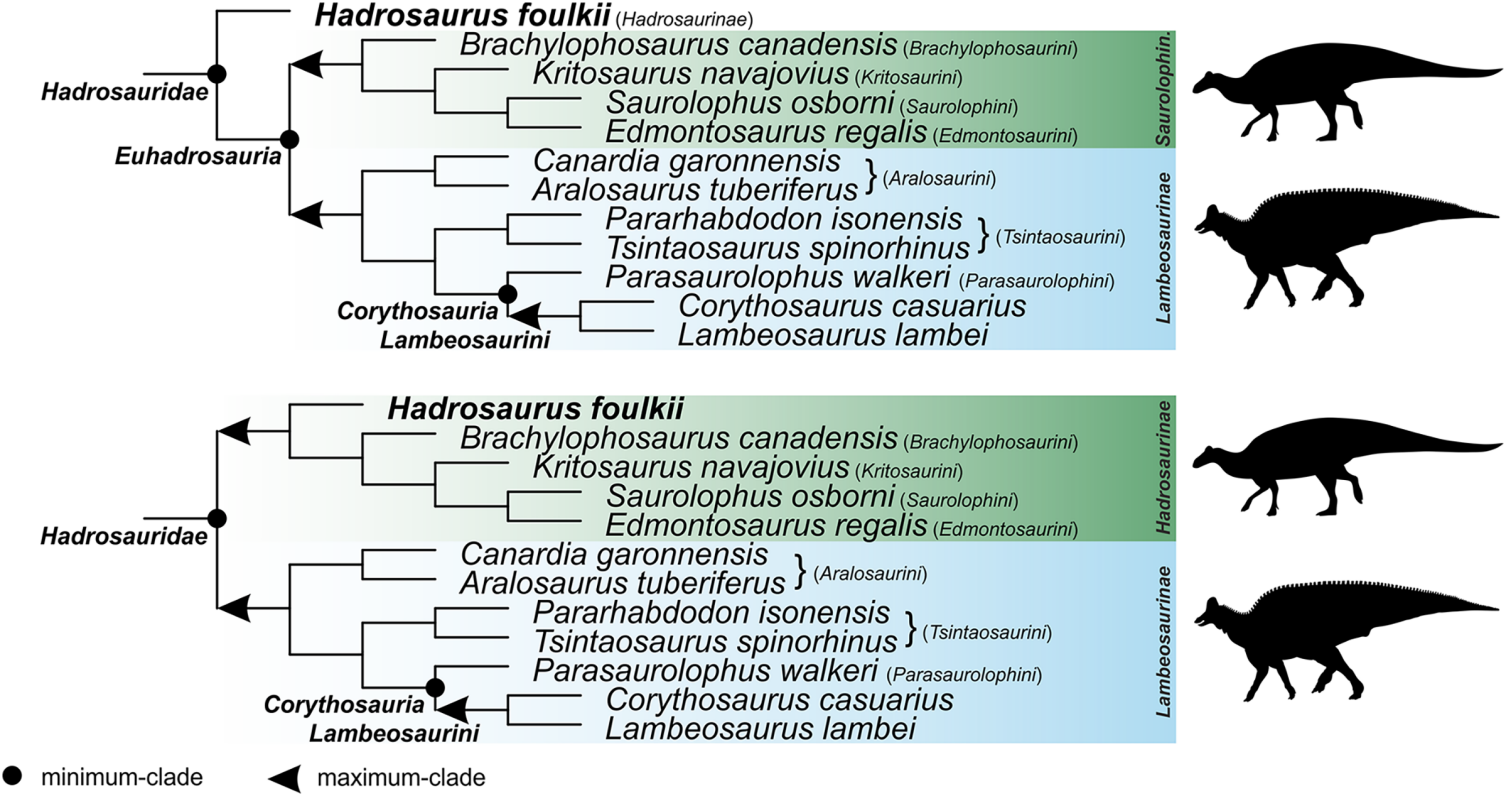
Specifier-based phylogeny of *Hadrosauridae* showing alternative placements of *Hadrosaurus foulkii*. The silhouette of *Lambeosaurinae* was obtained from phylopic.org (Dmitry Bogdanov, CC BY 3.0). The silhouette of *Hadrosaurinae*/*Saurolophinae* was prepared by Victoria M. Arbour (CC BY 4.0).

**Synonyms.** Following the widespread application of the Principle of Coordination, under which *Hadrosaurinae* has to be attributed to Cope (1869), the name *Hadrosaurinae* is generally considered to have priority over *Saurolophinae*, even though the latter was coined 4 years earlier (*Saurolophinae* Brown, 1914a; *Hadrosaurinae* Lambe, 1918). In recent years, both *Hadrosaurinae* and *Saurolophinae*, have been used for the sister taxon of *Lambeosaurinae*. The selection of the proper name has traditionally depended on whether the clade includes *Hadrosaurus foulkii* or not (Fig. 5). In the cases in which *H. foulkii* falls within the smallest clade containing *Saurolophus osborni* and *Lambeosaurus lambei*, and within the ‘*Saurolophus* branch’, the name *Hadrosaurinae* is preferred (*e.g*., Cruzado-Caballero & Powell, 2017; Xing, Mallon & Currie, 2017; Kobayashi et al., 2019; Zhang et al., 2020). However, when *H. foulkii* falls outside the clade, the name *Saurolophinae* is used (*e.g*., Prieto-Márquez et al., 2019; Prieto-Márquez, Wagner & Lehman, 2020; Gates, Evans & Sertich, 2021; Kobayashi et al., 2021; McDonald et al., 2021; Ramírez-Velasco et al., 2021).

**Comments.** The name *Saurolophinae* has been (informally) defined before by Prieto-Márquez (2010) who applied a maximum-clade definition and used *Saurolophus osborni* as the internal specifier and *Lambeosaurus lambei* and *Hadrosaurus foulkii* as the external specifiers. Here, we formalize a maximum-clade definition of *Saurolophinae* that applies the name to the sister clade of *Lambeosaurinae* only on the condition that it does not contain *H. foulkii*. In turn, the name *Hadrosaurinae* is defined to be used for the ‘*Saurolophus* branch’ when *H. foulkii* falls within the clade. Although our definition may be considered similar to that of Prieto-Márquez (2010) it differs substantially because under our definition, the name *Saurolophinae* may become inapplicable.

### *Saurolophini* Glut, 1997 (converted clade name)

**Registration number:** 651

**Definition.** The largest clade containing *Saurolophus osborni* Brown, 1912 but not *Brachylophosaurus canadensis* Sternberg, 1953, *Edmontosaurus regalis* Lambe, 1917, *Hadrosaurus foulkii* Leidy, 1858, and *Kritosaurus navajovius* Brown, 1910. This is a maximum-clade definition. Abbreviated definition: max ∇ (*Saurolophus osborni* Brown, 1912 ~ *Brachylophosaurus canadensis* Sternberg, 1953 & *Edmontosaurus regalis* Lambe, 1917 & *Hadrosaurus foulkii* Leidy, 1858 & *Kritosaurus navajovius* Brown, 1910).

**Reference phylogeny.** Figure 18 of Prieto-Márquez, Wagner & Lehman (2020) is treated here as the primary reference phylogeny. Additional reference phylogenies include Figure 5 of Kobayashi et al. (2019), Figure 11 of Prieto-Márquez et al. (2019), Figure 9 of Zhang et al. (2019), Figure 5 of Zhang et al. (2020), Figure 7 of Kobayashi et al. (2021), and Figure 10 of Longrich et al. (2021).

**Composition.** Under the primary reference phylogeny, *Saurolophini* comprises *Augustynolophus morrisi*, *Prosaurolophus maximus*, and *Saurolophus* spp.

**Synonyms.** No other taxon names are currently in use for the same or approximate clade.

**Comments.** The name *Saurolophini* has been (informally) defined before (Sereno, 2005; Prieto-Márquez et al., 2014). Both these definitions were maximum-clade and used *Saurolophus osborni* as the internal specifier and *Edmontosaurus regalis* and *Maiasaura peeblesorum* (Sereno, 2005) or *Brachylophosaurus canadensis*, *Edmontosaurus regalis*, *Kritosaurus navajovius*, and *Lambeosaurus lambei* (Prieto-Márquez et al., 2014) as the external specifiers. Here we apply a definition similar to that of Prieto-Márquez et al. (2014) but remove *L. lambei* and instead add *Hadrosaurus foulkii*.

### *Shamosaurinae* Tumanova, 1983 (converted clade name)

**Registration number:** 652

**Definition.** The largest clade containing *Gobisaurus domoculus* Vickaryous et al., 2001 and *Shamosaurus scutatus* Tumanova, 1983 but not *Ankylosaurus magniventris* Brown, 1908. This is a maximum-clade definition. Abbreviated definition: max ∇ (*Gobisaurus domoculus* Vickaryous et al., 2001 & *Shamosaurus scutatus* Tumanova, 1983 ~ *Ankylosaurus magniventris* Brown, 1908).

**Reference phylogeny.** Figure 11 of Arbour & Currie (2016) is treated here as the primary reference phylogeny. Additional reference phylogenies include Figure 1 of Arbour, Zanno & Gates (2016), Figure 8 of Arbour & Evans (2017), and Figure 5 of Rivera-Sylva et al. (2018a).

**Composition.** Under the primary reference phylogeny, *Shamosaurinae* comprises *Gobisaurus domoculus* and *Shamosaurus scutatus*.

**Synonyms.** No other taxon names are currently in use for the same or approximate clade.

**Comments.** Tumanova (1987) described *Shamosaurinae* based on a list of diagnostic features: shamosaurines were ankylosaurids with narrow anterior snouts, angle of the orbital plane with the skull axis less than 25°, anterior wall of the pterygoid inclined posteriorly, occipital condyle a wide oval, pterygoids fused with the basisphenoid, small interpterygoid fenestra, and orbits at the midlength of the skull. *Shamosaurinae* is not reconstructed in all recent phylogenetic analyses, as *Shamosaurus* and *Gobisaurus* are sometimes inferred as successive outgroups to *Ankylosaurinae* rather than as a clade (*e.g*., Thompson et al., 2012; Wiersma & Irmis, 2018). We provide a maximum-clade definition that makes *Shamosaurinae* applicable only under the topologies in which *Shamosaurus* and *Gobisaurus* are more closely related to each other than either is to *Ankylosaurus*.

### *Stegosauria* Marsh, 1877b (converted clade name)

**Registration number:** 653

**Definition.** The largest clade containing *Stegosaurus stenops* Marsh, 1887 but not *Ankylosaurus magniventris* Brown, 1908. This is a maximum-clade definition. Abbreviated definition: max ∇ (*Stegosaurus stenops* Marsh, 1887 ~ *Ankylosaurus magniventris* Brown, 1908).

**Reference phylogeny.** Figure 12 of Maidment et al. (2020) is treated here as the primary reference phylogeny. Additional reference phylogenies include Figure 11 of Maidment et al. (2008), Figure 1 of Raven & Maidment (2017), and Figure 1 of Dieudonné et al. (2020).

**Composition.** Under the primary reference phylogeny, *Stegosauria* comprises *Isaberrysaura mollensis*, *Gigantspinosaurus sichuanensis*, and members of the clades *Stegosauridae* and *Huayangosauridae*.

**Synonyms.** No other taxon names are currently in use for the same or approximate clade.

**Comments.** The name *Stegosauria* has been (informally) defined before (Galton, 1997; Sereno, 1998; Galton & Upchurch, 2004; Sereno, 2005) using *Stegosaurus* (Galton, 1997; Sereno, 1998; Galton & Upchurch, 2004) or *Stegosaurus stenops* (Sereno, 2005) as the internal specifier and *Ankylosaurus* (Galton, 1997; Sereno, 1998), *Ankylosauria* (Galton & Upchurch, 2004), or *Ankylosaurus magniventris* (Sereno, 2005) as the external specifiers. Since *Stegosauria* has never been proposed an alternative use, we apply *S. stenops* as the internal specifier and *A. magniventris* as the external specifier. Note that *A. magniventris* is not included in the primary reference phylogeny. See Figure 3 of Thompson et al. (2012) for its placement with respect to *Stegosauria*.

### *Stegosauridae* Marsh, 1880 (converted clade name)

**Registration number:** 654

**Definition.** The largest clade containing *Stegosaurus stenops* Marsh, 1887 but not *Huayangosaurus taibaii* Dong, Tang & Zhou, 1982. This is a maximum-clade definition. Abbreviated definition: max ∇ (*Stegosaurus stenops* Marsh, 1887 ~ *Huayangosaurus taibaii* Dong, Tang & Zhou, 1982).

**Reference phylogeny.** Figure 12 of Maidment et al. (2020) is treated here as the primary reference phylogeny. Additional reference phylogenies include Figure 11 of Maidment et al. (2008) and Figure 1 of Raven & Maidment (2017).

**Composition.** Under the primary reference phylogeny, *Stegosauridae* comprises *Adratiklit boulahfa*, *Alcovasaurus longispinus*, *Dacentrurus armatus*, *Hesperosaurus mjosi*, *Jiangjunosaurus junggarensis*, *Kentrosaurus aethiopicus*, *Loricatosaurus priscus*, *Miragaia longicollum*, *Paranthodon africanus*, *Stegosaurus homheni*, *Stegosaurus stenops*, and *Tuojiangosaurus multispinus*.

**Synonyms.** No other taxon names are currently in use for the same or approximate clade.

**Comments.** The name *Stegosauridae* was first (informally) defined by Sereno (1998, 2005) who used the maximum-clade definition and selected *Stegosaurus stenops* as the internal specifier and *Huayangosaurus taibaii* as the external specifier. We formalize this definition.

### *Struthiosaurini* (new clade name)

**Registration number:** 655

**Definition.** The largest clade containing *Struthiosaurus austriacus* Bunzel, 1871 but not *Nodosaurus textilis* Marsh, 1889 and *Panoplosaurus mirus* Lambe, 1919. This is a maximum-clade definition. Abbreviated definition: max ∇ (*Struthiosaurus austriacus* Bunzel, 1871 ~ *Nodosaurus textilis* Marsh, 1889 & *Panoplosaurus mirus* Lambe, 1919).

**Etymology.** Derived from the stem of *Struthiosaurus* Bunzel, 1871, the name of an included taxon, which combines the Latin word *struthio* (ostrich) and Greek *sauros* (lizard, reptile).

**Reference phylogeny.** Figure 5 of Rivera-Sylva et al. (2018a) is treated here as the primary reference phylogeny. Additional reference phylogenies include Figure 1 of Arbour, Zanno & Gates (2016), Figure 3 of Brown et al. (2017), and Figure 9 of Zheng et al. (2018).

**Composition.** Under the primary reference phylogeny, *Struthiosaurini* comprises *Europelta carbonensis*, *Hungarosaurus tormai*, *Pawpawsaurus campbelli*, *Stegopelta landerensis*, and *Struthiosaurus* spp.

**Synonyms.** The name *Struthiosaurinae* Nopcsa, 1923 has been recently used for an approximate clade (Kirkland et al., 2013; Blows & Honeysett, 2014; Villanueva-Amadoz et al., 2015). No other taxon names are currently in use for the same or approximate clade.

**Comments.** A grouping similar to that covered here under the name *Struthiosaurini* has previously been named *Struthiosaurinae* (Kirkland et al., 2013). The name *Struthiosaurinae* was (informally) defined by Kirkland et al. (2013) who applied the maximum-clade definition and used *Europelta* as the internal specifier and *Cedarpelta*, *Peloroplites*, *Sauropelta*, and *Edmontonia* as the external specifiers. *Struthiosaurinae* was considered to represent the clade of Late Cretaceous European nodosaurids. However, Kirkland et al. (2013) did not include a character matrix or phylogenetic analysis in their study and have not yet published a follow-up paper with results indicating the extent of their *Struthiosaurinae*. They provided, however, a list of diagnostic characters. According to Kirkland et al. (2013), *Struthiosaurinae* includes nodosaurid ankylosaurs with narrow predentaries, nearly horizontal and unfused quadrates, quadrate condyles that are 3 times transversely wider than long, premaxillary teeth and dentary teeth that are near the predentary symphysis, dorsally arched sacra, an acromion process dorsal to the midpoint of the scapulocoracoid suture, straight ischia with a straight dorsal margin, long slender limbs, a sacral shield, and erect sacral osteoderms with flat bases. This suite of characters was considered to unite *Anoplosaurus*, *Europelta*, *Hungarosaurus*, and *Struthiosaurus*, but many of these characters have a broad distribution in *Ankylosauria* and *Nodosauridae* (Ősi, 2015). Arbour, Zanno & Gates (2016) reconstructed a clade containing *Ahshislepelta*, *Europelta*, *Hungarosaurus*, *Niobrarasaurus*, *Nodosaurus*, *Pawpawsaurus*, *Stegopelta*, *Struthiosaurus*, and the ‘Paw Paw juvenile’ as the sister clade to that containing *Edmontonia*, which would thus be considered *Struthiosaurinae*. Brown et al. (2017) added *Borealopelta* to the matrix of Arbour, Zanno & Gates (2016) and reconstructed a clade of *Borealopelta*, *Europelta*, *Hungarosaurus*, and *Pawpawsaurus*; *Stegopelta* and *Struthiosaurus* were outside of this clade and sister to *Edmontonia*, ‘*Denversaurus*’, and *Panoplosaurus*. As was the case with *Panoplosaurinae*, owing to the fact that the ‘*Struthiosaurus* clade’ is nested within *Nodosaurinae*, we prefer to use a name that implies a lesser inclusiveness (that is, -*ini* rather than -*inae*). The use of *Struthiosaurinae*, without discussing the phylogenetic context, may suggest that *Struthiosaurinae* and *Nodosaurinae* are mutually exclusive clades. When the suffix -*ini* is applied, such confusion is eliminated. Note that the recent use of *Struthiosaurinae* has been largely limited to mentions of Kirkland et al. (2013) application of the name (Blows & Honeysett, 2014; Villanueva-Amadoz et al., 2015).

### *Styracosterna* Sereno, 1986 (converted clade name)

**Registration number:** 656

**Definition.** The largest clade containing *Iguanodon bernissartensis* Boulenger in Beneden, 1881 but not *Camptosaurus dispar* (Marsh, 1879). This is a maximum-clade definition. Abbreviated definition: max ∇ (*Iguanodon bernissartensis* Boulenger in Beneden, 1881 ~ *Camptosaurus dispar* (Marsh, 1879)).

**Reference phylogeny.** Figure 12 of Madzia, Jagt & Mulder (2020) is treated here as the primary reference phylogeny. Additional reference phylogenies include Figure 20 of Verdú et al. (2018), Figure 9 of Verdú et al. (2020), Figure 11 of McDonald et al. (2021), and Figure 11 of Santos-Cubedo et al. (2021).

**Composition.** Under the primary reference phylogeny, *Styracosterna* comprises *Cedrorestes crichtoni*, *Cumnoria prestwichii*, *Dakotadon lakotaensis*, *Draconyx loureioi*, *Fukuisaurus tetoriensis*, *Hippodraco scutodens*, *Iguanacolossus fortis*, *Lanzhousaurus magnidens*, *Muttaburrasaurus langdoni*, *Osmakasaurus depressus*, *Owenodon hoggii*, *Planicoxa venenica*, *Theiophytalia kerri*, *Uteodon aphanoecetes*, *Yunganglong datongensis*, and members of the clade *Hadrosauriformes*.

**Synonyms.** No other taxon names are currently in use for the same or approximate clade.

**Comments.** The name *Styracosterna* was first (informally) defined by Sereno (1998: 62) who used the maximum-clade definition and selected *Parasaurolophus* as the internal specifier and *Camptosaurus* as the external specifier. We prefer to use *Iguanodon bernissartensis* as the external specifier to maintain the ‘node-branch triplet’ (‘node-stem triplet’ of Sereno (1998: 52–54)) comprising *Ankylopollexia*, *Camptosauridae*, and *Styracosterna* (all formally defined in the present paper). The inclusion of a different external specifier does not change the extent of *Styracosterna* under any of the published phylogeny inferences.

### *Thescelosauridae* Sternberg, 1937 (converted clade name)

**Registration number:** 657

**Definition.** The largest clade containing *Thescelosaurus neglectus* Gilmore, 1913 but not *Iguanodon bernissartensis* Boulenger in Beneden, 1881, provided that it does not include *Hypsilophodon foxii* Huxley, 1869. This is a maximum-clade definition. Abbreviated definition: max ∇ (*Thescelosaurus neglectus* Gilmore, 1913 ~ *Iguanodon bernissartensis* Boulenger in Beneden, 1881 | ~ *Hypsilophodon foxii* Huxley, 1869).

**Reference phylogeny.** Figure 4 of Madzia, Boyd & Mazuch (2018) is treated here as the primary reference phylogeny. Additional reference phylogenies include Figure 25 of Herne et al. (2019) and Figure 57 of Barta & Norell (2021).

**Composition.** Under the primary reference phylogeny, *Thescelosauridae* comprises members of the clades *Thescelosaurinae* and *Orodrominae*.

**Synonyms.** The name *Parksosauridae* Buchholz, 2002 has been used recently for the same contents (Boyd, 2015; Rivera-Sylva et al., 2018b). No other taxon names are currently in use for the same or approximate clade.

**Comments.** The name *Thescelosauridae* has been (informally) defined before (Brown et al., 2013; Madzia, Boyd & Mazuch, 2018). Both these definitions were minimum-clade and used *Thescelosaurus neglectus* and *Orodromeus makelai* as the internal specifiers. Madzia, Boyd & Mazuch (2018) further added one external specifier, *Iguanodon bernissartensis*, to ensure that the name is applicable with a similar circumscription (see Madzia, Boyd & Mazuch, 2018: Appendix 1 for details). We apply a complex maximum-clade definition to ensure that *Thescelosauridae* is not inferred within *Hypsilophodontidae*; for example under the potential topology in which *Hypsilophodon* is the sister taxon to a *Thescelosaurinae* + *Orodrominae* node. Even though no such phylogenetic hypothesis has been proposed, the placement of taxa ‘traditionally’ dubbed the ‘hypsilophodonts’ is highly pliable across studies (Han et al., 2018; Madzia, Boyd & Mazuch, 2018; Andrzejewski, Winkler & Jacobs, 2019; Herne et al., 2019; Dieudonné et al., 2020; Rotatori, Moreno-Azanza & Mateus, 2020; Yang et al., 2020) and often differs significantly even under different tree-search methods applied to a single dataset. Therefore, it can be expected that phylogeny inferences of the rootward neornithischian-ornithopod transitional segment of the ornithischian phylogenetic trees may result in such topology at some point. A maximum-clade definition with a single internal specifier (*T. neglectus*) was preferred to allow *Thescelosauridae* in use regardless of the relationship of *T. neglectus* to *O. makelai*.

### *Thescelosaurinae* Sternberg, 1940 (converted clade name)

**Registration number:** 658

**Definition.** The largest clade within *Hypsilophodontidae* or *Thescelosauridae* containing *Thescelosaurus neglectus* Gilmore, 1913 but not *Hypsilophodon foxii* Huxley, 1869 and *Orodromeus makelai* Horner & Weishampel, 1988. This is a maximum-clade definition. Abbreviated definition: max ∇ ∈ *Hypsilophodontidae* ∨ *Thescelosauridae* (*Thescelosaurus neglectus* Gilmore, 1913 ~ *Hypsilophodon foxii* Huxley, 1869 & *Orodromeus makelai* Horner & Weishampel, 1988).

**Reference phylogeny.** Figure 4 of Madzia, Boyd & Mazuch (2018) is treated here as the primary reference phylogeny. Additional reference phylogenies include Figure 25 of Herne et al. (2019) and Figure 57 of Barta & Norell (2021).

**Composition.** Under the primary reference phylogeny, *Thescelosaurinae* comprises *Notohypsilophodon comodorensis*, *Parksosaurus warreni*, and *Thescelosaurus* spp.

**Synonyms.** No other taxon names are currently in use for the same or approximate clade.

**Comments.** The name *Thescelosaurinae* has been (informally) defined before (Brown & Druckenmiller, 2011; Boyd, 2015). Both these definitions were maximum-clade and used *Thescelosaurus neglectus* as the internal specifier and *Orodromeus makelai* and *Hypsilophodon foxii* (Brown & Druckenmiller, 2011) or *Orodromeus makelai* and *Parasaurolophus walkeri* (Boyd, 2015) as the external specifiers. Considering the ‘traditional concept’ of *Thescelosaurinae*, as a subclade of *Thescelosauridae*/‘hypsilophodonts’, and keeping in mind the unstable phylogenetic position of *H. foxii* (*e.g*., Madzia, Boyd & Mazuch, 2018), we apply *Thescelosaurinae* only when it is inferred either within *Thescelosauridae* or *Hypsilophodontidae* (see Article 11.14 of the *ICPN*).

### *Thyreophora* Nopcsa, 1915 (converted clade name)

**Registration number:** 659

**Definition.** The largest clade containing *Ankylosaurus magniventris* Brown, 1908 and *Stegosaurus stenops* Marsh, 1887 but not *Iguanodon bernissartensis* Boulenger in Beneden, 1881 and *Triceratops horridus* Marsh, 1889. This is a maximum-clade definition. Abbreviated definition: max ∇ (*Ankylosaurus magniventris* Brown, 1908 & *Stegosaurus stenops* Marsh, 1887 ~ *Iguanodon bernissartensis* Boulenger in Beneden, 1881 & *Triceratops horridus* Marsh, 1889).

**Reference phylogeny.** Figure 16 of Han et al. (2018) is treated here as the primary reference phylogeny. Additional reference phylogenies include Figure 4 of Madzia, Boyd & Mazuch (2018), Figure 25 of Herne et al. (2019), Figure 1 of Dieudonné et al. (2020), Figure 12 of Yang et al. (2020), and Figure 57 of Barta & Norell (2021).

**Composition.** Under the primary reference phylogeny, *Thyreophora* comprises *Scutellosaurus lawleri*, *Emausaurus ernsti*, *Scelidosaurus harrisonii*, and members of the clade *Eurypoda*.

**Synonyms.** No other taxon names are currently in use for the same or approximate clade.

**Comments.** The name *Thyreophora* has been (informally) defined before (Sereno, 1998; Sereno, 2005; Norman, 2021). All these definitions were maximum-clade. The definitions of Sereno (1998, 2005) used *Ankylosaurus* (Sereno, 1998) or *Ankylosaurus magniventris* (Sereno, 2005) as the internal specifier, and *Triceratops* (Sereno, 1998) or *Triceratops horridus*, *Parasaurolophus walkeri*, and *Pachycephalosaurus wyomingensis* (Sereno, 2005) as the external specifiers. In turn, Norman (2021) defined *Thyreophora* using *Euoplocephalus* and *Stegosaurus* as the internal specifiers and *Hypsilophodon* as the external specifier. In order to maintain the ‘traditional’ concept of *Genasauria* as a clade comprising *Neornithischia* and *Thyreophora*, the internal specifiers in the definition of *Thyreophora* are used from among the taxa representing the two major subclades – *Ankylosauria* (*Ankylosaurus magniventris*) and *Stegosauria* (*Stegosaurus stenops*) – and the external specifiers are used from among the taxa representing the neornithischian clades *Ornithopoda* (*Iguanodon bernissartensis*) and *Marginocephalia* (*Triceratops horridus*). Note that the internal specifier *Ankylosaurus magniventris* and the external specifier *Triceratops horridus* are not included in the primary reference phylogeny. The former belongs to *Ankylosauria* within *Thyreophora* (see, *e.g*., Thompson et al., 2012), while the latter is part of *Ceratopsia* (*e.g*., Morschhauser et al., 2019).

### *Triceratopsini* Longrich, 2011 (converted clade name)

**Registration number:** 692

**Definition.** The largest clade containing *Triceratops horridus* Marsh, 1889 but not *Anchiceratops ornatus* Brown, 1914c and *Arrhinoceratops brachyops* Parks, 1925. This is a maximum-clade definition. Abbreviated definition: max ∇ (*Triceratops horridus* Marsh, 1889 ~ *Anchiceratops ornatus* Brown, 1914c & *Arrhinoceratops brachyops* Parks, 1925).

**Reference phylogeny.** Figure 9a of Fowler & Freedman Fowler (2020) is treated here as the primary reference phylogeny. Additional reference phylogenies include Figure 11 of Longrich (2011), Figure 3 of Brown & Henderson (2015), Figure 14 of Mallon et al. (2016), and Figure 3 of Campbell et al. (2019).

**Composition.** Under the primary reference phylogeny, *Triceratopsini* comprises *Eotriceratops xerinsularis*, *Nedoceratops hatcheri*, *Ojoceratops fowleri*, *Torosaurus* spp., and *Triceratops* spp.

**Synonyms.** No other taxon names are currently in use for the same or approximate clade.

**Comments.** The name was first (informally) defined by Longrich (2011) who applied the maximum-clade definition and used *Triceratops horridus* as the internal specifier and *Anchiceratops ornatus* and *Arrhinoceratops brachyops* as the external specifiers. We formalize this definition.

### *Tsintaosaurini* Prieto-Márquez et al., 2013 (converted clade name)

**Registration number:** 660

**Definition.** The largest clade containing *Pararhabdodon isonensis* Casanovas-Cladellas, Santafé-Llopis & Isidro-Llorens, 1993 and *Tsintaosaurus spinorhinus* Young, 1958 but not *Aralosaurus tuberiferus* Rozhdestvensky, 1968, *Lambeosaurus lambei* Parks, 1923 and *Parasaurolophus walkeri* Parks, 1922. This is a maximum-clade definition. Abbreviated definition: max ∇ (*Pararhabdodon isonensis* Casanovas-Cladellas, Santafé-Llopis & Isidro-Llorens, 1993 & *Tsintaosaurus spinorhinus* Young, 1958 ~ *Aralosaurus tuberiferus* Rozhdestvensky, 1968 & *Lambeosaurus lambei* Parks, 1923 & *Parasaurolophus walkeri* Parks, 1922).

**Reference phylogeny.** Figure 18 of Prieto-Márquez, Wagner & Lehman (2020) is treated here as the primary reference phylogeny. Additional reference phylogenies include Figure 20 of Xing, Mallon & Currie (2017), Figure 5 of Kobayashi et al. (2019), Figure 11 of Prieto-Márquez et al. (2019), Figure 5 of Zhang et al. (2020), Figure 7 of Kobayashi et al. (2021), and Figure 11 of McDonald et al. (2021).

**Composition.** Under the primary reference phylogeny, *Tsintaosaurini* comprises *Pararhabdodon isonensis* and *Tsintaosaurus spinorhinus*.

**Synonyms.** No other taxon names are currently in use for the same or approximate clade.

**Comments.** The name was first (informally) defined by Prieto-Márquez et al. (2013) who applied the minimum-clade definition and used *Pararhabdodon isonensis* and *Tsintaosaurus spinorhinus* as the internal specifiers. We preserve the original intent of Prieto-Márquez et al. (2013) but prefer to use the maximum-clade definition. *Pararhabdodon isonensis* and *Tsintaosaurus spinorhinus* are used as the internal specifiers and representatives of *Aralosaurini* (*Aralosaurus tuberiferus*), *Lambeosaurini* (*Lambeosaurus lambei*), and *Parasaurolophini* (*Parasaurolophus walkeri*), as the external specifiers. The name *Tsintaosaurini* is inapplicable under some recent phylogenies (Prieto-Márquez et al., 2019; Gates, Evans & Sertich, 2021; Longrich et al., 2021).

## Discussion

Phylogeny reconstructions of some ornithischian clades currently face challenges that have an impact on the construction of the phylogenetic definitions of several taxon names. Below, we provide discussion of some topological conflicts.

### The phylogeny of early-diverging ornithischians

The early evolution of *Ornithischia* and the phylogenetic relationships of taxa nested near the base of the clade are currently contentious, particularly with respect to the potential Triassic members of the clade. Ornithischians have been ‘traditionally’ represented by a single undisputed Triassic taxon, *Pisanosaurus mertii* Casamiquela, 1967. Recent reassessments of the type specimen of *P. mertii* showed, however, that the morphological features of the taxon are rather difficult to interpret and that it may represent either a non-dinosaur dinosauriform from the clade *Silesauridae* (Agnolín & Rozadilla, 2018, Baron, 2019) or an early-diverging ornithischian (Desojo et al., 2020).

Even if *P. mertii* turns out to be a silesaurid, however, it may still represent an early-diverging ornithischian dinosaur as a few studies have proposed that silesaurids, a group of Anisian–?Rhaetian (Middle and Late Triassic) dinosauriforms that are usually inferred to be the sister group to dinosaurs (*e.g*., Nesbitt et al., 2010; Peecook et al., 2013; Ezcurra, 2016; Cau, 2018; Ezcurra et al., 2020), may form an early clade of ornithischians (Langer & Ferigolo, 2013; Cabreira et al., 2016; Pacheco et al., 2019) or a paraphyletic assemblage of early-diverging ornithischians (Müller & Garcia, 2020). Such placement of the silesaurid taxa, especially the oldest known members referred to the group, would have considerable implications for the early evolution of dinosaurs as a whole because neither of the two other major dinosaur clades, *Theropoda* and *Sauropodomorpha*, are known from the Middle Triassic.

Pending additional studies more focused on the basal dinosauriform-dinosaur transition, we do not define neither *Silesauridae* Langer et al., 2010 nor the recently proposed name *Sulcimentisauria* Martz & Small, 2019. If formal definitions for the names are to be proposed in the future, the definitions should comply with all recently proposed phylogenies, including the possible paraphyletic ‘dissolution’ of *Silesauridae* (Müller & Garcia, 2020) that would make *Sulcimentisauria*, as (informally) defined by Martz & Small (2019), applicable to a clade containing the vast majority of ‘traditional’ silesaurids and all ‘core’ ornithischians. One option is to restrict the use of *Sulcimentisauria* for a clade only when inferred within *Silesauridae* (*e.g*., ‘max ∇ ∈ *Silesauridae* (*Silesaurus opolensis* Dzik, 2003 ~ *Asilisaurus kongwe* Nesbitt et al., 2010)’), as originally intended by Martz & Small (2019).

Recently, Baron & Barrett (2017, 2018) reconstructed the enigmatic dinosaur *Chilesaurus diegosuarezi* from the Tithonian (uppermost Jurassic) of Central Patagonian Cordillera in Chile to represent the earliest-diverging ornithischian, which was in striking contrast to the original inference of the taxon at the base of *Tetanurae*, within *Theropoda* (Novas et al., 2015). Although the proposed placement of *Chilesaurus* among early-diverging ornithischians does not have any impact on the use of particular clade names (except when the extent of *Ornithischia* is to be indicated on some recently inferred phylogenies; see Fig. 3), it is perhaps appropriate to express some skepticism towards this inference. As already noted by Müller et al. (2018), the originally proposed tetanurine affinities have not been tested by Baron & Barrett (2017, 2018), nor by Baron (2019), or Müller & Dias-da-Silva (2019), all of whom have also reconstructed *C. diegosuarezi* at the earliest-diverging position within *Ornithischia*. It is worth noting that Dieudonné et al. (2020) included *C. diegosuarezi* in their data matrix as well; however, despite being considered an ornithischian by the authors, its placement at the very base of their tree does not indicate ornithischian affinities for the taxon. Dieudonné et al. (2020) did not include any theropods and/or sauropodomorphs in their analysis and, as such, they did not explore the placement of *C. diegosuarezi* among dinosaurs. In turn, the studies of Baron & Barrett (2017, 2018), Baron (2019), and Müller & Dias-da-Silva (2019) have all been based on a dataset modified from the one first published by Baron, Norman & Barrett (2017a) that was constructed to test the relationships of rootward dinosaurs (ornithischians, theropods, and sauropodomorphs), especially Late Triassic and Early Jurassic forms (though some younger taxa of *Ornithischia* were included as well) and their closest pan-avian relatives. We are of the opinion that, at present and with the evidence provided, the placement of *Chilesaurus*, a latest Jurassic taxon with mosaic features, at the very base of a clade that originated in the Triassic or at the Triassic/Jurassic boundary interval, may be best interpreted as being indicative of inadequate/inappropriate data sampling. In other words, the dataset of Baron, Norman & Barrett (2017a) and its more recent versions are most likely unable to actually test the phylogenetic placement of *Chilesaurus*.

### The phylogenetic placement of *Heterodontosauridae*

The members of *Heterodontosauridae* have long been treated as early-diverging ornithopods (*e.g*., Sereno, 1986, 1998, 1999). The last two decades have shown, however, that heterodontosaurids represent some of the more problematic ornithischian groups, with some studies inferring them as non-ornithopod neornithischians (Butler, 2005), as the sister group to *Marginocephalia* (Xu et al., 2006), near the base of *Ornithischia* (*e.g*., Butler, Upchurch & Norman, 2008; Boyd, 2015; Sereno, 2012; Dieudonné et al., 2016; Han et al., 2018; Madzia, Boyd & Mazuch, 2018; Andrzejewski, Winkler & Jacobs, 2019; Herne et al., 2019; Yang et al., 2020), and within *Pachycephalosauria* (Dieudonné et al., 2020). With respect to the recent reconstruction of heterodontosaurids as early-diverging pachycephalosaurs by Dieudonné et al. (2020), it is worth noting that *Heterodontosauridae* still form a clade (*contra* Dieudonné et al., 2020). Even though some taxa that are usually inferred as members of *Heterodontosauridae* (*Echinodon becklesii* and *Tianyulong confuciusi*) are placed more closely to pachycephalosaurids in Dieudonné et al. (2020: Fig. 1) than to *Heterodontosaurus*, making the ‘traditional’ composition of the group as inferred in other recent studies paraphyletic, *Heterodontosauridae* still comprises *Abrictosaurus consors*, *Fruitadens haagarorum*, *Heterodontosaurus tucki*, and *Lycorhinus angustidens* in that study. Similarly, under the topology of Xu et al. (2006), heterodontosaurids and marginocephalians were inferred as the sister taxa, forming a clade named *Heterodontosauriformes*. Such topology has not been supported in more recent studies (see studies cited above).

Regardless of which of the hypotheses will gain further support in subsequent studies, the definition of the name *Heterodontosauridae* needs to reflect each of them. Therefore, the applied phylogenetic definition of the name includes representatives of all major ornithischian lineages, *Ceratopsia* (*Triceratops horridus*), *Ornithopoda* (*Iguanodon bernissartensis*), *Pachycephalosauria* (*Pachycephalosaurus wyomingensis*), and *Thyreophora* (*Stegosaurus stenops*).

### The early-diverging thyreophorans and ankylosaurs

The ‘armored’ dinosaurs, *Thyreophora*, comprise two major clades, *Ankylosauria* and *Stegosauria*, and other taxa that are more closely related to members of the two species-rich lineages than to ornithopods and marginocephalians. These include *Emausaurus ernsti*, *Scelidosaurus harrisonii*, and *Scutellosaurus lawleri* (Han et al., 2018; Herne et al., 2019; Madzia, Boyd & Mazuch, 2018; Dieudonné et al., 2020), and some other, more problematic taxa, such as the dubious ‘*Tatisaurus oehleri*’ (Norman, Butler & Maidment, 2007) and ‘*Bienosaurus lufengensis*’ (Raven et al., 2019). *Lesothosaurus diagnosticus* and *Laquintasaura venezuelae* have been inferred as early-diverging thyreophorans as well (see, *e.g*., Butler, Upchurch & Norman, 2008 for the placement of *L. diagnosticus*, and, *e.g*., Baron, Norman & Barrett, 2017c and Andrzejewski, Winkler & Jacobs, 2019 for the position of *La. venezuelae*). Other studies, however, place *Le. diagnosticus* as an early neornithischian (Madzia, Boyd & Mazuch, 2018; Herne et al., 2019) or an early-diverging ornithischian in general (Han et al., 2018; Andrzejewski, Winkler & Jacobs, 2019; Dieudonné et al., 2020; Yang et al., 2020), and *La. venezuelae* as an early-diverging ornithischian (Han et al., 2018; Dieudonné et al., 2020; Yang et al., 2020).

Following his thorough redescription of *Scelidosaurus harrisonii* (Norman, 2020a, 2020b, 2020c), Norman (2021) assessed the phylogenetic relationships of early-diverging thyreophorans and reconstructed *E. ernsti*, *Sce. harrisonii*, and *Scu. lawleri* as the earliest-diverging ankylosauromorphs (*Ankylosauria sensu* this study), restricting the name *Ankylosauria* to a smaller clade, approximately comprising ankylosaurids and nodosaurids (two definitions – one minimum-clade and one maximum-clade – were provided; both applying the name to the same known contents). Norman (2021: 70) further noted that the node comprising ankylosaurids and nodosaurids “has the potential to become the new taxon Euankylosauria but this additional clade name is neither essential nor particularly desirable”.

When applying a minimum-clade definition (*e.g*., ‘min ∇ (*Ankylosaurus magniventris* Brown, 1908 & *Nodosaurus textilis* Marsh, 1889)’), the name *Euankylosauria* may indeed be useful in the future, especially if further studies support the placement of some taxa, such as *Mymoorapelta maysi* and *Kunbarrasaurus ieversi* (as in Arbour & Currie, 2016), or *E. ernsti*, *Sce. harrisonii*, and *Scu. lawleri* (as in Norman, 2020c), as non-ankylosaurid/non-nodosaurid ankylosaurs. However, there is no need to replace *Ankylosauria* with *Ankylosauromorpha* as the name for the largest clade containing *A. magniventris* but not *Stegosaurus stenops*. The branch has long been named *Ankylosauria* and it has always been expected that it may contain taxa with characters that are absent in ‘traditional’ ankylosaurs (*i.e*., ankylosaurids and nodosaurids). We suggest that the name *Ankylosauromorpha* is abandoned.

### Problematic clades within *Ankylosauria*

Comprehensive alpha taxonomic reviews and phylogenetic analyses of *Ankylosauridae* in recent years have clarified many of the interrelationships within this clade (*e.g*., Arbour & Currie, 2013; Arbour & Currie, 2016). However, similar reviews for *Nodosauridae* have not been undertaken in recent years, and phylogenetic resolution within *Nodosauridae* is often poor and inconsistent between different phylogenies (*e.g*., Thompson et al., 2012; Arbour, Zanno & Gates, 2016; Brown et al., 2017), in part because many recent ankylosaur phylogenetic analyses are modified from Arbour & Currie (2016) which was designed to test relationships within *Ankylosauridae*, not *Nodosauridae*. Additionally, many names for clades within *Nodosauridae* have been introduced by various authors based on proposed diagnostic characters rather than phylogenetic hypotheses, and have not been defined phylogenetically. In particular, the validity of *Polacanthidae* or *Polacanthinae*, *Sauropeltinae*, *Struthiosaurinae*, and *Stegopeltinae*, and the contents of *Edmontiniinae* or *Panoplosaurinae*, are unclear. In this manuscript we provide a formal definition of *Polacanthinae*, and discuss the use of *Struthiosaurinae* and *Panoplosaurinae*, as the names have been mentioned recently with some frequency and have had informal definitions proposed previously. Ford (2000) introduced the names *Sauropeltinae* and *Stegopeltinae* and provided diagnostic characters but did not test their contents phylogenetically; *Sauropeltinae* included *Sauropelta edwardsorum* and *Silvisaurus condrayi* and *Stegopeltinae* included *Aletopelta coombsi*, *Glyptodontopelta mimus*, and *Stegopelta landerensis*. *Sauropelta* and *Silvisaurus* do not form a clade in any recent analyses, nor do *Stegopelta*, *Glyptodontopelta*, and *Aletopelta*. As such, we do not provide formal definitions for *Sauropeltinae* or *Stegopeltinae* at this time.

### The origin of *Ornithopoda*

The understanding of the origin and early evolution of *Ornithopoda* is tightly connected with the knowledge of the character distribution among rootward neornithischians. With that respect, the basal neornithischian-ornithopod transition is among the poorest known stages of the ornithischian evolutionary history, as recent phylogenetic studies that focused on that particular tree segment provided strikingly conflicting topologies (Boyd, 2015; Dieudonné et al., 2016; Han et al., 2018; Madzia, Boyd & Mazuch, 2018; Andrzejewski, Winkler & Jacobs, 2019; Herne et al., 2019; Dieudonné et al., 2020; Yang et al., 2020).

Substantial conflicts are apparent especially with regards to the phylogenetic placements of taxa ‘traditionally’ dubbed the ‘hypsilophodonts’ (compare, *e.g*., Boyd, 2015; Han et al., 2018; Herne et al., 2019), including *Hypsilophodon foxii* itself (*e.g*., Madzia, Boyd & Mazuch, 2018). Phylogeny reconstructions of ornithopods provide more stable results around the node marking the origin of *Iguanodontia* (*e.g*., Madzia, Boyd & Mazuch, 2018; Madzia, Jagt & Mulder, 2020), although alternative hypotheses of early iguanodontian phylogenetic relationships exist as well (*e.g*., Norman, 2015). The names of non-cerapod neornithischian and rootward ornithopod clades are defined here to reflect these uncertainties though we recognize that some potential topologies may still render issues. For example, if *Hypsilophodon* forms a clade with thescelosaurids but falls outside the *Thescelosaurus* + *Orodromeus* node, *Hypsilophodontidae* would cover *Thescelosauridae* if the latter name was defined using a minimum-clade definition (as in Brown et al., 2013 and Madzia, Boyd & Mazuch, 2018). We do not include *T. neglectus* as an external specifier in the definition of *Hypsilophodontidae* because under the scenario, in which *H. foxii* would be inferred within the *Thescelosaurus* + *Orodromeus* node, the names *Thescelosauridae*, *Thescelosaurinae*, and *Orodrominae* would be all inapplicable, while *Hypsilophodontidae* could effectively remain in use only for *H. foxii*. The definitions we propose ensure that if *H. foxii* is component of the *Thescelosaurus* + *Orodromeus* clade, *Thescelosauridae* becomes inapplicable, while *Thescelosaurinae* and *Orodrominae* still remain in use. The potential issue with *Hypsilophodontidae* covering *Thescelosauridae* under a topology in which *Hypsilophodon* is the sister taxon to the *Thescelosaurus* + *Orodromeus* node was solved by providing *Thescelosauridae* with a maximum-clade definition that makes it inapplicable under such scenario.

### Hadrosaurid ingroup relationships

Hadrosaurids are some of the most intensively researched ornithischians, with thoroughly explored phylogenetic relationships. Recent studies almost uniformly infer seven major hadrosaurid clades: *Brachylophosaurini*, *Edmontosaurini*, *Kritosaurini*, *Lambeosaurini*, *Parasaurolophini*, *Saurolophini*, and *Tsintaosaurini* (*e.g*., Freedman Fowler & Horner, 2015; Prieto-Márquez, Erickson & Ebersole, 2016; Xing, Mallon & Currie, 2017; Kobayashi et al., 2019; Prieto-Márquez et al., 2019; Prieto-Márquez, Wagner & Lehman, 2020; Zhang et al., 2020; Kobayashi et al., 2021; Longrich et al., 2021; McDonald et al., 2021; Ramírez-Velasco et al., 2021). Longrich et al. (2021) recently introduced a new clade name, *Arenysaurini*, for a diverse grouping of mostly European lambeosaurines, resulting, at the same time, in *Tsintaosaurini* (as originally used and as defined here) becoming inapplicable. The study of Longrich et al. (2021) was first to infer such topology. Other phylogenetic studies placed *Arenysaurus ardevoli* either deeply within *Lambeosaurini* (*e.g*., Prieto-Márquez, Erickson & Ebersole, 2016; Prieto-Márquez et al., 2019; Zhang et al., 2019; Prieto-Márquez, Wagner & Lehman, 2020; Gates, Evans & Sertich, 2021; Ramírez-Velasco et al., 2021), within *Parasaurolophini* (Cruzado-Caballero et al., 2013), or as the sister taxon or close to the clade uniting *Lambeosaurini* and *Parasaurolophini* (*e.g*., Pereda-Suberbiola et al., 2009; Cruzado-Caballero, Pereda-Suberbiola & Ruiz-Omeñaca, 2010; Godefroit, Bolotsky & Bolotsky, 2012; Cruzado-Caballero & Powell, 2017; Xing, Mallon & Currie, 2017; Kobayashi et al., 2019; Zhang et al., 2020).

Owing to the fact that the consensus regarding the placement of *Arenysaurus ardevoli* among lambeosaurines has yet to be reached, and that other ‘arenysaurins’ of Longrich et al. (2021) are distributed across the lambeosaurine tree in other studies, we do not define *Arenysaurini* here. If future studies support the results of Longrich et al. (2021), *Arenysaurini* should probably be defined so that it becomes inapplicable if inferred within *Lambeosaurini*. The easiest way to do so would be to define *Arenysaurini* through a maximum-clade definition using *Arenysaurus ardevoli* and at least one other internal specifier that would make the name applicable only in the case *Arenysaurus* is inferred outside *Lambeosaurini*. The taxon *Adynomosaurus arcanus* is a possible candidate, if such a solution is preferred. In turn, *Blasisaurus canudoi* should be avoided as this taxon has been inferred as the sister taxon of *A. ardevoli* in some analyses (*e.g*., Cruzado-Caballero, Pereda-Suberbiola & Ruiz-Omeñaca, 2010; Cruzado-Caballero et al., 2013; Prieto-Márquez et al., 2019; Prieto-Márquez, Wagner & Lehman, 2020; Gates, Evans & Sertich, 2021). Another option is to apply a clause similar to that used in the definitions of *Clypeodonta*, *Euornithopoda*, *Hypsilophodontia*, *Orodrominae*, and *Thescelosaurinae*. That is, by using the set theory symbol ∉, meaning “not element of”, the name *Arenysaurini* could be applicable only under the condition that the clade for which the name was intended was reconstructed outside *Lambeosaurini* and *Parasaurolophini*. Such definition could be abbreviated as follows: max ∇ ∉ *Lambeosaurini* & *Parasaurolophini* (*Arenysaurus ardevoli* Pereda-Suberbiola et al., 2009 ~ *Lambeosaurus lambei* Parks, 1923 & *Parasaurolophus walkeri* Parks, 1922).

## Conclusions

Ornithischian dinosaurs were a major clade of globally distributed Mesozoic archosaurs that achieved substantial taxic diversity and apparent morphological disparity, expressed especially through their cranial features and the body armor of some of their most distinctive members. Throughout their two-century-long research history, ornithischians have been thoroughly assessed both taxonomically and phylogenetically, which has led to the recognition of numerous clades.

Following the pivotal studies establishing the theoretical foundation of the phylogenetic nomenclature in the 1980s and early 1990s, many names for the ornithischian clades have been provided phylogenetic definitions, some of which have proven useful and have not been changed since their introduction.

However, following the 2020 establishment of the *International Code of Phylogenetic Nomenclature* (*ICPN*), or the *PhyloCode*, all of the definitions proposed before the implementation of the Code are treated as formally ineffective.

We have reconsidered the utility of previously proposed phylogenetic definitions of established ornithischian taxon names and provide definitions for 81 names of ornithischian clades, five of which are newly proposed here, as specified by the Articles of the *ICPN*, thus marking a key step towards a formal phylogenetic nomenclature of ornithischian dinosaurs.

## Institutional abbreviations

CMN: Canadian Museum of Nature, Ottawa, Ontario, Canada
CPC: Colección Paleontológica de Coahuila, Museo del Desierto, Saltillo, Mexico
GPDM: Great Plains Dinosaur Museum, Malta, Montana, USA
MOR: Museum of the Rockies, Bozeman, Montana, USA
PASAC: Paleontological Association of Sabinas, Coahuila, Mexico
ROM: Royal Ontario Museum, Toronto, Ontario, Canada
UTEP: Centennial Museum and Chihuahuan Desert Gardens, University of Texas at El Paso, Texas, USA
ZPAL: Institute of Paleobiology, Polish Academy of Sciences, Warsaw, Poland

## Additional Information and Declarations

### Data Availability

The following information was supplied regarding data availability: This study did not generate any raw data.
